# Global Burden and Improvement Gap of Non-Rheumatic Calcific Aortic Valve Disease: 1990–2019 Findings from Global Burden of Disease Study 2019

**DOI:** 10.3390/jcm11226733

**Published:** 2022-11-14

**Authors:** Chengzhi Yang, Haobo Xu, Ruofei Jia, Zening Jin, Changlin Zhang, Jiansong Yuan

**Affiliations:** 1 Department of Cardiology and Macrovascular Diseases, Beijing Tiantan Hospital, Capital Medical University, Beijing 100070, China; 2 Department of Cardiology, Fuwai Hospital, National Center for Cardiovascular Diseases, Chinese Academy of Medical Sciences and Peking Union Medical College, Beijing 100037, China; 3 Department of Cardiology, the Second Affiliated Hospital of Dalian Medical University, Dalian 116023, China

**Keywords:** non-rheumatic calcific aortic valve disease (nrCAVD), global burden of disease, prevalence, disability-adjusted life-years (DALYs), socio-demographic index, contribution factor, improvement

## Abstract

The aim of this study was to explore the most updated changing trends of non-rheumatic calcific aortic valve disease (nrCAVD) and reveal possible improvements. We analyzed the age-standardized rates (ASRs) of prevalence, incidence, disability-adjusted life-years (DALYs), and mortality trends of nrCAVD from 1990 to 2019 using data from the Global Burden of Disease (GBD) study 2019. The relations between ASRs and socio-demographic index (SDI) were analyzed with Pearson’s correlation coefficients. Decomposition and frontier analysis were employed to reveal the contribution proportion of influence factors and regions where improvement can be achieved. In 2019, there were 9.40 million (95% uncertainty interval (UI): 8.07 to 10.89 million) individuals with nrCAVD globally. From 1990 to 2019, the prevalence rate of nrCAVD increased by 155.47% (95% IU: 141.66% to 171.7%), with the largest increase observed in the middle SDI region (821.11%, 95% UI: 709.87% to 944.23%). Globally, there were no significant changes in the mortality rate of nrCAVD (0.37%, 95% UI: −8.85% to 7.99%). The global DALYs decreased by 10.97% (95% UI: −17.94% to −3.46%). The population attributable fraction (PAF) of high systolic blood pressure increased in the population aged 15–49 years, while it declined slightly in population aged 50+ years. Population growth was the main contributing factor to the increased DALYs across the globe (74.73%), while aging was the driving force in the high-SDI region (80.27%). The Netherlands, Finland, Luxembourg, Germany, and Norway could reduce DALY rates of nrCAVD using their socio-demographic resources. According to these results, we revealed that the burden of nrCAVD increased markedly from 1990 to 2019 in high-SDI and high-middle-SDI regions. There was a downward trend in the mortality due to nrCAVD since 2013, which is possibly owing to profound advances in transcatheter aortic valve replacement. Some countries may reduce burdens of nrCAVD using their socio-demographic resources.

## 1. Introduction

The 20th anniversary of transcatheter aortic valve replacement (TAVR) has seen profound advances since its appearance in 2002, owing to dramatically increased patients with aortic valve stenosis [1]. Several studies have demonstrated that non-rheumatic calcific aortic valve disease (nrCAVD) is the main pathological basis of aortic valve stenosis nowadays [2,3]. The past three decades witnessed rapid economic and social developments, with increased burden of non-communicable diseases. As one of these disease burdens, the prevalence of nrCAVD increased markedly and carried a significant risk of mortality and morbidity in the global population [4,5,6]. In a substudy of the SCOT-HEART multicenter randomized controlled trial, the authors showed that aortic valve calcification was observed in 241 (14%) of the 1769 participants aged 58 ± 9 years [7]. In addition, a meta-analysis including 22 studies found that the prevalence of aortic sclerosis ranged from 9% in a study where the mean age was 54 years to 42% in a study where the mean age was 81 years [8]. However, the studies concerning the burden of nrCAVD across the globe are very limited. Although Simon Yadgir et al. reported the general global changes of non-rheumatic valvular diseases from 1990 to 2017 [9], the global burden of nrCAVD was not investigated in detail, particularly the influence factors of nrCAVD epidemiology and potential improvement gap.

In the present study, we sought to explore the most updated changing trends of nrCAVD with the Global Burden of Disease (GBD) study 2019 data. Particularly, we used decomposition and frontier analysis to reveal the contribution proportion of influence factors and regions where improvement can be possibly achieved.

## 2. Methods

### 2.1. Data Sources and Case Definitions

We used data from the Global Burden of Diseases, Injuries, and Risk Factors Study (GBD) 2019, which was designed to provide a comprehensive assessment of health loss due to diseases, causes of death, and risk factors at the global, regional, and national levels from 1990 to 2019. The non-rheumatic calcific aortic valve disease (nrCAVD) was defined based on the International Classification of Diseases Ninth Revision (ICD-9) and Tenth Revision (ICD-10) codes.

### 2.2. Socio-Demographic Index (SDI)

The socio-demographic index (SDI) was employed to determine the relationship between the development status of a region or country and the burden of nrCAVD. The SDI is a composite indicator of social development levels that correlate with health outcomes. It is calculated from national-level income per capita, educational attainment in the population ≥ 15 years old, and women fertility rate under 25 years old. The SDI ranges from 0 (minimum development) to 1 (maximum development), and 204 countries and territories were categorized into five groups based on SDI quintiles: low SDI, low-middle SDI, middle SDI, high-middle SDI, and high SDI.

### 2.3. Estimation of Prevalence, Incidence, and Disability-Adjusted Life-Years

The prevalence and incidence of nrCAVD was estimated using DisMod-MR 2.1, a Bayesian meta-regression tool by which GBD 2019 collected and analyzed data from hospital discharges, publications, and household surveys. The disability-adjusted life-years (DALYs) is calculated by adding the years of life lost (YLLs) due to premature death and the years lost due to disability (YLDs) in the population.

The age-standardized rates (ASRs) of prevalence, incidence, deaths, and DALYs were generated by summarizing the products of the age-specific rates and corresponding number of persons in the same age subgroup of the GBD 2019 standard population, and then dividing by the sum of the standard population weights. The changes of ASRs between 1990 and 2019 were shown with percentage changes. Uncertainty intervals (UIs) were calculated from 1000 draws for each quantity. The 95% UIs were defined as the 25th and 975th ordered draw of the uncertainty distribution. For all analysis, a 95% UI and 95% confidence intervals (CIs) were considered to be statistically significant when zero was excluded.

### 2.4. Risk Factors for DALYs

The inclusion criteria of attributable risk factors were previous evidence of causation with nrCAVD and availability of exposure data in GBD 2019. The final risk factors included were high systolic blood pressure, diet high in sodium, and lead exposure.

### 2.5. Statistical Analysis

Spearman’s rank order correlation was used to measure the strength and direction of the association between the SDI and age-standardized rates. The change in the SDI between 1990 and 2019 (the ratio of the index in 2019 to the index in 1990), and the average annual percentage change (AAPC) of ASR during 1990–2019 was calculated. To assess the magnitude and direction of trends in the ASR of nrCAVD over time, we used JoinPoint software (Version 4.7.0.0) to calculate the AAPC and the corresponding 95% confidence interval (CI) by joinpoint regression analysis. By comparing AAPC with 0, we ascertained whether the variation trend in different sections is statistically significant.

The decomposition analysis is an analytic approach to identify the additive contribution of the effect of the differences in factors in 2 populations (such as the population in 1990 and the population in 2019) on the difference in their overall value. The decomposition of nrCAVD DALYs by the different causes allows the quantification of the contribution of each cause to the overall nrCAVD DALYs. We first used the decomposition methodology of Das Gupta to decompose nrCAVD DALYs by population age structure, population growth, and epidemiologic changes (DALYs rate) [10,11]. The number of DALYs at each location was obtained from the following formula:

(1) $${\mathrm{DALY}\;}_{\mathrm{ay},\;\mathrm{py},\;\mathrm{ey}}=\sum_{i=1}^{20}({a\;}_{i,\;y}*{p\;}_{y}*{e\;}_{i,\;y})$$

where DALY _ay, py, ey_ represent DALYs based on the factors of age structure, population, and DALYs rate for specific year y; a _i, y_ represents the proportion of population for the age category i of the 20 age categories in given year y; p _y_ represents the total population in given year y; and e _i, y_ represents DALYs rate given age category i in year y. The contribution of each factor to the change in DALYs from 1990 to 2019 was defined by the effect of one factor changing while the other factors were held constant.

For example, the effect of age structure was calculated as follows:

[(DALY _a2019, p1990, e1990_ + DALY _a2019, p2019, e2019_)/3 + (DALY _a2019, p1990, e2019_ + DALY _a2019, p2019, e1990_)/6] − [(DALY _a1990, p2019, e2019_ + DALY _a1990, p1990, e1990_)/3 + (DALY _a1990, p2019, e1990_ + DALY _a1990, p1990, e2019_)/6] (2)

In order to evaluate the relationship between the burden of nrCAVD and socio-demographic development, we applied a frontier analysis as a quantitative methodology to identify the lowest potentially achievable age-standardized DALYs rate on the basis of development status as measured by the socio-demographic index (SDI). In this method, data envelopment analysis (DEA) would be used for frontier analysis. The frontier can be produced from several deterministic algorithms, such as free disposability hull (FDH), variable returns to scale (VRS), and so on. We used the FDH in our analysis. To incorporate stochastic variation into the frontier, we used 1000 bootstrapped samples of the data. Each bootstrap includes a subset of locations produced by randomly sampling with replacement from all countries in the Global Burden of Disease study. This accounts for autocorrelation of locations over time. In each bootstrapped sample, the following procedure was performed: Firstly, remove one data point at a time to generate a DEA and identify if the removed point is a superefficient point (outlier). Then, put the data point back, remove the second data point to generate DEA, and examine if it is the superefficient point (outlier). In this method, the superefficient point is defined as the unit whose number of age-standardized DALY rate is less than the frontier line at each SDI value calculated after removing the unit. After all the points are examined and removed all superefficient points (outliers), we then generate the frontier using DEA with FDH algorithm. We repeat this step for 1000 iteration bootstrapping, and the mean nrCAVD DALYs frontier at each SDI value from the bootstrapped samples was computed for each country at each year. Finally, LOESS regression with a local polynomial degree of 1 and span of 0.2 was then developed to generate a smoothed frontier [12,13]. To understand the relationship of age-standardized nrCAVD DALY rates vis-à-vis the frontier in 2019, we calculated the effective difference (the absolute distance from the frontier) using 2019 SDI and age-standardized nrCAVD DALYs rate data point for each country or territory. Countries or territories with lower DALYs than the frontiers were assigned a zero distance.

The detailed description of the frontier analysis is described in the Supplementary Method. All the data analyses were conducted with the R program (Version 4.0.4, R core team).

## 3. Results

### 3.1. Prevalence, Incidence, DALYs, and Mortality of nrCAVD

**Figure 1:**
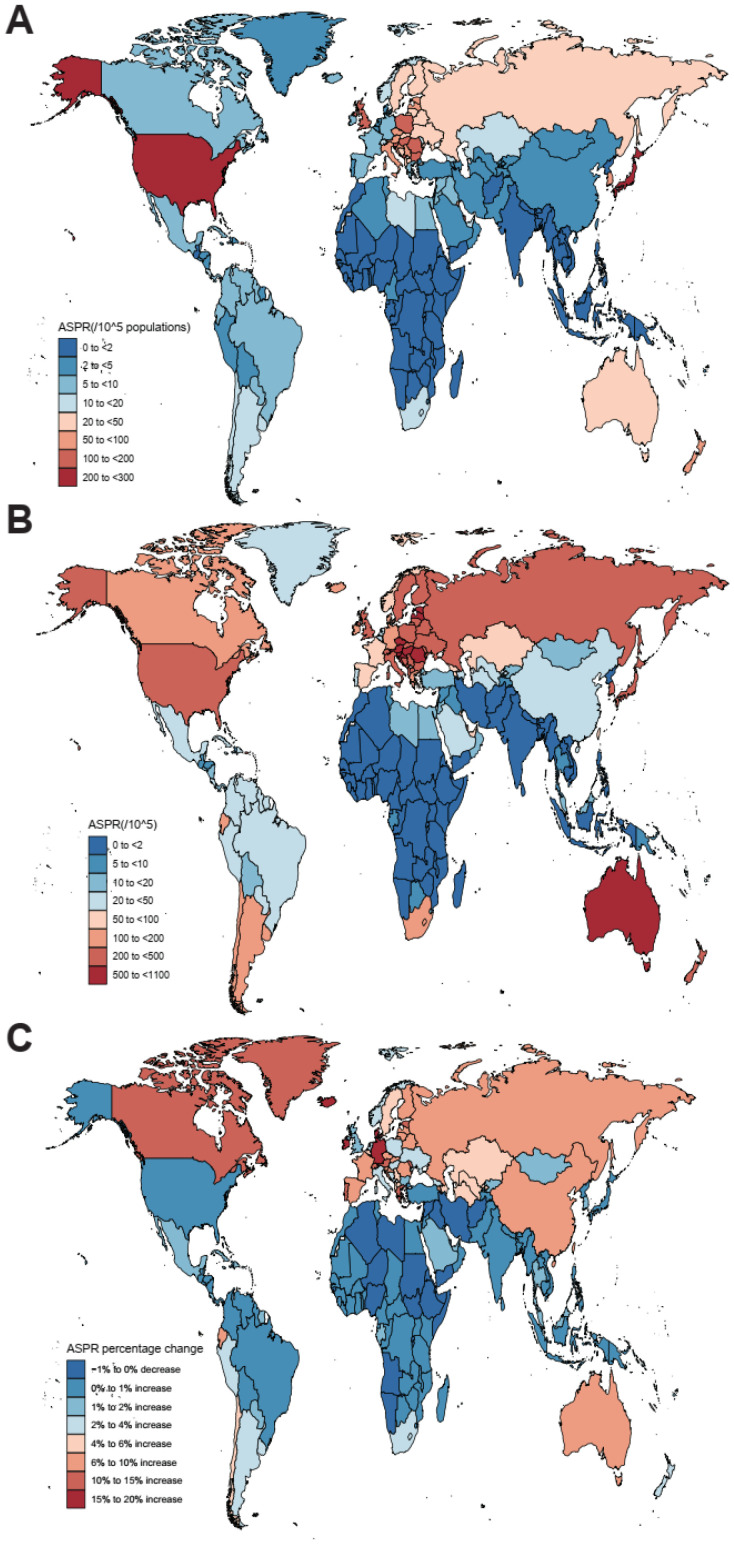
The global age-standardized prevalence rate (ASPR) of non-rheumatic calcific aortic valve disease (nrCAVD) in 204 countries and territories. (**A**) The ASPR of nrCAVD in 1990. (**B**) The ASPR of nrCAVD in 2019. (**C**) The relative change in ASPR of nrCAVD between 1990 and 2019.

**Figure 2:**
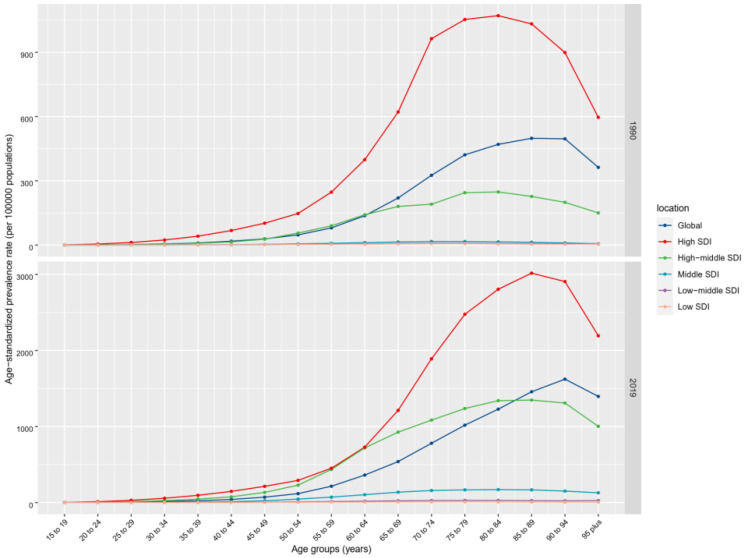
The ASPR of nrCAVD by age group in 1990 and 2017. ASPR, age-standardized prevalence rate; nrCAVD, non-rheumatic calcific aortic valve disease.

Globally, the past 30 years witnessed a marked upward trend in the prevalence of nrCAVD (Table 1, Figure 1; average annual percent change (AAPC) = 3.36 (95% CI: 2.77 to 3.95)). The estimated prevalence cases of nrCAVD increased from 1.73 million (95% UI: 1.43 million to 2.07 million) in 1990 to 9.40 million (95% UI: 8.07 million to 10.89 million) in 2019. At the same time, the age-standardized prevalence rate per 100 000 population (ASPR) increased from 45.54 (95% UI: 37.61 to 54.67) to 116.34 (95% UI: 100.39 to 134.5), indicating a 155.47% (95% UI: 141.66% to 171.7%) augment in ASPR. By sex, the ASPR of nrCAVD in males was higher than that in females (133.38 vs. 99.86 in 2019), whereas the AAPC was comparable between females and males. In terms of age, global prevalence rates of nrCAVD increased with age before 95 years old in both 1990 and 2019. The ASPR of nrCAVD was very low before 30 years old and declined after 95 years old (Figure 2). Regionally, the highest ASPR of nrCAVD was reported in Australasia (649.5, 95% UI: 552.0 to 772.74) and Central Europe (608.31, 95% UI: 517.94 to 713.51) in 2019. In the past 30 years, the largest increase in ASPR was reported in East Asia (1920.64%, 95% UI: 1545.86% to 2360.13%). With respect to countries, the highest ASPR change of nrCAVD was observed in Denmark (2859.7%, 95% UI: 2329.24% to 3473.71%), and the largest number of individuals with nrCAVD was noted in China (867,917, 95% UI: 687,948 to 1,064,921) in 2019 (Supplementary Table S1).

The global incidence number of nrCAVD was 589,638 (95% UI: 512,895 to 677,062) in 2019, with a 350.72% increase from 130,822 (95% UI: 110,701 to 156,022) in 1990 (Supplementary Table S2). Besides, the age-standardized incidence rates per 100,000 population (ASIR) increased from 3.25 (95% UI: 2.76 to 3.86) to 7.13 (95% UI: 6.22 to 8.15), showing an increase of 119.24 (95% UI: 108.72 to 131.69). In 2019, the highest ASIR was reported in Australasia (44.39, 95% UI: 38.08 to 51.79) and Central Europe (33.16, 95% UI: 28.29 to 38.67). The greatest increase in ASIR was observed in Australasia (698.05%, 95% UI: 597.68% to 830.16%), followed by Eastern Europe (678.78%, 95% UI: 631.44% to 732.11%) and East Asia (665.55%, 95% UI: 544.2% to 807.08%).

As shown in Supplementary Table S3, the global age-standardized DALYs rate (ASDR) of nrCAVD decreased from 26.85 (95% UI: 24.07 to 30.31) per 100,000 person-years in 1990 to 23.9 (95% UI: 21.1 to 26.55) per 100,000 person-years in 2019, showing a reduction of 10.97% (95% UI: −17.94% to −3.46%). The ASDR of nrCAVD varied substantially among different regions. In 2019, the highest ASDR of nrCAVD was reported in Western Europe (51.94, 95% UI: 45.69 to 56.95) while the lowest ASDR was reported in East Asia (4.36, 95% UI: 3.59 to 5.18). Over the past 30 years, the largest decrease in DALYs was found in high-income Asia Pacific (−35.91%, 95% UI: −45.57 to −27.93) while the greatest increase was shown in Eastern Europe (169.8%, 95% UI: 114.91% to 265.33%).

Globally, there were no significant changes in the age-standardized mortality rate (ASMR) per 100,000 population of nrCAVD between 1990 and 2019 (0.37%, 95% UI: −8.85% to 7.99%; Supplementary Table S4), while the deaths caused by nrCAVD increased from 53,298 (95% UI: 47,760 to 59,731) to 126,827 (95% UI: 105,603 to 141,390). In detail, there were two periods when the ASMR of nrCAVD increased, 1990 to 1993 and 2003 to 2013. On the other hand, the ASMR decreased in two periods, 1994 to 2002 and 2013 to 2019.

Stratified by SDI regions and sex, the prevalence, incidence, DALYs, and mortality of nrCAVD correlated positively with SDI for both men and women (Supplementary Figures S1 and S2). Consistently, the highest ASPR, ASIR, ASDR, and ASMR were seen in the high-SDI region. At the same time, the middle-SDI region witnessed the greatest increase in ASPR (821.11%, 95% UI: 709.87% to 944.23%), and the high-middle-SDI region saw the greatest increase in ASIR (298.74%, 95% UI: 283.11% to 316.0%).

### 3.2. Risk Factors for nrCAVD

We studied the trends of population attributable fraction (PAF) of three exposure risk factors for DALYs of nrCAVD, high systolic blood pressure (HSBP), diet high in sodium, and lead exposure over the past 30 years. Globally, the age-standardized PAF for HSBP accounted for 38.02% (95% UI 30.27% to 46.42%) in 1990, and 33.62% (95% UI 25.98% to 42.46%; Supplementary Figure S3) in 2019 of DALYs. The PAF of HSBP declined slightly in population aged 50+ years, while it increased in those aged 15–49 years. Besides, the PAF of diet high in sodium and lead exposure remained at a low level and relatively constant from 1990 to 2019. The PAF trends for these risk factors were similar between men and women at all age groups.

Stratified by SDI regions, the PAF of the three risk factors declined in all age groups in the high-SDI region, except for diet high in sodium in those aged 15–49 years (Supplementary Figure S4). For the high-middle SDI region, the trends of PAF for the three risk factors were similar to the global trends. For other SDI regions, the PAF of HSBP increased in all age groups, while the PAF of diet high in sodium declined in most age groups.

### 3.3. Decomposition of nrCAVD

**Figure 3:**
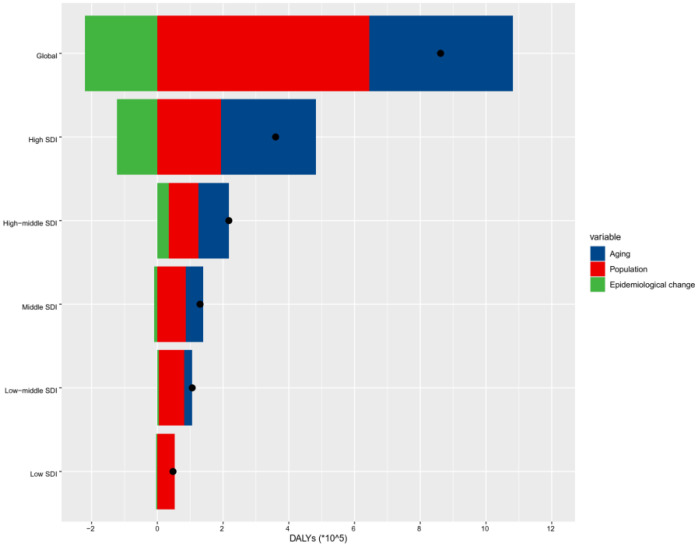
Changes in nrCAVD DALYs according to population-level determinants of population growth, aging, and epidemiological change from 1990 to 2019 at the global level and by SDI quintile. The black dot represents the overall value of change contributed by all three components. For each component, the magnitude of a positive value indicates a corresponding increase in nrCAVD DALYs attributed to the component, and the magnitude of a negative value indicates a corresponding decrease in nrCAVD DALYs attributed to the related component. nrCAVD, non-rheumatic calcific aortic valve disease; SDI, socio-demographic index.

In order to identify the contribution of population growth, aging, and epidemiological changes to the trends of nrCAVD epidemiology over the past three decades, we conducted a decomposition analysis of raw DALYs by age structure, population growth, and epidemiological changes (referring to age- and population-standardized mortality rates). The raw DALYs of nrCAVD were increased in all SDI quintiles, and the increase extent of DALYs was positively related to SDI values. Globally, the population growth contributed 74.73% to the increased burden of nrCAVD DALYs from 1990 and 2019, while the epidemiological changes contributed 25.49% to the decreased burden of nrCAVD DALYs (Figure 3, Supplementary Table S5). The contribution of aging to raw DALYs was highest in the high-SDI region (80.27%); decreased to 42.56% in the high-middle region, 41.2% in the middle region, 23.77% in the low-middle region; and was counterproductive in the low-SDI region (−1.87%). Over the same period, the contribution of population growth showed a nearly contrary trend to that of aging. Of note, although the contribution of epidemiological changes was decreased in most GBD regions, it increased in Central Europe and Eastern Europe, where it contributed 69.83% and 82.86% to the burden of nrCAVD DALYs, respectively.

### 3.4. Frontier Analysis of nrCAVD

**Figure 4:**
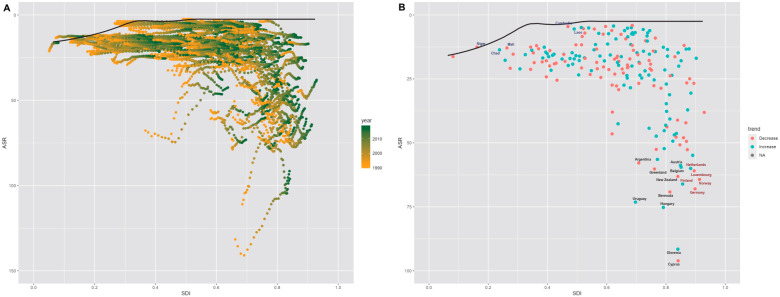
(**A**) Frontier analysis based on SDI and age-standardized nrCAVD DALYs rate from 1990 to 2019. The frontier is delineated in solid black color; countries and territories are represented as dots. (**B**) Frontier analysis based on SDI and age-standardized nrCAVD DALYs rate in 2019. The top 10 countries with the largest effective difference (largest nrCAVD DALYs gap from the frontier) are labeled in black; examples of frontier countries with low SDI (<0.5) and low effective difference are labeled in blue (e.g., Chad, Niger, Mali, Cambodia, and Laos); and examples of countries and territories with high SDI (>0.85) and relatively high effective difference for their level of development are labeled in red (e.g., Netherlands, Finland, Luxembourg, Germany, and Norway). Red dots indicate an increase in age-standardized nrCAVD DALYs rate from 1990 to 2019; blue dots indicate a decrease in age-standardized nrCAVD DALYs rate between 1990 and 2019. DALYs, disability-adjusted life years; SDI, socio-demographic index; nrCAVD, non-rheumatic calcific aortic valve disease.

Furthermore, we performed a frontier analysis based on age-standardized DALY rates (ASDR) of nrCAVD and SDI values to explore the possible improvement in the ASDR that is potentially realized given a nation’s development status. The countries and territories with lowest ASDR (optimal performers) based on corresponding SDI values were indicated by the frontier line. The distance from the frontier line is the gap between a country’s observed and potentially achievable DALYs. This distance is called effective difference. The effective difference could be potentially reduced or eliminated taking the country’s socio-demographic resources. Globally, the effective difference for a given SDI tended to be larger and more variable as SDI increased (Figure 4, Supplementary Table S6). The top 10 countries with the largest effective difference were Cyprus, Slovenia, Hungary, Uruguay, Bermuda, New Zealand, Greenland, Belgium, Austria, and Argentina. Besides, there were some countries and territories with high SDI (>0.85) but relatively high effective difference, such as Netherlands, Finland, Luxembourg, Germany, and Norway. By contrast, several countries with low SDI (<0.5) showed small effective difference, including Chad, Niger, Mali, Cambodia, and Laos.

## 4. Discussion

The non-rheumatic calcific aortic valve disease (nrCAVD) has attracted more and more attention over the past 30 years. In this study, based on the most updated Global Burden of Disease (GBD) study data, we found that the global age-standardized prevalence and incidence rate of nrCAVD increased markedly from 1990 to 2019, while there were no overt changes in the age-standardized DALYs and mortality due to nrCAVD. The population attributable fraction (PAF) of high systolic blood pressure declined slightly in population aged 50+ years, while it increased in those aged 15–49 years. The population growth was the main contributing factor to the increased DALYs across the globe, while aging was the driving force in the high-SDI region. Netherlands, Finland, Luxembourg, Germany, and Norway could reduce DALY rates of nrCAVD using their socio-demographic resources.

Previous studies showed that the prevalence of nrCAVD increased obviously in some countries or regions over the past three decades [8,14,15]. In line with prior reports, we found that both prevalence cases and age-standardized prevalence rate (ASPR) of nrCAVD increased markedly from 1990 to 2019. In addition, the incidence of nrCAVD also increased over the same period. There are several risk factors related to nrCAVD pathogenesis [16]. First, it is well established that aging is an important stimulus to nrCAVD [17]. A recent study of 944 participants aged ≥65 years reported that overall prevalence of nrCAVD was 22.0%, with 16.7% in individuals aged 65~69 years and 67.0% in individuals aged ≥85 years [18]. We also found that the prevalence of nrCAVD sharply surges with advancing age. Interestingly, the global ASPR declined in individuals aged 95+ years, a phenomenon not reported previously. A possible explanation for this turning relation between ASPR and age may be that the nrCAVD and concurrent diseases with shared risk factors would lead to early death before 95 years old. The turning point of ASPR according to age came later in 2019 than that in 1990, which may be attributable to population aging and healthcare progress.

Many studies have demonstrated that hypertension is one of the causes of nrCAVD and can result in about a 20% increase in risk of nrCAVD [19,20,21]. In a prospective, observational study of 101 participants with mild or moderate aortic stenosis, Lionel Tastet et al. found that patients with systolic hypertension had much faster aortic valve calcification progression compared with those without systolic hypertension during a 2-year follow-up [22]. In addition, in a cohort study of 5.4 million UK participants, the authors reported that each 20 mmHg increment in systolic blood pressure (SBP) was associated with a 41% higher risk of aortic stenosis and a 38% higher risk of aortic regurgitation, and these associations were stronger in younger participants [23]. In the current study, we observed that DALYs of nrCAVD attributable to high SBP increased in most regions from 1990 to 2019, except for the high-SDI region. Noteworthily, the risk factor attribution of high SBP increased in individuals <50 years old, while it decreased in those ≥50 years old. These two findings are coordinated because population aging in the high-SDI region is more severe than that in developing regions. The effect of high SBP on nrCAVD in terms of age in our study is in line with what was found in the UK cohort [24]. Our findings suggest that better management of high SBP may help to reduce the burden of nrCAVD in developing countries, especially for younger individuals. Of note, the causal relationship between excessive sodium consumption and increased blood pressure has long been demonstrated. However, our study showed that diet high in sodium had little effect on the disease burden of nrCAVD. Hence, the present study indicates that sodium is not an independent risk factor for nrCAVD.

It is well established that the most profound factors promoting epidemiologic transition over the past 30 years are population growth and aging, which lead to a dramatically increased burden of non-communicable diseases [4]. It is very important to identify the contribution of each risk factor in different countries so as to reduce the burden of nrCAVD and improve health care. The present studied revealed that population growth was the main contribution to the increased burden of nrCAVD DALYs globally, and this contribution proportion was inversely related to the SDI value. By contrast, aging contributed more to the increased burden of nrCAVD in regions with higher SDI value. Our findings indicate that improvements in access to healthcare would reduce the burden of nrCAVD.

Mortality due to nrCAVD is being modified by advances in transcatheter aortic valve replacement (TAVR) over the past ten years [17,24]. A recent study based on the US National Center for Health Statistics reported that age-adjusted mortality rate (ADMR) of aortic stenosis increased before 2013 and declined after that [25]. Interestingly, we found that the global ADMR of nrCAVD also increased from 2003 to 2013 and declined after 2013. This ADMR trend of nrCAVD was especially obvious in the high-SDI region. The decrease in ADMR since 2013 occurred at a time when the number of TAVR procedures increased markedly, which suggested the reduced mortality of nrCAVD may be related to TAVR therapy.

The increased burden of non-communicable diseases was accompanied by social, economic, and medical developments [4,26]. Therefore, it is not surprising to find that the prevalence, incidence, mortality, and DALYs of nrCAVD were positively related to SDI values in our study. The prevalence and incidence of nrCAVD are much higher in the high-SDI and high-middle-SDI regions than those in other regions. Notably, the case number of nrCAVD is very large in some developing countries. For instance, China has the most cases of nrCAVD, which increased about fifty fold over the past 30 years. A similar trend might occur in India in the following years. Therefore, it is urgent for these regions to take actions to reduce the burden of nrCAVD. Using frontier analysis, our study suggests that some developed countries, such as Germany and Norway, can also reduce the burden of nrCAVD if appropriate measures are taken in the future. Considering the rapid advances are being achieved in TAVI, they may play an important role in reducing the burden of nrCAVD. It may be necessary for these regions to introduce and popularize TAVI therapy in future.

There were several limitations in the present study. First, although the DisMod-MR 2.1 could control potential biases in the data of GBD 2019, the inadequate quality and quantity of input data from some regions may have adverse effects on the accuracy of estimates. Second, different methods of diagnosis may result in differences in the incidence and prevalence of nrCAVD. The transthoracic echocardiography (TEE) is less sensitive than cardiac computed tomography in detecting nrCAVD, whereas nrCAVD is diagnosed by TEE in most cases [27,28]. Third, symptomatic and asymptomatic nrCAVD, and aortic stenosis and aortic regurgitation were mixed in the GBD 2019 database. Subtyping characteristics of nrCAVD are needed to identify the burden of nrCAVD in detail in the future.

## 5. Conclusions

The prevalence and incidence of nrCAVD increased markedly from 1990 to 2019, particularly in high-SDI and high-middle-SDI regions. Population growth was the main contributing factor to the increased burden of nrCAVD globally, while aging played the leading role in the high-SDI region. Better management of high SBP or young individuals may help to reduce burden of nrCAVD. There was a downward trend in the mortality due to nrCAVD since 2013, which is possibly owing to profound advances in transcatheter aortic valve replacement. Some countries with a high SDI value, such as Germany and Norway, may reduce burdens of nrCAVD using their socio-demographic resources.

## Figures and Tables

**Table 1:** The prevalence of cases and age-standardized prevalence rate of non-rheumatic calcific aortic valve disease in 1990 and 2019, and their temporal trends from 1990 to 2019.

	1990		2019		1990–2019	1990–2019
	Cases No. (95% UI)	ASPR (per 100,000) No. (95% UI)	Cases No. (95% UI)	ASPR (per 100,000) No. (95% UI)	ASPR Percentage Change (95% UI)	AAPC No. (95% CI)
Global	1,732,989 (1,431,469 to 2,074,809)	45.54 (37.61 to 54.67)	9,404,078 (8,079,604 to 10,889,727)	116.34 (100.39 to 134.5)	155.47 (141.66 to 171.7)	3.36 (2.77 to 3.95)
Female	838,493 (690,259 to 1,016,936)	40.28 (32.99 to 48.82)	4,376,817 (3,771,235 to 5,082,805)	99.86 (86.1 to 115.88)	147.89 (134.1 to 165.7)	3.31 (2.86 to 3.75)
Male	894,496 (741,593 to 1,067,498)	51.19 (42.68 to 60.91)	5,027,261 (4,276,877 to 5,861,586)	133.38 (113.79 to 154.58)	160.54 (146.7 to 176.16)	3.38 (2.68 to 4.08)
High SDI	1,324,934 (1,090,000 to 1,602,157)	126.83 (104.61 to 152.72)	5,095,444 (4,402,067 to 5,933,379)	273.52 (237.08 to 315.22)	115.66 (102.05 to 130.27)	2.79 (2.18 to 3.41)
High-middle SDI	364,934 (300,689 to 436,285)	33.9 (27.98 to 40.44)	3,569,820 (3,002,415 to 4,203,730)	174.53 (147.33 to 204.54)	414.83 (391.56 to 443.21)	5.89 (5.55 to 6.23)
Middle SDI	30,505 (24,085 to 37,764)	2.82 (2.23 to 3.49)	658,545 (529,926 to 800,781)	25.93 (20.92 to 31.47)	821.11 (709.87 to 944.23)	8.04 (7.89 to 8.19)
Low-middle SDI	9361 (7345 to 11,713)	1.45 (1.15 to 1.79)	67,258 (54,355 to 81,974)	4.78 (3.86 to 5.82)	229.82 (201.99 to 259.51)	4.16 (4.08 to 4.24)
Low SDI	2930 (2295 to 3719)	1.12 (0.88 to 1.38)	9675 (7605 to 12,135)	1.67 (1.34 to 2.07)	49.94 (44.14 to 56.39)	1.39 (1.34 to 1.44)
High-income Asia Pacific	469,556 (383,483 to 566,437)	233.42 (191.41 to 280.25)	1,715,700 (1,450,883 to 2,042,127)	408.4 (348.93 to 479.89)	74.96 (63.35 to 86.36)	1.94 (1.8 to 2.08)
High-income North America	687,664 (557,975 to 840,921)	191.35 (155.87 to 232.93)	1,492,891 (1,305,203 to 1,727,501)	244.39 (214.46 to 279.6)	27.72 (13.83 to 44.93)	1.86 (0.81 to 2.92)
Western Europe	218,212 (177,736 to 268,116)	37.65 (30.75 to 45.97)	1,862,787 (1,577,560 to 2,209,085)	204.84 (174.62 to 240.59)	444 (398.78 to 494.86)	6.38 (5.73 to 7.03)
Australasia	9877 (8177 to 11,826)	41.77 (34.78 to 49.88)	320,825 (272,234 to 381,057)	649.5 (552 to 772.74)	1454.86 (1236.35 to 1697.07)	9.97 (9.85 to 10.08)
Andean Latin America	831 (661 to 1026)	3.77 (3.02 to 4.63)	33,352 (28,251 to 39,016)	59.04 (50.07 to 69.05)	1465.67 (1241.46 to 1733.05)	10.1 (9.49 to 10.72)
Tropical Latin America	8034 (6435 to 9814)	7.79 (6.23 to 9.54)	58,601 (47,232 to 72,054)	23.73 (19.16 to 29.01)	204.51 (180.25 to 234)	3.81 (3.7 to 3.92)
Central Latin America	6996 (5589 to 8531)	7.98 (6.44 to 9.71)	76,957 (63,860 to 91,384)	31.82 (26.45 to 37.75)	298.86 (261.69 to 344.2)	5.06 (4.14 to 6)
Southern Latin America	5497 (4391 to 6936)	11.87 (9.53 to 14.91)	101,893 (87,124 to 120,797)	122.72 (104.78 to 145.67)	933.61 (766.84 to 1118.16)	8.22 (8.07 to 8.38)
Caribbean	3359 (2751 to 4009)	12.61 (10.3 to 15.1)	45,467 (37,694 to 54,849)	87.51 (72.62 to 105.56)	594.1 (504.42 to 696.1)	7.07 (6.73 to 7.41)
Central Europe	154,665 (128,343 to 184,574)	104.18 (86.62 to 123.47)	1,260,558 (1,067,648 to 1,479,616)	608.31 (517.94 to 713.51)	483.91 (434.98 to 537.45)	6.63 (6.24 to 7.02)
Eastern Europe	121,662 (97,120 to 148,877)	43.32 (34.79 to 52.76)	1,328,687 (1,065,696 to 1,605,206)	395.8 (319.64 to 477)	813.6 (752.33 to 886.02)	8.12 (7.89 to 8.34)
Central Asia	3767 (2956 to 4713)	7.92 (6.22 to 9.83)	41,060 (33,495 to 48,810)	53.22 (43.89 to 62.92)	572.13 (489.66 to 669.91)	6.82 (6.7 to 6.93)
North Africa and Middle East	9696 (7638 to 11,898)	4.92 (3.9 to 6.04)	54,300 (43,226 to 66,785)	10.83 (8.67 to 13.35)	120.05 (104.74 to 135.33)	3.01 (2.55 to 3.47)
South Asia	8332 (6398 to 10,638)	1.34 (1.04 to 1.67)	30,188 (23,678 to 37,633)	2.03 (1.6 to 2.52)	51.55 (45.4 to 58.54)	1.44 (1.4 to 1.48)
Southeast Asia	1785 (1337 to 2357)	0.7 (0.53 to 0.91)	23,986 (18,739 to 30,273)	4.06 (3.21 to 5.09)	480.45 (408.41 to 566.35)	6.17 (6.05 to 6.29)
East Asia	18,272 (13,682 to 24,325)	2.1 (1.57 to 2.74)	891,018 (707,255 to 1,093,313)	42.41 (33.87 to 51.68)	1920.64 (1545.86 to 2360.13)	11.07 (10.58 to 11.57)
Oceania	110 (89 to 136)	4.3 (3.49 to 5.36)	1130 (904 to 1374)	18.61 (14.95 to 22.75)	332.86 (271.32 to 396.31)	4.7 (4.17 to 5.24)
Western Sub-Saharan Africa	1300 (1004 to 1627)	1.27 (0.99 to 1.58)	3870 (3002 to 4876)	1.65 (1.3 to 2.04)	29.76 (25.16 to 34.98)	0.85 (0.76 to 0.94)
Eastern Sub-Saharan Africa	763 (589 to 963)	0.93 (0.73 to 1.15)	2602 (2004 to 3271)	1.4 (1.1 to 1.74)	51.64 (43.67 to 61.1)	1.47 (1.41 to 1.53)
Central Sub-Saharan Africa	245 (189 to 315)	1 (0.79 to 1.26)	796 (621 to 1012)	1.36 (1.08 to 1.69)	36.9 (28.71 to 46.73)	1.12 (1.06 to 1.18)
Southern Sub-Saharan Africa	2365 (1845 to 2936)	8.15 (6.36 to 10.13)	57409 (44,278 to 73,386)	94.86 (72.97 to 120.7)	1064.03 (877.48 to 1280.35)	9.02 (8.62 to 9.41)

AAPC, average annual percentage change; ASPR, age-standardized prevalence rate; SDI, socio-demographic index; UI, uncertainty interval; CI, confidence interval.

## Data Availability

Data are available in an open access repository. We retrieved data from the Global Health Data Exchange query tool (https://vizhub.healthdata.org/gbd-results/), an online tool of the Global Burden of Diseases, Injuries, and Risk Factors Study 2019. The database is hosted by the Institute for Health Metrics and Evaluation at the Washington University.

## Supplementary Materials

The following supporting information can be downloaded at: https://www.mdpi.com/article/10.3390/jcm11226733/s1, Figure S1: The age-standardized prevalence rate, incidence rate, mortality and disability-adjusted life-years of nrCAVD versus SDI quintile for men and women, 1990–2019. nrCAVD, non-rheumatic calcific aortic valve disease; SDI, socio-demographic index; Figure S2: The ASPR of nrCAVD versus SDI quintile for men and women by region, 1990–2019. The black line, a LOWESS smoother, shows the expected value only on the SDI values of the global regions between 1990 and 2019. ASPR, age-standardized prevalence rate; LOWESS, locally weighted scatterplot smoothing; nrCAVD, non-rheumatic calcific aortic valve disease; SDI, socio-demographic index; Figure S3. Percentage contributions of major risk factors attributed for DALYs of nrCAVD stratified by age and sex. DALYs, disability-adjusted life-years; nrCAVD, non-rheumatic calcific aortic valve disease; Figure S4: Percentage contributions of major risk factors attributed for DALYs of nrCAVD stratified by SDI quintile and age group. DALYs, disability-adjusted life-years; nrCAVD, non-rheumatic calcific aortic valve disease; SDI, socio-demographic index; Table S1: The prevalence cases and age-standardised prevalence rate of non-rheumatic calcific aortic valve disease in 1990 and 2019, and their temporal trends from 1990 to 2019 in 204 countries and territories; Table S2: The incidence cases and age-standardized incidence rate of non-rheumatic calcific aortic valve disease in 1990 and 2019 and its temporal trends from 1990 to 2019; Table S3: The DALYs and age-standardized DALYs rate of non-rheumatic calcific aortic valve disease in 1990 and 2019 and its temporal trends from 1990 to 2019; Table S4: The death cases and age-standardized mortality rate of cardiomyopathy between 1990 and 2019 and its temporal trends from 1990 to 2019; Table S5: Changes in DALYs number according to population-level determinants from 1990 to 2019; Table S6: Frontier DALYs and effective difference by country or territory [29].

## References

[1] Vahanian A., Beyersdorf F., Praz F., Milojevic M., Baldus S., Bauersachs J., Capodanno D., Conradi L., De Bonis M., De Paulis R. (2022). 2021 ESC/EACTS Guidelines for the management of valvular heart disease. Eur. Heart J..

[2] Carbone R.G. (2022). Advancements in calcific aortic valve disease. Int. J. Cardiol..

[3] Buttner P., Feistner L., Lurz P., Thiele H., Hutcheson J.D., Schlotter F. (2021). Dissecting Calcific Aortic Valve Disease-The Role, Etiology, and Drivers of Valvular Fibrosis. Front. Cardiovasc. Med..

[4] Vos T., Lim S.S., Abbafati C., Abbas K.M., Abbasi M., Abbasifard M., Abbasi-Kangevari M., Abbastabar H., Abd-Allah F., Bhutta Z.A. (2020). Global burden of 369 diseases and injuries in 204 countries and territories, 1990–2019: A systematic analysis for the Global Burden of Disease Study 2019. Lancet.

[5] Arabloo J., Omidi N., Rezapour A., Sarabi A.A., Mojtaba G.S., Azari S. (2022). The burden of nonrheumatic valvular heart diseases in Iran between 1990 and 2017: Results from the global burden of disease study 2017. Int. J. Cardiol. Heart Vasc..

[6] Nejad M., Ahmadi N., Mohammadi E., Shabani M., Sherafati A., Aryannejad A., Rezaei N., Ghanbari A., Yoosefi M., Aminorroaya A. (2022). Global and regional burden and quality of care of non-rheumatic valvular heart diseases: A systematic analysis of Global Burden of Disease 1990–2017. Int. J. Qual. Health Care.

[7] Williams M.C., Massera D., Moss A.J., Bing R., Bularga A., Adamson P.D., Hunter A., Alam S., Shah A., Pawade T. (2021). Prevalence and clinical implications of valvular calcification on coronary computed tomography angiography. Eur. Heart J. Cardiovasc. Imaging.

[8] Coffey S., Cox B., Williams M.J. (2014). The prevalence, incidence, progression, and risks of aortic valve sclerosis: A systematic review and meta-analysis. J. Am. Coll. Cardiol..

[9] Yadgir S., Johnson C.O., Aboyans V., Adebayo O.M., Adedoyin R.A., Afarideh M., Alahdab F., Alashi A., Alipour V., Arabloo J. (2020). Global, Regional, and National Burden of Calcific Aortic Valve and Degenerative Mitral Valve Diseases, 1990–2017. Circulation.

[10] Das G.P. (1994). Standardization and decomposition of rates from cross-classified data. Genus.

[11] Chevan A., Sutherland M. (2009). Revisiting Das Gupta: Refinement and extension of standardization and decomposition. Demography.

[12] Xie Y., Bowe B., Xian H., Balasubramanian S., Al-Aly Z. (2015). Rate of Kidney Function Decline and Risk of Hospitalizations in Stage 3A CKD. Clin. J. Am. Soc. Nephrol..

[13] Barber R.M., Fullman N., Sorensen R.J., Bollyky T., McKee M., Nolte E., Abajobir A.A., Avate K.H., Abbafati C., Davey G. (2017). Healthcare Access and Quality Index based on mortality from causes amenable to personal health care in 195 countries and territories, 1990–2015: A novel analysis from the Global Burden of Disease Study 2015. Lancet.

[14] Ferreira-Gonzalez I., Pinar-Sopena J., Ribera A., Marsal J.R., Cascant P., Gonzalez-Alujas T., Evangelista A., Brotons C., Moral I., Permanyer-Miralda G. (2013). Prevalence of calcific aortic valve disease in the elderly and associated risk factors: A population-based study in a Mediterranean area. Eur. J. Prev. Cardiol..

[15] Lindman B.R., Sukul D., Dweck M.R., Madhavan M.V., Arsenault B.J., Coylewright M., Merryman W.D., Newby D.E., Lewis J., Harrell F.J. (2021). Evaluating Medical Therapy for Calcific Aortic Stenosis: JACC State-of-the-Art Review. J. Am. Coll. Cardiol..

[16] Kostyunin A.E., Yuzhalin A.E., Ovcharenko E.A., Kutikhin A.G. (2019). Development of calcific aortic valve disease: Do we know enough for new clinical trials?. J. Mol. Cell. Cardiol..

[17] Kraler S., Blaser M.C., Aikawa E., Camici G.G., Luscher T.F. (2022). Calcific aortic valve disease: From molecular and cellular mechanisms to medical therapy. Eur. Heart J..

[18] Ye T., Ma T., Wang Q., Zhang C.M., Cao L., Xu B.D., Zong G.J. (2019). Prevalence and risk factors of aortic valve calcification among the elderly residents of Wuxi city, Jiangsu province. Zhonghua Xin Xue Guan Bing Za Zhi.

[19] Stewart B.F., Siscovick D., Lind B.K., Gardin J.M., Gottdiener J.S., Smith V.E., Kitzman D.W., Otto C.M. (1997). Clinical factors associated with calcific aortic valve disease. Cardiovascular Health Study. J. Am. Coll. Cardiol..

[20] Lindroos M., Kupari M., Valvanne J., Strandberg T., Heikkila J., Tilvis R. (1994). Factors associated with calcific aortic valve degeneration in the elderly. Eur. Heart J..

[21] Chen H.Y., Engert J.C., Thanassoulis G. (2019). Risk factors for valvular calcification. Curr. Opin. Endocrinol. Diabetes Obes..

[22] Tastet L., Capoulade R., Clavel M.A., Larose E., Shen M., Dahou A., Arsenault M., Mathieu P., Bedard E., Dumesnil J.G. (2017). Systolic hypertension and progression of aortic valve calcification in patients with aortic stenosis: Results from the PROGRESSA study. Eur. Heart J. Cardiovasc. Imaging.

[23] Rahimi K., Mohseni H., Kiran A., Tran J., Nazarzadeh M., Rahimian F., Woodward M., Dwyer T., MacMahon S., Otto C.M. (2018). Elevated blood pressure and risk of aortic valve disease: A cohort analysis of 5.4 million UK adults. Eur. Heart J..

[24] Osnabrugge R.L., Mylotte D., Head S.J., Van Mieghem N.M., Nkomo V.T., LeReun C.M., Bogers A.J., Piazza N., Kappetein A.P. (2013). Aortic stenosis in the elderly: Disease prevalence and number of candidates for transcatheter aortic valve replacement: A meta-analysis and modeling study. J. Am. Coll. Cardiol..

[25] Bevan G.H., Zidar D.A., Josephson R.A., Al-Kindi S.G. (2019). Mortality Due to Aortic Stenosis in the United States, 2008–2017. JAMA.

[26] Murray C.J., Aravkin A.Y., Zheng P., Abbafati C., Abbas K.M., Abbasi-Kangevari M., Abd-Allah F., Abdelalim A., Abdollahi M., Borzouei S. (2020). Global burden of 87 risk factors in 204 countries and territories, 1990–2019: A systematic analysis for the Global Burden of Disease Study 2019. Lancet.

[27] Walsh C.R., Larson M.G., Kupka M.J., Levy D., Vasan R.S., Benjamin E.J., Manning W.J., Clouse M.E., O’Donnell C.J. (2004). Association of aortic valve calcium detected by electron beam computed tomography with echocardiographic aortic valve disease and with calcium deposits in the coronary arteries and thoracic aorta. Am. J. Cardiol..

[28] Shah R.G., Novaro G.M., Blandon R.J., Whiteman M.S., Asher C.R., Kirsch J. (2009). Aortic valve area: Meta-analysis of diagnostic performance of multi-detector computed tomography for aortic valve area measurements as compared to transthoracic echocardiography. Int. J. Cardiovasc. Imaging.

[29] Hwang S.-N., Lee H.-S., Zhu J. (2016). Handbook of Operations Analytics Using Data Envelopment Analysis.

