# Supplementary material for: Global Burden and Improvement Gap of Non-Rheumatic Calcific Aortic Valve Disease: 1990–2019 Findings from Global Burden of Disease Study 2019
Source: J Clin Med. 2022 Nov 14;11(22):6733. doi: 10.3390/jcm11226733 (PMC9698619; doi:10.3390/jcm11226733)
Supplement: Supplementary file 1 [file jcm-11-06733-s001.zip › Supplemental Table S1 nrCAVD prevalence in 204 countries.pdf]

Table S1. The prevalence cases and age-standardised prevalence rate of non-rheumatic calcific aortic valve disease in 1990 and 2019, and their temporal trends from 1990 to 2019 in 204 countries and territories.

	1990		2019		1990–2019	1990–2019
	Cases No. (95% UI)	ASPR (per 100,000) No. (95% UI)	Cases No. (95% UI)	ASPR (per 100,000) No. (95% UI)	ASPR Percentage change (95% UI)	AAPC No. (95% CI)
Afghanistan	61 (46 to 79)	0.78 (0.6 to 1.02)	170 (125 to 228)	1.04 (0.8 to 1.34)	32.63 (20.02 to 49.67)	0.97 (0.91 to 1.04)
Albania	654 (543 to 782)	31.31 (26.06 to 37.57)	10142 (8377 to 12306)	239.18 (199.2 to 289.35)	663.82 (555.89 to 780.35)	7.25 (7.12 to 7.39)
Algeria	403 (311 to 503)	2.92 (2.28 to 3.66)	1729 (1337 to 2185)	4.64 (3.63 to 5.84)	58.97 (41.87 to 81.11)	1.59 (1.5 to 1.69)
American Samoa	9 (7 to 11)	45.2 (35.49 to 57.11)	91 (72 to 114)	195.91 (153.9 to 245.53)	333.44 (252.1 to 439.83)	4.26 (3.25 to 5.29)
Andorra	4 (3 to 5)	7.18 (5.66 to 9.14)	237 (180 to 295)	171.77 (130.03 to 213.02)	2291.79 (1718.67 to 2916.39)	11.83 (10.38 to 13.3)
Angola	40 (30 to 51)	0.91 (0.72 to 1.13)	149 (115 to 190)	1.2 (0.95 to 1.5)	31.79 (19.72 to 44.76)	0.97 (0.94 to 1)
Antigua and Barbuda	3 (3 to 4)	6.4 (5.23 to 7.74)	31 (26 to 37)	29.65 (24.38 to 35.08)	363 (299.79 to 441.26)	5.33 (5.21 to 5.46)
Argentina	3539 (2751 to 4664)	10.96 (8.53 to 14.45)	55367 (47036 to 66507)	102.43 (86.97 to 122.81)	834.79 (638.81 to 1056.96)	7.75 (7.54 to 7.96)
Armenia	142 (108 to 182)	5.05 (3.89 to 6.4)	2288 (1859 to 2807)	55.18 (44.9 to 67.49)	993.28 (787.84 to 1225.04)	8.78 (8.29 to 9.27)
Australia	6565 (5425 to 7893)	33.21 (27.6 to 39.89)	288497 (243985 to 345487)	695.19 (590.19 to 832.23)	1993.12 (1667.94 to 2369.86)	11.05 (10.9 to 11.19)
Austria	6099 (5184 to 7207)	52.03 (44.12 to 61.23)	121170 (104958 to 137387)	668.14 (576.67 to 759.72)	1184.11 (1012.09 to 1417.75)	9.57 (9.19 to 9.96)
Azerbaijan	249 (186 to 328)	4.86 (3.63 to 6.28)	4647 (3661 to 5739)	45.27 (36.12 to 55.52)	831.6 (651.93 to 1060.38)	8.15 (7.75 to 8.55)
Bahamas	14	8.71	156	38.24	338.99	5.41

	(12 to 17)	(7.08 to 10.42)	(128 to 187)	(31.29 to 45.62)	(286.24 to 399.16)	(5.12 to 5.71)
Bahrain	52<br>(39 to 66)	19.08<br>(14.93 to 23.99)	603<br>(457 to 768)	40.24<br>(31.18 to 50.8)	110.95<br>(90.65 to 134.2)	2.82<br>(2.56 to 3.09)
Bangladesh	565<br>(434 to 731)	1.09<br>(0.85 to 1.4)	2240<br>(1745 to 2815)	1.64<br>(1.28 to 2.04)	49.93<br>(34.71 to 68.32)	1.32<br>(1.19 to 1.45)
Barbados	35<br>(29 to 41)	12.59<br>(10.35 to 15)	276<br>(222 to 332)	57.18<br>(46.41 to 68.28)	354.31<br>(293.14 to 426.36)	5.3<br>(4.99 to 5.61)
Belarus	3026<br>(2368 to 3726)	23.35<br>(18.4 to 28.59)	35854<br>(28628 to 44269)	229.73<br>(183.94 to 280.39)	883.76<br>(760.78 to 1033.67)	8.36<br>(8.12 to 8.6)
Belgium	517<br>(407 to 666)	3.35<br>(2.62 to 4.33)	8836<br>(6944 to 11221)	39<br>(30.82 to 48.99)	1063.62<br>(860.09 to 1358.13)	8.62<br>(7.58 to 9.67)
Belize	5<br>(4 to 6)	5.17<br>(4.13 to 6.34)	125<br>(100 to 160)	42.7<br>(33.9 to 55.26)	725.3<br>(583.1 to 959.67)	7.59<br>(6.56 to 8.63)
Benin	24<br>(19 to 31)	1.08<br>(0.85 to 1.34)	99<br>(76 to 128)	1.65<br>(1.27 to 2.08)	52.75<br>(36.47 to 72.02)	1.32<br>(1.14 to 1.51)
Bermuda	34<br>(27 to 42)	53.42<br>(42.76 to 65.48)	347<br>(291 to 414)	274.8<br>(231.59 to 328.97)	414.37<br>(324.24 to 531.07)	5.75<br>(5.67 to 5.83)
Bhutan	4<br>(3 to 6)	1.58<br>(1.22 to 2.03)	30<br>(22 to 40)	4.95<br>(3.67 to 6.65)	213.01<br>(158.37 to 300.94)	3.93<br>(3.53 to 4.32)
Bolivia (Plurinational State of)	87<br>(70 to 108)	2.49<br>(2 to 3.06)	1442<br>(1184 to 1735)	15.7<br>(12.98 to 19.01)	531.34<br>(443.09 to 637.31)	6.6<br>(6.47 to 6.73)
Bosnia and Herzegovina	1613<br>(1300 to 1965)	40.23<br>(32.33 to 48.85)	17469<br>(14381 to 21150)	294.68<br>(245.88 to 354.85)	632.4<br>(528.55 to 761.53)	7.21<br>(6.53 to 7.89)
Botswana	10<br>(8 to 12)	1.58<br>(1.26 to 1.96)	90<br>(68 to 114)	5.98<br>(4.64 to 7.49)	278.23<br>(220.85 to 345.74)	4.66<br>(4.39 to 4.93)
Brazil	7905<br>(6333 to 9659)	7.85<br>(6.26 to 9.62)	57153<br>(45927 to 70348)	23.7<br>(19.11 to 29)	201.79<br>(177.49 to 231.77)	3.77<br>(3.66 to 3.88)
Brunei Darussalam	67<br>(53 to 83)	62.64<br>(48.9 to 79.56)	899<br>(748 to 1089)	289.41<br>(246.36 to 342.68)	362.04<br>(298.27 to 458.24)	5.47<br>(5.3 to 5.65)
Bulgaria	15626	124.24	48314	357.88	188.06	3.52

	(12391 to 19290)	(98.96 to 152.51)	(39017 to 58508)	(289.22 to 429.53)	(154.58 to 222.87)	(3.2 to 3.84)
Burkina Faso	44<br>(35 to 56)	0.89<br>(0.7 to 1.13)	154<br>(119 to 196)	1.39<br>(1.08 to 1.74)	56.08<br>(40.34 to 73.85)	1.56<br>(1.44 to 1.68)
Burundi	21<br>(17 to 27)	0.83<br>(0.67 to 1.03)	41<br>(31 to 53)	0.78<br>(0.61 to 0.98)	-6.59<br>(-17.33 to 3.67)	-0.26<br>(-0.32 to -0.21)
Cabo Verde	4<br>(3 to 5)	1.74<br>(1.36 to 2.13)	16<br>(13 to 20)	3.42<br>(2.71 to 4.24)	97.17<br>(75.91 to 124.14)	2.31<br>(2.16 to 2.46)
Cambodia	17<br>(12 to 23)	0.37<br>(0.28 to 0.5)	137<br>(100 to 188)	1.15<br>(0.85 to 1.53)	207.91<br>(162.2 to 265.2)	3.91<br>(3.78 to 4.04)
Cameroon	111<br>(84 to 141)	2.13<br>(1.64 to 2.72)	451<br>(344 to 577)	3.05<br>(2.37 to 3.86)	43.12<br>(27.03 to 62.74)	1.14<br>(0.8 to 1.48)
Canada	1909<br>(1506 to 2399)	5.86<br>(4.64 to 7.34)	67765<br>(56040 to 80666)	100.8<br>(83.69 to 119.91)	1620.24<br>(1304.74 to 1985.2)	10.3<br>(9.96 to 10.64)
Central African Republic	10<br>(7 to 12)	0.76<br>(0.6 to 0.94)	19<br>(15 to 24)	0.82<br>(0.65 to 1.01)	7.36<br>(-0.62 to 16.42)	0.26<br>(0.21 to 0.31)
Chad	24<br>(19 to 31)	0.78<br>(0.61 to 0.99)	70<br>(54 to 90)	0.99<br>(0.78 to 1.26)	26.78<br>(14.87 to 39.22)	0.8<br>(0.58 to 1.02)
Chile	1464<br>(1209 to 1765)	14.25<br>(11.79 to 17.1)	39521<br>(32889 to 47405)	166.28<br>(138.23 to 198.59)	1066.52<br>(906.49 to 1261.98)	8.86<br>(8.8 to 8.93)
China	17791<br>(13302 to 23691)	2.12<br>(1.58 to 2.76)	867917<br>(687948 to 1064921)	42.75<br>(34.03 to 52.12)	1920.39<br>(1543.48 to 2357.5)	11.07<br>(10.55 to 11.58)
Colombia	1305<br>(1010 to 1603)	7.02<br>(5.43 to 8.66)	14539<br>(11904 to 17576)	27.56<br>(22.58 to 33.42)	292.44<br>(228.03 to 380.92)	4.73<br>(4.62 to 4.85)
Comoros	3<br>(2 to 4)	1.3<br>(1.02 to 1.61)	8<br>(6 to 10)	1.47<br>(1.14 to 1.85)	13.17<br>(1.17 to 26.46)	0.41<br>(0.35 to 0.48)
Congo	16<br>(13 to 20)	1.42<br>(1.13 to 1.77)	49<br>(38 to 63)	1.77<br>(1.37 to 2.22)	24.58<br>(12.51 to 37.7)	0.78<br>(0.72 to 0.83)
Cook Islands	1<br>(1 to 2)	10.9<br>(8.57 to 13.69)	30<br>(23 to 37)	118.44<br>(91.5 to 146.01)	986.63<br>(791.49 to 1233.8)	7.97<br>(7.28 to 8.66)
Costa Rica	193	10.84	2542	48.79	350.15	5.12

	(160 to 233)	(8.98 to 13.04)	(2115 to 3037)	(40.59 to 58.17)	(286.14 to 426.13)	(4.96 to 5.29)
Croatia	4421<br>(3570 to 5316)	68.76<br>(55.68 to 82.56)	63554<br>(54339 to 73922)	761.43<br>(654.39 to 881.08)	1007.39<br>(872.45 to 1174.88)	8.78<br>(8.55 to 9)
Cuba	774<br>(638 to 929)	7.47<br>(6.16 to 8.98)	8982<br>(7468 to 10722)	49.03<br>(40.91 to 58.48)	556.26<br>(459.41 to 662.92)	6.69<br>(6.47 to 6.91)
Cyprus	42<br>(33 to 53)	4.93<br>(3.85 to 6.17)	2657<br>(2136 to 3279)	131.73<br>(106.46 to 162.28)	2574.23<br>(2097.93 to 3186.27)	13.41<br>(11.68 to 15.17)
Czechia	8928<br>(7022 to 11254)	65.24<br>(51.59 to 81.37)	135356<br>(112016 to 163024)	676.09<br>(559.92 to 802)	936.35<br>(761.57 to 1154.17)	8.26<br>(7.75 to 8.77)
Cote D'lvoire	75<br>(57 to 96)	1.37<br>(1.06 to 1.71)	243<br>(188 to 309)	1.72<br>(1.35 to 2.16)	26.26<br>(12.5 to 41.56)	0.65<br>(0.5 to 0.8)
Democratic People's Republic of Korea	144<br>(106 to 193)	0.91<br>(0.69 to 1.19)	1190<br>(916 to 1531)	3.66<br>(2.84 to 4.66)	303.76<br>(227.44 to 397.89)	4.68<br>(4.42 to 4.94)
Democratic Republic of the Congo	168<br>(130 to 217)	0.97<br>(0.77 to 1.24)	445<br>(342 to 576)	1.12<br>(0.88 to 1.41)	15.62<br>(5.39 to 26.37)	0.52<br>(0.49 to 0.55)
Denmark	395<br>(304 to 511)	4.83<br>(3.71 to 6.18)	16873<br>(13726 to 20994)	142.9<br>(116.33 to 175.93)	2859.7<br>(2329.24 to 3473.71)	12.82<br>(9.8 to 15.91)
Djibouti	2<br>(2 to 3)	1.22<br>(0.97 to 1.52)	11<br>(8 to 14)	1.55<br>(1.22 to 1.94)	27.33<br>(15.34 to 42.52)	0.86<br>(0.8 to 0.91)
Dominica	5<br>(4 to 6)	7.06<br>(5.81 to 8.49)	33<br>(27 to 40)	37.31<br>(30.66 to 44.91)	428.72<br>(354.87 to 522.82)	5.85<br>(5.66 to 6.03)
Dominican Republic	137<br>(110 to 166)	3.35<br>(2.69 to 4.14)	2206<br>(1824 to 2650)	23.21<br>(19.2 to 27.72)	592.05<br>(489.82 to 717.39)	6.72<br>(6.4 to 7.04)
Ecuador	366<br>(292 to 454)	6.48<br>(5.17 to 8)	20348<br>(17505 to 23404)	132.9<br>(114.58 to 153.07)	1951.96<br>(1622.32 to 2364.32)	10.73<br>(9.59 to 11.89)
Egypt	2376<br>(1758 to 3087)	7.14<br>(5.3 to 9.32)	9706<br>(7369 to 12610)	13.43<br>(10.24 to 17.4)	88.04<br>(67.41 to 114.55)	2.68<br>(1.46 to 3.91)
El Salvador	91<br>(71 to 114)	2.98<br>(2.31 to 3.76)	843<br>(651 to 1101)	14.48<br>(11.13 to 18.99)	385.96<br>(301.83 to 507.68)	5.78<br>(5.55 to 6.01)
Equatorial Guinea	2	0.84	21	4.01	379.58	5.95

	(1 to 2)	(0.66 to 1.06)	(16 to 27)	(3.01 to 5.09)	(308.17 to 464.01)	(5.49 to 6.42)
Eritrea	9<br>(7 to 11)	0.75<br>(0.6 to 0.91)	27<br>(20 to 34)	0.86<br>(0.68 to 1.07)	15.16<br>(3.42 to 27.19)	0.48<br>(0.42 to 0.54)
Estonia	1052<br>(854 to 1274)	51.27<br>(41.94 to 61.85)	18715<br>(14075 to 23512)	759.94<br>(578.58 to 943.34)	1382.22<br>(1126.44 to 1701.79)	9.75<br>(9.11 to 10.39)
Eswatini	11<br>(8 to 16)	3.66<br>(2.59 to 5.11)	66<br>(45 to 89)	10.39<br>(7.14 to 13.98)	183.83<br>(128.16 to 282.55)	3.96<br>(2.83 to 5.1)
Ethiopia	156<br>(118 to 203)	0.68<br>(0.52 to 0.86)	512<br>(387 to 657)	1.05<br>(0.81 to 1.35)	55.3<br>(43.3 to 69.95)	1.62<br>(1.49 to 1.74)
Fiji	12<br>(10 to 16)	3.96<br>(3.09 to 5.19)	158<br>(124 to 199)	22.15<br>(17.46 to 27.72)	459.95<br>(353.4 to 571.72)	5.72<br>(5.13 to 6.31)
Finland	3009<br>(2471 to 3645)	40.11<br>(33.2 to 48.45)	53982<br>(40850 to 67092)	432.08<br>(330.68 to 536.75)	977.36<br>(743.83 to 1281.25)	9.1<br>(8.56 to 9.64)
France	4797<br>(3789 to 6019)	5.8<br>(4.6 to 7.32)	109065<br>(86214 to 138503)	79.49<br>(63.32 to 99.16)	1270.52<br>(1026.92 to 1564.48)	9.85<br>(8.8 to 10.91)
Gabon	11<br>(9 to 14)	1.9<br>(1.49 to 2.38)	112<br>(84 to 147)	10.01<br>(7.57 to 12.9)	427.46<br>(339.19 to 539.01)	5.97<br>(5.41 to 6.53)
Gambia	5<br>(4 to 7)	1.21<br>(0.96 to 1.54)	18<br>(14 to 23)	1.57<br>(1.23 to 1.96)	29.83<br>(15.94 to 45.73)	0.82<br>(0.73 to 0.91)
Georgia	1122<br>(877 to 1374)	17.93<br>(14.11 to 21.82)	5384<br>(4501 to 6432)	92.12<br>(76.88 to 110.29)	413.8<br>(343.38 to 512.41)	6<br>(5.51 to 6.48)
Germany	7099<br>(5581 to 8885)	5.71<br>(4.5 to 7.16)	287039<br>(235176 to 348617)	147.9<br>(121.44 to 179.61)	2492.23<br>(2025.32 to 3028.8)	13.43<br>(11.67 to 15.21)
Ghana	73<br>(55 to 95)	0.95<br>(0.73 to 1.22)	366<br>(282 to 463)	1.89<br>(1.47 to 2.33)	98.99<br>(76.56 to 129.32)	2.35<br>(2.23 to 2.47)
Greece	1002<br>(779 to 1296)	6.5<br>(5.1 to 8.34)	30220<br>(24352 to 37184)	126.19<br>(103.74 to 154.94)	1840.26<br>(1476.99 to 2285.57)	11.11<br>(9.47 to 12.79)
Greenland	1<br>(1 to 2)	3.79<br>(3.02 to 4.71)	30<br>(24 to 36)	45.34<br>(37.49 to 54.11)	1096.89<br>(879.69 to 1328.23)	8.63<br>(8.27 to 8.99)
Grenada	3	4.7	26	22.06	369.06	5.08

	(2 to 4)	(3.45 to 5.82)	(20 to 32)	(17.49 to 27.16)	(277.75 to 508.36)	(4.66 to 5.51)
Guam	18<br>(14 to 22)	26.5<br>(21.68 to 32.57)	360<br>(282 to 446)	189.76<br>(149.17 to 234.59)	616.09<br>(486.01 to 767.87)	7.08<br>(6.51 to 7.64)
Guatemala	80<br>(61 to 103)	1.96<br>(1.49 to 2.5)	761<br>(589 to 938)	6.42<br>(5.02 to 7.93)	227.99<br>(183.45 to 285.27)	4.21<br>(4.15 to 4.28)
Guinea	31<br>(24 to 41)	0.87<br>(0.68 to 1.12)	81<br>(63 to 103)	1.25<br>(0.99 to 1.57)	43.28<br>(27.55 to 61.22)	1.22<br>(1.09 to 1.35)
Guinea-Bissau	5<br>(4 to 6)	1.04<br>(0.8 to 1.31)	11<br>(9 to 14)	1.16<br>(0.92 to 1.45)	11.84<br>(0.82 to 26.71)	0.29<br>(0.07 to 0.51)
Guyana	22<br>(17 to 27)	5.08<br>(4.06 to 6.25)	195<br>(156 to 238)	29.2<br>(23.48 to 35.73)	474.63<br>(378.27 to 608.82)	6.18<br>(5.89 to 6.48)
Haiti	89<br>(69 to 109)	2.51<br>(1.95 to 3.08)	552<br>(418 to 701)	7.17<br>(5.36 to 9.18)	185.61<br>(134.07 to 235.36)	3.57<br>(3.34 to 3.79)
Honduras	60<br>(47 to 75)	2.73<br>(2.14 to 3.4)	570<br>(459 to 697)	8.74<br>(7.08 to 10.67)	219.49<br>(179.58 to 268.24)	3.98<br>(3.83 to 4.14)
Hungary	27752<br>(23433 to 32723)	186.85<br>(157.65 to 219.63)	184447<br>(159114 to 218515)	997.07<br>(861.74 to 1167.68)	433.61<br>(368.27 to 512.56)	5.86<br>(5.64 to 6.09)
Iceland	18<br>(14 to 23)	6.38<br>(5.01 to 8.12)	861<br>(704 to 1044)	155.92<br>(128.08 to 189.36)	2345.02<br>(1906.73 to 2907.6)	12.51<br>(10.84 to 14.21)
India	6162<br>(4676 to 7873)	1.21<br>(0.93 to 1.51)	23315<br>(18286 to 29292)	1.94<br>(1.52 to 2.42)	60.28<br>(53.5 to 67.71)	1.67<br>(1.64 to 1.7)
Indonesia	558<br>(405 to 748)	0.55<br>(0.41 to 0.73)	6015<br>(4543 to 7787)	2.72<br>(2.09 to 3.5)	390.15<br>(331.77 to 457.71)	5.58<br>(5.46 to 5.7)
Iran (Islamic Republic of)	1124<br>(872 to 1428)	3.54<br>(2.74 to 4.47)	3464<br>(2697 to 4337)	4.18<br>(3.26 to 5.23)	18.05<br>(10.59 to 26.31)	0.48<br>(0.21 to 0.74)
Iraq	684<br>(508 to 896)	8.13<br>(6.06 to 10.68)	1492<br>(1114 to 1949)	5.67<br>(4.3 to 7.39)	-30.26<br>(-39.27 to -21.2)	-1.37<br>(-2.16 to -0.56)
Ireland	263<br>(204 to 340)	6.3<br>(4.89 to 8.06)	14640<br>(11042 to 17825)	192.79<br>(145.96 to 234.79)	2960.65<br>(2222.19 to 3809.19)	13.27<br>(11.8 to 14.75)
Israel	191	3.87	4366	37.58	871.22	8.63

	(145 to 246)	(2.95 to 4.91)	(3423 to 5539)	(29.51 to 47.6)	(677.4 to 1103.09)	(7.79 to 9.47)
Italy	74111<br>(60295 to 89699)	84.07<br>(68.6 to 101.04)	560971<br>(474688 to 662132)	408.26<br>(347.04 to 476.32)	385.61<br>(346.8 to 434.6)	5.56<br>(4.32 to 6.81)
Jamaica	68<br>(54 to 84)	3.89<br>(3.07 to 4.86)	604<br>(466 to 803)	20.35<br>(15.71 to 27.14)	423<br>(329.23 to 553.76)	5.66<br>(5.2 to 6.11)
Japan	439043<br>(357529 to 530647)	261.58<br>(214.61 to 314.48)	1411874<br>(1186418 to 1700725)	435.47<br>(369.13 to 514.16)	66.48<br>(55.42 to 77.26)	1.77<br>(1.68 to 1.85)
Jordan	107<br>(79 to 145)	6.34<br>(4.74 to 8.73)	1510<br>(1150 to 1917)	18.74<br>(14.28 to 23.93)	195.41<br>(140.1 to 261.76)	3.93<br>(3.65 to 4.2)
Kazakhstan	1535<br>(1176 to 1952)	11.96<br>(9.2 to 15.25)	17880<br>(14238 to 21613)	97.94<br>(77.96 to 117.06)	718.91<br>(588.22 to 889.22)	7.43<br>(7.1 to 7.75)
Kenya	120<br>(92 to 153)	1.32<br>(1.03 to 1.7)	709<br>(537 to 898)	2.77<br>(2.12 to 3.52)	108.94<br>(86.94 to 134.8)	2.64<br>(2.5 to 2.78)
Kiribati	1<br>(1 to 1)	2.08<br>(1.64 to 2.71)	4<br>(3 to 5)	6.5<br>(4.98 to 8.33)	212.75<br>(166.94 to 267.1)	3.67<br>(3.03 to 4.3)
Kuwait	577<br>(447 to 729)	59.49<br>(46.5 to 75.12)	3193<br>(2488 to 4024)	85.34<br>(66.96 to 106.72)	43.46<br>(31.07 to 58.32)	1.13<br>(0.07 to 2.2)
Kyrgyzstan	116<br>(88 to 151)	3.75<br>(2.85 to 4.83)	604<br>(471 to 738)	12.13<br>(9.57 to 14.79)	223.47<br>(174.04 to 291.71)	4.12<br>(3.9 to 4.34)
Lao People's Democratic Republic	9<br>(6 to 11)	0.41<br>(0.31 to 0.53)	48<br>(35 to 66)	1.11<br>(0.82 to 1.49)	171.56<br>(133.35 to 211.94)	3.43<br>(3.29 to 3.57)
Latvia	1890<br>(1567 to 2295)	53.05<br>(44.04 to 63.93)	19331<br>(15857 to 23481)	526.67<br>(435.44 to 631.71)	892.84<br>(775.57 to 1047.38)	8.63<br>(8.22 to 9.04)
Lebanon	156<br>(113 to 221)	6.17<br>(4.49 to 8.69)	588<br>(437 to 783)	11.26<br>(8.34 to 15.04)	82.56<br>(58.11 to 114.82)	2.48<br>(2.07 to 2.89)
Lesotho	13<br>(10 to 17)	1.32<br>(1.06 to 1.65)	45<br>(35 to 56)	3.28<br>(2.6 to 4.08)	147.68<br>(115.45 to 189.28)	3.09<br>(2.9 to 3.29)
Liberia	14<br>(11 to 18)	1.16<br>(0.88 to 1.49)	58<br>(43 to 76)	2.18<br>(1.65 to 2.81)	87.45<br>(67.51 to 115.99)	2.32<br>(2.19 to 2.45)

Libya	229<br>(173 to 291)	10.87<br>(8.23 to 13.9)	646<br>(489 to 828)	11.24<br>(8.49 to 14.42)	3.39<br>(-7.52 to 15.46)	-0.01<br>(-0.33 to 0.32)
Lithuania	1728<br>(1379 to 2093)	38.33<br>(30.69 to 46.11)	20324<br>(16803 to 24468)	380.23<br>(316.79 to 452.87)	892.08<br>(756.03 to 1064.18)	8.28<br>(8.06 to 8.5)
Luxembourg	85<br>(67 to 110)	15.33<br>(12.1 to 19.62)	2621<br>(2133 to 3210)	264.1<br>(215.26 to 323.76)	1623.26<br>(1387.11 to 1929.31)	10.49<br>(9.1 to 11.9)
Madagascar	76<br>(62 to 94)	1.3<br>(1.05 to 1.57)	188<br>(149 to 235)	1.43<br>(1.15 to 1.75)	10.12<br>(0.71 to 19.64)	0.25<br>(0.16 to 0.33)
Malawi	35<br>(26 to 45)	0.85<br>(0.65 to 1.08)	95<br>(71 to 123)	1.19<br>(0.91 to 1.53)	40.47<br>(28.05 to 56.29)	1.13<br>(0.92 to 1.34)
Malaysia	131<br>(96 to 168)	1.48<br>(1.11 to 1.91)	2872<br>(2240 to 3646)	10.91<br>(8.63 to 13.67)	635.5<br>(509.49 to 803.04)	7.4<br>(6.93 to 7.87)
Maldives	1<br>(1 to 1)	0.9<br>(0.68 to 1.16)	19<br>(15 to 25)	6.77<br>(5.23 to 8.68)	654.19<br>(519.47 to 809.67)	6.8<br>(6.27 to 7.34)
Mali	41<br>(32 to 53)	0.87<br>(0.68 to 1.11)	115<br>(89 to 146)	1.11<br>(0.86 to 1.39)	27.66<br>(13.45 to 43.02)	0.76<br>(0.62 to 0.9)
Malta	15<br>(11 to 19)	3.48<br>(2.63 to 4.49)	958<br>(806 to 1156)	99.79<br>(83.46 to 119.84)	2770.62<br>(2183.09 to 3508.7)	12.91<br>(11.26 to 14.58)
Marshall Islands	0<br>(0 to 1)	2.51<br>(1.99 to 3.24)	3<br>(2 to 3)	8.03<br>(6.34 to 10.14)	219.37<br>(178.34 to 272.44)	3.52<br>(2.86 to 4.18)
Mauritania	21<br>(16 to 26)	1.86<br>(1.45 to 2.31)	80<br>(61 to 101)	3.33<br>(2.55 to 4.2)	78.98<br>(53.68 to 108.49)	2.08<br>(1.91 to 2.25)
Mauritius	12<br>(9 to 16)	1.68<br>(1.28 to 2.16)	252<br>(195 to 326)	14.25<br>(11.15 to 18.11)	748.69<br>(593.11 to 960.55)	7.21<br>(6.73 to 7.69)
Mexico	4473<br>(3540 to 5478)	9.96<br>(7.87 to 12.2)	50230<br>(40976 to 60266)	41.39<br>(33.68 to 49.4)	315.59<br>(275.5 to 364.69)	5.51<br>(3.85 to 7.2)
Micronesia (Federated States of)	2<br>(2 to 3)	5.13<br>(3.93 to 6.61)	14<br>(11 to 18)	22.73<br>(18.04 to 28.83)	343.06<br>(267.26 to 432.06)	4.49<br>(3.7 to 5.28)
Monaco	9<br>(7 to 12)	12.96<br>(10.11 to 17.05)	266<br>(220 to 329)	291.41<br>(242.55 to 359.55)	2148.61<br>(1738.83 to 2682.69)	11.62<br>(10.72 to 12.53)

Mongolia	30 (23 to 39)	2.73 (2.08 to 3.54)	312 (246 to 388)	11.74 (9.38 to 14.38)	330 (262.31 to 423.98)	5.06 (4.69 to 5.43)
Montenegro	678 (535 to 836)	108.71 (86.03 to 134.34)	4187 (3402 to 5087)	433.64 (352.66 to 524.66)	298.89 (251.37 to 352.96)	5.01 (4.82 to 5.2)
Morocco	282 (215 to 360)	1.83 (1.39 to 2.32)	1125 (869 to 1413)	3.24 (2.5 to 4.04)	77.15 (56.98 to 99.87)	1.99 (1.91 to 2.07)
Mozambique	47 (36 to 60)	0.73 (0.57 to 0.92)	148 (116 to 187)	1.22 (0.96 to 1.51)	67.59 (52.03 to 86)	1.86 (1.7 to 2.02)
Myanmar	95 (70 to 128)	0.4 (0.3 to 0.54)	554 (399 to 756)	1.2 (0.87 to 1.6)	195.18 (153.14 to 246.82)	3.73 (3.65 to 3.81)
Namibia	12 (9 to 14)	1.56 (1.25 to 1.92)	43 (34 to 53)	2.9 (2.34 to 3.54)	86.29 (70.94 to 106)	2.2 (2.09 to 2.31)
Nauru	0 (0 to 0)	5.56 (4.36 to 7.24)	1 (0 to 1)	17.2 (13.58 to 21.33)	209.13 (166.34 to 262.44)	3.22 (2.41 to 4.04)
Nepal	98 (74 to 127)	0.94 (0.73 to 1.21)	497 (382 to 626)	2.12 (1.65 to 2.67)	126 (101.25 to 151.98)	2.89 (2.75 to 3.04)
Netherlands	731 (572 to 931)	3.66 (2.86 to 4.62)	10494 (8327 to 13411)	30.87 (24.52 to 39.18)	743.57 (603.22 to 931.99)	7.71 (7.13 to 8.29)
New Zealand	3312 (2685 to 4130)	84.34 (68.75 to 104.19)	32328 (27212 to 38143)	405.99 (342.78 to 477.05)	381.37 (333.24 to 438.39)	5.99 (5.36 to 6.62)
Nicaragua	54 (42 to 67)	3.12 (2.46 to 3.89)	512 (403 to 632)	10.36 (8.19 to 12.7)	232.4 (188.47 to 289.12)	4.27 (4.14 to 4.4)
Niger	30 (23 to 39)	0.85 (0.65 to 1.08)	96 (73 to 125)	0.98 (0.75 to 1.25)	15.87 (4.55 to 29.75)	0.46 (0.4 to 0.51)
Nigeria	714 (545 to 908)	1.4 (1.08 to 1.76)	1762 (1345 to 2233)	1.6 (1.24 to 2.01)	14.34 (9.26 to 19.47)	0.37 (0.18 to 0.57)
Niue	0 (0 to 0)	5.82 (4.59 to 7.26)	1 (1 to 1)	49.02 (38.72 to 60.88)	742.93 (598.26 to 919.75)	7.03 (6.48 to 7.59)
North Macedonia	876 (693 to 1084)	46.36 (37.01 to 56.91)	9898 (8139 to 11957)	307.54 (253.65 to 370.57)	563.41 (460.12 to 708.83)	6.92 (6.63 to 7.21)

Northern Mariana Islands	5<br>(4 to 6)	34.32<br>(27.14 to 43.3)	65<br>(49 to 82)	128.64<br>(102.16 to 159.79)	274.79<br>(210.07 to 348.57)	3.51<br>(2.38 to 4.65)
Norway	753<br>(598 to 963)	11.08<br>(8.81 to 14.1)	5517<br>(4329 to 7066)	57.49<br>(45.28 to 73.06)	418.88<br>(355.26 to 499.74)	5.99<br>(5.31 to 6.68)
Oman	32<br>(25 to 41)	3.69<br>(2.9 to 4.55)	320<br>(237 to 413)	11.96<br>(9.11 to 15.24)	224.28<br>(170.69 to 287.73)	4.01<br>(3.67 to 4.36)
Pakistan	1502<br>(1181 to 1912)	2.53<br>(1.98 to 3.23)	4106<br>(3216 to 5110)	3.41<br>(2.69 to 4.25)	34.7<br>(26.4 to 43.55)	0.98<br>(0.74 to 1.23)
Palau	1<br>(0 to 1)	6.56<br>(4.98 to 8.42)	12<br>(9 to 14)	56.57<br>(44.48 to 71.27)	762.94<br>(590.56 to 971.91)	7.23<br>(6.59 to 7.88)
Palestine	30<br>(22 to 40)	3.15<br>(2.31 to 4.11)	96<br>(70 to 128)	3.29<br>(2.43 to 4.3)	4.22<br>(-15.29 to 21.96)	0.09<br>(-0.96 to 1.16)
Panama	85<br>(67 to 105)	5.44<br>(4.29 to 6.82)	635<br>(517 to 772)	15.29<br>(12.49 to 18.55)	180.78<br>(138.15 to 230.99)	3.31<br>(3.03 to 3.59)
Papua New Guinea	35<br>(28 to 45)	2.03<br>(1.59 to 2.61)	241<br>(186 to 300)	5.13<br>(3.99 to 6.35)	153.11<br>(123.16 to 189.15)	2.8<br>(2.32 to 3.28)
Paraguay	129<br>(105 to 157)	5.4<br>(4.4 to 6.57)	1448<br>(1203 to 1725)	25.43<br>(21.13 to 30.44)	371.11<br>(311.01 to 446.58)	5.56<br>(5.41 to 5.71)
Peru	377<br>(297 to 478)	2.91<br>(2.3 to 3.64)	11562<br>(9339 to 14013)	35.9<br>(29.07 to 43.6)	1133.55<br>(921.75 to 1393.78)	9.13<br>(8.77 to 9.48)
Philippines	187<br>(135 to 256)	0.6<br>(0.44 to 0.82)	1364<br>(997 to 1821)	1.73<br>(1.28 to 2.31)	186.99<br>(157.18 to 222.53)	3.67<br>(3.57 to 3.78)
Poland	49436<br>(39196 to 59539)	113.21<br>(89.83 to 135.96)	258294<br>(209260 to 305720)	382.71<br>(310.52 to 454.22)	238.06<br>(215.55 to 265.1)	4.4<br>(3.92 to 4.89)
Portugal	303<br>(228 to 402)	2.15<br>(1.63 to 2.86)	11252<br>(9003 to 14287)	46.47<br>(37.66 to 57.77)	2059.47<br>(1626.8 to 2611.12)	11.77<br>(10.93 to 12.62)
Puerto Rico	1944<br>(1573 to 2414)	53.47<br>(43.26 to 66.24)	28773<br>(22813 to 35815)	457.61<br>(362.96 to 575.53)	755.77<br>(599.99 to 940.83)	7.92<br>(7.17 to 8.68)
Qatar	283<br>(214 to 362)	108.48<br>(83.11 to 138.8)	2668<br>(2055 to 3339)	137.22<br>(105.59 to 171.6)	26.49<br>(12.78 to 41.67)	1.6<br>(0.32 to 2.89)

Republic of Korea	28188 (22924 to 34053)	92.66 (75.6 to 111.97)	268834 (226640 to 317268)	310.27 (261.14 to 364.94)	234.86 (186.12 to 295.02)	4.33 (4.2 to 4.45)
Republic of Moldova	704 (567 to 852)	15.88 (12.78 to 19.14)	15714 (12437 to 19128)	274.15 (219.12 to 333.46)	1626.14 (1354.47 to 1906.05)	10.76 (10.15 to 11.38)
Romania	32933 (26572 to 40249)	117.25 (94.72 to 142.48)	369313 (300496 to 454223)	1044.49 (855.59 to 1271.28)	790.81 (643.87 to 959.57)	8.36 (7.12 to 9.61)
Russian Federation	78538 (62246 to 97562)	42.95 (34.28 to 53.09)	1049884 (839793 to 1270534)	456.35 (365.58 to 553.15)	962.41 (882.47 to 1050.54)	8.64 (8.21 to 9.08)
Rwanda	32 (25 to 39)	1 (0.8 to 1.24)	79 (60 to 101)	1.22 (0.94 to 1.53)	21.51 (8.35 to 35.51)	0.7 (0.63 to 0.77)
Saint Kitts and Nevis	2 (2 to 3)	6.13 (5 to 7.47)	28 (22 to 33)	39.52 (32.26 to 47.2)	544.39 (457.33 to 644.44)	6.48 (6.32 to 6.65)
Saint Lucia	5 (4 to 6)	5.89 (4.84 to 7.13)	71 (59 to 86)	32.61 (26.88 to 39.31)	453.17 (373.43 to 557)	6.03 (5.97 to 6.1)
Saint Vincent and the Grenadines	4 (3 to 5)	4.95 (3.95 to 6.16)	42 (34 to 50)	30.25 (24.94 to 36.43)	510.85 (396.93 to 636.56)	6.44 (6.3 to 6.59)
Samoa	11 (9 to 13)	13.31 (10.55 to 16.77)	36 (29 to 45)	26.44 (21.08 to 33.1)	98.68 (72.93 to 132.31)	2.03 (1.14 to 2.92)
San Marino	2 (2 to 3)	6.38 (4.87 to 8.26)	109 (85 to 132)	169.43 (134.48 to 203.7)	2555.48 (2042.98 to 3187.78)	12.62 (10.8 to 14.47)
Sao Tome and Principe	1 (1 to 1)	1.48 (1.13 to 1.87)	4 (3 to 5)	2.76 (2.11 to 3.4)	86.39 (66.14 to 112.24)	2.2 (2.04 to 2.35)
Saudi Arabia	333 (237 to 450)	4.61 (3.32 to 6.39)	7647 (5831 to 9605)	30.66 (23.69 to 38.07)	564.54 (426.43 to 726.36)	6.76 (6.57 to 6.95)
Senegal	42 (33 to 54)	1.12 (0.88 to 1.42)	120 (92 to 152)	1.34 (1.05 to 1.7)	19.52 (6.93 to 32.71)	0.61 (0.53 to 0.7)
Serbia	6606 (5454 to 7854)	57.34 (48.14 to 67.6)	78339 (66345 to 92903)	492.89 (417.41 to 579.51)	759.65 (628.89 to 930.8)	7.99 (7.61 to 8.37)
Seychelles	2 (1 to 2)	2.79 (2.1 to 3.6)	23 (17 to 30)	20.89 (15.47 to 27.26)	649.55 (502.33 to 850.84)	7.29 (6.17 to 8.41)

Sierra Leone	22<br>(17 to 28)	1.04<br>(0.81 to 1.31)	57<br>(44 to 74)	1.27<br>(1 to 1.6)	22.53<br>(10.23 to 35.13)	0.66<br>(0.48 to 0.85)
Singapore	2259<br>(1849 to 2746)	98.11<br>(80.67 to 120.32)	34092<br>(28074 to 40796)	433.97<br>(358.54 to 518.38)	342.34<br>(298.84 to 384.98)	5.32<br>(5.17 to 5.47)
Slovakia	2434<br>(1962 to 3038)	40.65<br>(32.66 to 50.46)	36995<br>(30865 to 43899)	410.75<br>(342.55 to 482.3)	910.55<br>(755.32 to 1096.75)	9.43<br>(8.53 to 10.33)
Slovenia	2708<br>(2152 to 3350)	110.19<br>(87.57 to 136.18)	44251<br>(33263 to 56437)	1080.06<br>(825.88 to 1367.13)	880.19<br>(684.88 to 1152.6)	8.48<br>(8.18 to 8.78)
Solomon Islands	3<br>(2 to 4)	2.37<br>(1.84 to 3.1)	19<br>(15 to 24)	6.73<br>(5.28 to 8.49)	183.58<br>(142.41 to 231.51)	3.29<br>(2.92 to 3.66)
Somalia	23<br>(17 to 29)	0.78<br>(0.62 to 0.98)	53<br>(40 to 70)	0.66<br>(0.52 to 0.85)	-14.99<br>(-23.51 to -5.58)	-0.53<br>(-0.58 to -0.48)
South Africa	2243<br>(1747 to 2788)	10.2<br>(7.92 to 12.73)	56993<br>(43896 to 72883)	118.85<br>(91.51 to 151.35)	1065.04<br>(879.05 to 1286.31)	9.06<br>(8.66 to 9.47)
South Sudan	30<br>(23 to 38)	1.14<br>(0.88 to 1.45)	53<br>(40 to 70)	1.21<br>(0.92 to 1.56)	6.3<br>(-4.2 to 18.78)	0.24<br>(-0.02 to 0.5)
Spain	3319<br>(2599 to 4182)	6.14<br>(4.83 to 7.7)	82884<br>(66764 to 102702)	85.32<br>(69.27 to 105.43)	1288.85<br>(1052.65 to 1560.36)	9.84<br>(8.72 to 10.98)
Sri Lanka	175<br>(132 to 224)	1.61<br>(1.22 to 2.06)	1070<br>(818 to 1354)	4.07<br>(3.16 to 5.14)	152.29<br>(117.89 to 195.81)	3.1<br>(2.94 to 3.25)
Sudan	137<br>(105 to 175)	1.31<br>(1.01 to 1.68)	508<br>(390 to 646)	2.33<br>(1.82 to 2.97)	78.14<br>(59.81 to 99.6)	2.04<br>(1.89 to 2.19)
Suriname	13<br>(10 to 15)	4.6<br>(3.7 to 5.57)	170<br>(137 to 206)	27.52<br>(22.3 to 33.2)	498.04<br>(420.68 to 590.92)	6.41<br>(6.3 to 6.51)
Sweden	5508<br>(4430 to 6984)	35.49<br>(28.84 to 44.74)	60975<br>(49804 to 74278)	284.59<br>(234.8 to 343.78)	701.81<br>(617.85 to 804.45)	7.07<br>(6.38 to 7.75)
Switzerland	1277<br>(1002 to 1664)	12.01<br>(9.47 to 15.65)	43794<br>(36987 to 51577)	248.78<br>(210.62 to 294.25)	1971.85<br>(1629.06 to 2415.51)	11.22<br>(10.1 to 12.37)
Syrian Arab Republic	397<br>(314 to 490)	6.71<br>(5.29 to 8.26)	760<br>(594 to 947)	5.57<br>(4.4 to 6.86)	-17.02<br>(-27.59 to -5.67)	-0.57<br>(-0.73 to -0.41)

Taiwan (Province of China)	337<br>(250 to 448)	2.31<br>(1.75 to 2.98)	21911<br>(18162 to 26764)	55.11<br>(45.84 to 66.97)	2287.18<br>(1814.11 to 2898.3)	11.45<br>(10.82 to 12.08)
Tajikistan	71<br>(53 to 94)	2.4<br>(1.79 to 3.16)	353<br>(273 to 461)	5.72<br>(4.47 to 7.19)	138.82<br>(99.67 to 185.01)	3.03<br>(2.96 to 3.1)
Thailand	338<br>(250 to 447)	0.97<br>(0.74 to 1.26)	9793<br>(7633 to 12303)	9.52<br>(7.44 to 11.92)	879.65<br>(706.15 to 1133.02)	8.16<br>(8.03 to 8.29)
Timor-Leste	1<br>(1 to 2)	0.42<br>(0.31 to 0.56)	10<br>(7 to 13)	1.18<br>(0.88 to 1.55)	178.43<br>(138.22 to 235.3)	3.54<br>(3.43 to 3.65)
Togo	18<br>(14 to 22)	1.13<br>(0.89 to 1.4)	70<br>(54 to 88)	1.48<br>(1.17 to 1.82)	31.72<br>(20.74 to 45.85)	0.93<br>(0.76 to 1.1)
Tokelau	0<br>(0 to 0)	2.77<br>(2.2 to 3.49)	0<br>(0 to 0)	18.42<br>(14.59 to 23.16)	564.71<br>(448.07 to 714.72)	6.45<br>(6.08 to 6.82)
Tonga	4<br>(3 to 5)	8.28<br>(6.5 to 10.65)	27<br>(22 to 34)	35.43<br>(28.24 to 44.19)	328.04<br>(261.78 to 408.37)	4.15<br>(2.99 to 5.32)
Trinidad and Tobago	60<br>(47 to 75)	6.83<br>(5.36 to 8.54)	826<br>(656 to 1019)	43.89<br>(35.16 to 53.79)	542.82<br>(440.31 to 679.41)	7.07<br>(6.41 to 7.74)
Tunisia	226<br>(176 to 284)	4.04<br>(3.17 to 5.07)	847<br>(664 to 1071)	6.38<br>(5 to 8.03)	58.03<br>(42.25 to 77.12)	1.72<br>(1.54 to 1.9)
Turkey	1956<br>(1540 to 2409)	4.96<br>(3.95 to 6.04)	11167<br>(8897 to 13574)	12.3<br>(9.82 to 14.89)	147.97<br>(117.94 to 183.13)	3.19<br>(2.77 to 3.61)
Turkmenistan	75<br>(56 to 98)	3.78<br>(2.85 to 4.9)	1859<br>(1475 to 2277)	44.16<br>(35.28 to 53.39)	1068.28<br>(842.1 to 1362.16)	8.8<br>(8.56 to 9.04)
Tuvalu	0<br>(0 to 0)	2.06<br>(1.61 to 2.6)	1<br>(1 to 1)	9.09<br>(7.06 to 11.4)	340.83<br>(276.58 to 416.85)	4.77<br>(4.28 to 5.26)
Uganda	58<br>(44 to 75)	0.83<br>(0.64 to 1.05)	166<br>(127 to 213)	1.06<br>(0.82 to 1.33)	27.94<br>(17.03 to 41.69)	0.77<br>(0.63 to 0.92)
Ukraine	34725<br>(27665 to 42099)	49.15<br>(39.26 to 59.28)	168866<br>(133883 to 206429)	232.44<br>(185.06 to 282.36)	372.91<br>(333.16 to 420.12)	5.63<br>(5.31 to 5.95)
United Arab Emirates	170<br>(125 to 230)	18.86<br>(14.42 to 24.46)	5738<br>(4365 to 7301)	69.23<br>(53.5 to 88.41)	267.14<br>(223.05 to 328.04)	4.18<br>(2.72 to 5.67)

United Kingdom	108479 (86136 to 137223)	118.65 (94.51 to 149.28)	431376 (360325 to 516765)	341.24 (287.07 to 405.19)	187.6 (153.66 to 228.41)	3.82 (3.4 to 4.24)
United Republic of Tanzania	122 (92 to 156)	1.06 (0.81 to 1.33)	409 (311 to 524)	1.54 (1.18 to 1.95)	45.81 (31.22 to 62.55)	1.35 (1.2 to 1.49)
United States of America	685738 (556401 to 838473)	210.23 (171.24 to 256.04)	1425073 (1243341 to 1649908)	261.92 (229.32 to 299.88)	24.58 (11.1 to 41.3)	1.77 (0.72 to 2.84)
United States Virgin Islands	30 (24 to 37)	32.95 (27 to 40.62)	484 (388 to 595)	263.88 (211.63 to 324)	700.74 (581.21 to 833.25)	7.26 (6.81 to 7.72)
Uruguay	494 (381 to 645)	12.71 (9.88 to 16.44)	7001 (5879 to 8386)	129.03 (108.73 to 153.35)	914.83 (700.14 to 1168.81)	8.05 (7.75 to 8.35)
Uzbekistan	427 (319 to 557)	3.65 (2.74 to 4.73)	7733 (6102 to 9525)	33.6 (27.16 to 40.4)	819.39 (646.32 to 1044.74)	8.08 (7.83 to 8.33)
Vanuatu	2 (1 to 2)	3.01 (2.35 to 3.9)	14 (11 to 18)	8.39 (6.57 to 10.66)	178.53 (141.7 to 226.35)	3.28 (2.92 to 3.64)
Venezuela (Bolivarian Republic of)	655 (534 to 788)	6.51 (5.29 to 7.84)	6325 (5185 to 7603)	21.17 (17.36 to 25.42)	225.06 (185.25 to 268.49)	4.08 (3.77 to 4.4)
Viet Nam	257 (193 to 342)	0.63 (0.48 to 0.83)	1796 (1333 to 2368)	1.93 (1.44 to 2.52)	204.44 (161.9 to 259.87)	3.84 (3.73 to 3.94)
Yemen	75 (59 to 96)	1.27 (1 to 1.61)	268 (208 to 347)	1.67 (1.31 to 2.11)	30.75 (18.1 to 45.41)	0.94 (0.88 to 1)
Zambia	28 (21 to 36)	0.91 (0.7 to 1.17)	103 (78 to 133)	1.38 (1.06 to 1.76)	51.8 (36.32 to 70.3)	1.49 (1.2 to 1.78)
Zimbabwe	77 (60 to 96)	1.77 (1.39 to 2.21)	172 (138 to 210)	2.27 (1.83 to 2.77)	28.18 (16.54 to 41.04)	0.79 (0.59 to 0.98)

AAPC, average annual percentage change; ASPR, age-standardized prevalence rate; SDI, sociodemographic index; UI, uncertainty interval; CI, confidence interval.
