# Supplementary material for: Global Burden and Improvement Gap of Non-Rheumatic Calcific Aortic Valve Disease: 1990–2019 Findings from Global Burden of Disease Study 2019
Source: J Clin Med. 2022 Nov 14;11(22):6733. doi: 10.3390/jcm11226733 (PMC9698619; doi:10.3390/jcm11226733)
Supplement: Supplementary file 1 [file jcm-11-06733-s001.zip › Supplemental Table S2 incidence calcific aortic valve disease.pdf]

Table S2. The incidence cases and age-standardized incidence rate of non-rheumatic calcific aortic valve disease in 1990 and 2019 and its temporal trends from 1990 to 2019.

	1990		2019		1990–2019	1990–2019
	Cases<br>No. (95% UI)	ASIR (per 100,000)<br>No. (95% UI)	Cases<br>No. (95% UI)	ASIR (per 100,000)<br>No. (95% UI)	ASIR Percentage change<br>(ASIR, 95% UI)	AAPC<br>No. (95% CI)
Global	130822<br>(110701 to 156022)	3.25<br>(2.76 to 3.86)	589638<br>(512895 to 677062)	7.13<br>(6.22 to 8.15)	119.24<br>(108.72 to 131.69)	2.85<br>(2.44 to 3.26)
Female	61178<br>(50969 to 73841)	2.88<br>(2.41 to 3.45)	275833<br>(239867 to 317136)	6.31<br>(5.49 to 7.26)	119.27<br>(107.45 to 134.6)	2.9<br>(2.58 to 3.22)
Male	69644<br>(59317 to 82577)	3.63<br>(3.11 to 4.27)	313805<br>(271313 to 360915)	7.95<br>(6.92 to 9.09)	119.05<br>(108.57 to 130.95)	2.82<br>(2.37 to 3.27)
High SDI	92683<br>(76421 to 112271)	9.18<br>(7.65 to 11.05)	329823<br>(285980 to 381248)	19.04<br>(16.65 to 21.96)	107.46<br>(94.77 to 121.84)	2.63<br>(2.19 to 3.08)
High-middle SDI	26389<br>(22287 to 31007)	2.4<br>(2.04 to 2.79)	193217<br>(163540 to 225654)	9.59<br>(8.14 to 11.18)	298.74<br>(283.11 to 316)	4.95<br>(4.72 to 5.19)
Middle SDI	6192<br>(5244 to 7327)	0.63<br>(0.54 to 0.73)	49938<br>(41587 to 59436)	1.89<br>(1.59 to 2.23)	182.34<br>(156.46 to 215.14)	3.86<br>(3.66 to 4.06)
Low-middle SDI	3905<br>(3335 to 4608)	0.77<br>(0.66 to 0.9)	12597<br>(10820 to 14600)	0.98<br>(0.85 to 1.14)	26.78<br>(23.12 to 31.13)	0.83<br>(0.76 to 0.9)
Low SDI	1618<br>(1390 to 1879)	0.82<br>(0.71 to 0.94)	3860<br>(3343 to 4455)	0.84<br>(0.73 to 0.97)	-3.56<br>(-5.15 to -1.93)	0.1<br>(0.07 to 0.14)
High-income Asia Pacific	30473<br>(25249 to 36916)	15.12<br>(12.61 to 18.17)	88601<br>(73613 to 107125)	25.04<br>(21.32 to 29.6)	65.55<br>(55.74 to 75.72)	1.76<br>(1.68 to 1.85)
High-income North America	48905<br>(39764 to 59971)	14.32<br>(11.71 to 17.4)	123568<br>(106451 to 142325)	20.23<br>(17.61 to 23.2)	41.24<br>(25.04 to 60.36)	2.21<br>(1.21 to 3.23)
Western Europe	15951<br>(13209 to 19243)	2.96<br>(2.46 to 3.57)	116514<br>(98950 to 138347)	14.9<br>(12.77 to 17.56)	403.33<br>(367.91 to 444.76)	6.12<br>(5.55 to 6.69)
Australasia	1260<br>(1103 to 1452)	5.56<br>(4.9 to 6.32)	20173<br>(17134 to 23512)	44.39<br>(38.08 to 51.79)	698.05<br>(597.68 to 830.16)	7.46<br>(7.31 to 7.61)
Andean Latin America	152<br>(134 to 173)	0.72<br>(0.65 to 0.81)	2147<br>(1861 to 2465)	3.62<br>(3.14 to 4.16)	400.47<br>(344.34 to 463.24)	6.12<br>(5.63 to 6.6)

Tropical Latin America	1950<br>(1674 to 2279)	2.22<br>(1.91 to 2.57)	6585<br>(5610 to 7682)	2.71<br>(2.31 to 3.15)	21.64<br>(14.55 to 30.02)	0.65<br>(0.57 to 0.73)
Central Latin America	1051<br>(916 to 1201)	1.19<br>(1.05 to 1.35)	5750<br>(4954 to 6617)	2.29<br>(1.99 to 2.63)	93.42<br>(78.8 to 108.5)	2.39<br>(1.53 to 3.26)
Southern Latin America	1088<br>(897 to 1431)	2.59<br>(2.13 to 3.46)	8727<br>(7730 to 9923)	10.64<br>(9.44 to 12.08)	311.48<br>(214.61 to 401.02)	4.93<br>(4.51 to 5.35)
Caribbean	443<br>(398 to 493)	1.7<br>(1.53 to 1.88)	2350<br>(2015 to 2734)	4.49<br>(3.86 to 5.21)	164.97<br>(135.37 to 193.6)	3.52<br>(3.24 to 3.81)
Central Europe	9801<br>(8263 to 11497)	6.59<br>(5.52 to 7.69)	57281<br>(48866 to 67322)	33.16<br>(28.29 to 38.67)	403.37<br>(358.25 to 448.46)	6.11<br>(5.72 to 6.5)
Eastern Europe	7933<br>(6360 to 9591)	2.9<br>(2.34 to 3.48)	68415<br>(55228 to 82915)	22.58<br>(18.38 to 27.04)	678.78<br>(631.44 to 732.11)	7.51<br>(7.3 to 7.72)
Central Asia	330<br>(268 to 403)	0.66<br>(0.54 to 0.8)	2645<br>(2183 to 3113)	2.86<br>(2.37 to 3.35)	335.57<br>(282.18 to 389.39)	5.16<br>(4.96 to 5.36)
North Africa and Middle East	2036<br>(1746 to 2369)	1.19<br>(1.04 to 1.36)	7070<br>(5978 to 8346)	1.46<br>(1.27 to 1.69)	22.68<br>(16.65 to 29.87)	0.99<br>(0.59 to 1.4)
South Asia	4129<br>(3483 to 4942)	0.93<br>(0.79 to 1.09)	12370<br>(10486 to 14621)	1.01<br>(0.85 to 1.18)	7.86<br>(5.96 to 9.56)	0.29<br>(0.23 to 0.35)
Southeast Asia	713<br>(593 to 862)	0.32<br>(0.27 to 0.38)	3295<br>(2778 to 3940)	0.54<br>(0.46 to 0.64)	69.72<br>(56.21 to 84.64)	1.85<br>(1.8 to 1.89)
East Asia	2971<br>(2366 to 3782)	0.34<br>(0.28 to 0.42)	56641<br>(45299 to 69328)	2.61<br>(2.11 to 3.15)	665.55<br>(544.2 to 807.08)	7.18<br>(6.96 to 7.41)
Oceania	28<br>(24 to 33)	1.07<br>(0.91 to 1.26)	130<br>(109 to 153)	1.73<br>(1.47 to 2.04)	61.9<br>(46.07 to 80.31)	1.42<br>(1.13 to 1.71)
Western Sub-Saharan Africa	505<br>(429 to 597)	0.57<br>(0.49 to 0.66)	1160<br>(992 to 1365)	0.58<br>(0.5 to 0.67)	1.73<br>(-0.39 to 3.81)	0.08<br>(0.05 to 0.1)
Eastern Sub-Saharan Africa	500<br>(433 to 575)	0.83<br>(0.74 to 0.95)	1166<br>(1015 to 1351)	0.84<br>(0.73 to 0.96)	0.6<br>(-1.77 to 3.41)	0.05<br>(0.01 to 0.09)

Central Sub-Saharan Africa	163<br>(142 to 187)	0.94<br>(0.84 to 1.06)	411<br>(360 to 472)	0.97<br>(0.87 to 1.1)	3.47<br>(-0.48 to 6.92)	0.15<br>(0.11 to 0.19)
Southern Sub-Saharan Africa	441<br>(374 to 516)	1.62<br>(1.38 to 1.91)	4637<br>(3717 to 5702)	7.09<br>(5.73 to 8.6)	337.49<br>(269.44 to 416.62)	5.68<br>(5.08 to 6.28)

AAPC, average annual percentage change; ASIR, age-standardised incidence rates; SDI, sociodemographic index; UI, uncertainty interval; CI, confidence interval.
