# Supplementary material for: Global Burden and Improvement Gap of Non-Rheumatic Calcific Aortic Valve Disease: 1990–2019 Findings from Global Burden of Disease Study 2019
Source: J Clin Med. 2022 Nov 14;11(22):6733. doi: 10.3390/jcm11226733 (PMC9698619; doi:10.3390/jcm11226733)
Supplement: Supplementary file 1 [file jcm-11-06733-s001.zip › Supplemental Table S3 DALYs calcific aortic valve disease.pdf]

Table S3. The DALYs and age-standardized DALYs rate of non-rheumatic calcific aortic valve disease in 1990 and 2019 and its temporal trends from 1990 to 2019.

	1990		2019		1990–2019	1990–2019
	Cases No. (95% UI)	Age-standardized DALY rate (per 100,000) No. (95% UI)	Cases No. (95% UI)	Age-standardized DALY rate (per 100,000) No. (95% UI)	Age-standardized DALY rate Percentage change (95% UI)	AAPC No. (95% CI)
Global	975894 (872009 to 1109788)	26.85 (24.07 to 30.31)	1837751 (1637023 to 2031852)	23.9 (21.1 to 26.55)	-10.97 (-17.94 to -3.46)	-0.33 (-0.48 to -0.19)
Female	442232 (381940 to 551815)	22.3 (19.27 to 27.31)	893719 (765940 to 1032792)	20.48 (17.55 to 23.69)	-8.18 (-17.87 to 1.21)	-0.22 (-0.39 to -0.04)
Male	533661 (481123 to 594233)	31.47 (28.7 to 34.63)	944032 (864570 to 1036413)	27.4 (24.8 to 29.99)	-12.94 (-19.92 to -4.25)	-0.42 (-0.56 to -0.29)
High SDI	553755 (508564 to 609841)	52.88 (48.59 to 58.27)	913672 (778952 to 1005197)	42.88 (37.5 to 47)	-18.9 (-24.94 to -13.51)	-0.68 (-0.78 to -0.57)
High-middle SDI	178055 (162774 to 197284)	17.25 (15.73 to 19.19)	395647 (353615 to 437845)	20.21 (18.01 to 22.35)	17.15 (2.71 to 29.36)	0.51 (0.38 to 0.64)
Middle SDI	121004 (98914 to 146117)	10.48 (8.62 to 12.68)	251024 (227014 to 281657)	10.18 (9.21 to 11.4)	-2.79 (-17.93 to 21.45)	-0.13 (-0.19 to -0.08)
Low-middle SDI	81108 (53195 to 111592)	12.97 (8.69 to 17.57)	187228 (149865 to 235314)	13.56 (10.9 to 16.84)	4.53 (-12.79 to 34.18)	0.16 (0.09 to 0.23)
Low SDI	41391 (22473 to 63108)	16.14 (9.28 to 23.73)	89078 (62680 to 119761)	15.8 (11.57 to 20.29)	-2.11 (-22.92 to 34.05)	-0.12 (-0.15 to -0.08)
High-income Asia Pacific	70903 (63434 to 81928)	38.17 (33.96 to 44.37)	142126 (109597 to 165943)	24.46 (19.89 to 28.22)	-35.91 (-45.57 to -27.93)	-1.49 (-1.81 to -1.16)
High-income North America	206708 (189977 to 224974)	57.8 (53.37 to 62.81)	314811 (274623 to 346354)	46.8 (41.53 to 51.25)	-19.04 (-24.18 to -14)	-0.73 (-0.93 to -0.54)
Western Europe	321786 (294953 to 357151)	55.48 (50.82 to 61.36)	543784 (471461 to 598088)	51.94 (45.69 to 56.95)	-6.38 (-14.12 to 1.55)	-0.17 (-0.3 to -0.03)
Australasia	12154 (11119 to 13476)	52.52 (47.85 to 58.16)	23975 (20443 to 27499)	44.54 (38.48 to 51.02)	-15.19 (-23.88 to -5.18)	-0.52 (-0.74 to -0.3)
Andean Latin	3304	13.67	7264	12.54	-8.29	-0.2

America	(2471 to 4283)	(10.36 to 17.42)	(5877 to 8871)	(10.21 to 15.28)	(-31.73 to 27.17)	(-0.41 to 0.01)
Tropical Latin America	43137<br>(38392 to 47877)	41.46<br>(36.63 to 45.36)	70731<br>(63569 to 80921)	29.5<br>(26.5 to 33.74)	-28.84<br>(-34.8 to -20.16)	-1.16<br>(-1.25 to -1.07)
Central Latin America	18125<br>(16449 to 20015)	18.85<br>(17.19 to 20.94)	44870<br>(36842 to 55560)	18.72<br>(15.4 to 23.13)	-0.73<br>(-17.42 to 22.44)	-0.07<br>(-0.36 to 0.23)
Southern Latin America	24229<br>(21712 to 27031)	54.79<br>(48.95 to 61.1)	42481<br>(38287 to 46438)	50.54<br>(45.69 to 55.27)	-7.77<br>(-18.99 to 4.37)	-0.16<br>(-0.32 to 0)
Caribbean	6313<br>(5372 to 7234)	22.61<br>(19.31 to 25.69)	11324<br>(9158 to 14157)	22.09<br>(17.87 to 27.65)	-2.27<br>(-18.67 to 16.82)	0<br>(-0.18 to 0.18)
Central Europe	20994<br>(18132 to 25377)	14.59<br>(12.57 to 17.61)	84108<br>(69743 to 99017)	39.15<br>(32.37 to 46.15)	168.33<br>(93.69 to 224.57)	3.6<br>(3.25 to 3.94)
Eastern Europe	14221<br>(11028 to 16353)	5.25<br>(4.11 to 6.02)	46125<br>(37773 to 56568)	14.17<br>(11.57 to 17.34)	169.8<br>(114.91 to 265.33)	3.42<br>(2.68 to 4.17)
Central Asia	1757<br>(1480 to 1956)	3.48<br>(2.89 to 3.92)	5538<br>(4671 to 6969)	7.45<br>(6.27 to 9.38)	113.88<br>(74.11 to 186.27)	2.58<br>(1.85 to 3.32)
North Africa and Middle East	49071<br>(36291 to 61474)	24.77<br>(18.02 to 31.02)	100971<br>(84200 to 119652)	21.11<br>(17.59 to 24.72)	-14.77<br>(-30.89 to 20.78)	-0.58<br>(-0.65 to -0.52)
South Asia	76867<br>(44619 to 111682)	13.86<br>(8.35 to 19.67)	190680<br>(144057 to 248601)	13.83<br>(10.63 to 17.66)	-0.25<br>(-21.64 to 38.44)	0.02<br>(-0.14 to 0.19)
Southeast Asia	17125<br>(14082 to 23373)	6.22<br>(5.14 to 8.24)	39382<br>(32225 to 50172)	6.49<br>(5.36 to 8.18)	4.38<br>(-16.05 to 35.53)	0.21<br>(0.13 to 0.29)
East Asia	46987<br>(30302 to 66132)	4.71<br>(3.06 to 6.62)	86816<br>(70962 to 103738)	4.36<br>(3.59 to 5.18)	-7.57<br>(-38.27 to 53.8)	-0.24<br>(-0.44 to -0.03)
Oceania	542<br>(322 to 826)	15.64<br>(9.68 to 23.28)	1275<br>(819 to 1885)	15.58<br>(10.6 to 22.16)	-0.37<br>(-19.88 to 25.89)	-0.01<br>(-0.12 to 0.09)
Western Sub-Saharan Africa	15626<br>(8040 to 24983)	15.2<br>(8 to 23.99)	31347<br>(21263 to 45261)	13.17<br>(9.11 to 18.49)	-13.36<br>(-35.16 to 27.51)	-0.49<br>(-0.59 to -0.4)
Eastern Sub-Saharan	15687<br>(9220 to 24319)	18.86<br>(12.21 to 27.88)	29973<br>(22891 to 39839)	16.91<br>(13.45 to 21.67)	-10.36<br>(-35.05 to 29.79)	-0.4<br>(-0.5 to -0.3)

Africa						
Central Sub-Saharan Africa	4595<br>(2589 to 7527)	19.38<br>(11.77 to 30.04)	10426<br>(6840 to 15597)	18.97<br>(13.23 to 26.91)	-2.11<br>(-29.55 to 39.69)	-0.11<br>(-0.16 to -0.05)
Southern Sub-Saharan Africa	5763<br>(4929 to 7016)	17.96<br>(15.51 to 21.18)	9744<br>(8365 to 11082)	17.24<br>(14.86 to 19.32)	-4.01<br>(-17.76 to 10.29)	-0.07<br>(-0.3 to 0.16)

AAPC, average annual percentage change; DALY, disability-adjusted life-years; SDI, sociodemographic index; UI, uncertainty interval; CI, confidence interval.
