# Supplementary material for: Global Burden and Improvement Gap of Non-Rheumatic Calcific Aortic Valve Disease: 1990–2019 Findings from Global Burden of Disease Study 2019
Source: J Clin Med. 2022 Nov 14;11(22):6733. doi: 10.3390/jcm11226733 (PMC9698619; doi:10.3390/jcm11226733)
Supplement: Supplementary file 1 [file jcm-11-06733-s001.zip › Supplemental Table S4 death calcific aortic valve disease.pdf]

Table S4. The death cases and age-standardized mortality rate of cardiomyopathy between 1990 and 2019 and its temporal trends from 1990 to 2019.

	1990		2019		1990–2019	1990–2019
	Cases No. (95% UI)	ASMR (per 100,000) No. (95% UI)	Cases No. (95% UI)	ASMR (per 100,000) No. (95% UI)	ASMR Percentage change (95% UI)	AAPC No. (95% CI)
Global	53298 (47760 to 59731)	1.75 (1.55 to 1.96)	126827 (105603 to 141390)	1.76 (1.45 to 1.97)	0.37 (-8.85 to 7.99)	0.01 (-0.24 to 0.27)
Female	28780 (24823 to 34071)	1.62 (1.39 to 1.91)	72652 (57761 to 84305)	1.66 (1.32 to 1.92)	2.05 (-6.84 to 10.75)	0.05 (-0.09 to 0.18)
Male	24517 (22439 to 27031)	1.86 (1.69 to 2.04)	54175 (47774 to 58670)	1.85 (1.58 to 2.01)	-0.74 (-9.2 to 9.14)	-0.15 (-0.39 to 0.1)
High SDI	36015 (32133 to 39860)	3.46 (3.06 to 3.84)	80211 (64300 to 90100)	3.35 (2.75 to 3.74)	-3.16 (-11.89 to 3.45)	-0.01 (-0.23 to 0.22)
High-middle SDI	8359 (7420 to 9206)	0.93 (0.81 to 1.04)	24437 (20864 to 27261)	1.28 (1.08 to 1.43)	36.93 (21.22 to 53.62)	1.03 (0.89 to 1.17)
Middle SDI	4270 (3502 to 5173)	0.47 (0.39 to 0.57)	10479 (9433 to 11740)	0.48 (0.43 to 0.53)	1.1 (-13.31 to 24.6)	0.02 (-0.05 to 0.08)
Low-middle SDI	3120 (2097 to 4200)	0.65 (0.44 to 0.85)	8166 (6628 to 9989)	0.7 (0.57 to 0.84)	7.45 (-10.49 to 36.84)	0.26 (0.18 to 0.33)
Low SDI	1503 (876 to 2199)	0.77 (0.47 to 1.09)	3465 (2557 to 4390)	0.8 (0.61 to 0.99)	3.66 (-18.37 to 38.65)	0.11 (0.07 to 0.15)
High-income Asia Pacific	4282 (3659 to 5067)	2.57 (2.15 to 3.06)	13573 (9085 to 16510)	1.98 (1.4 to 2.36)	-22.98 (-37.39 to -12.89)	-1.06 (-1.48 to -0.64)
High-income North America	13159 (11623 to 14395)	3.56 (3.14 to 3.89)	27187 (22160 to 30239)	3.64 (3.02 to 4.02)	2.29 (-4.73 to 7.84)	0.1 (-0.08 to 0.27)
Western Europe	21104 (18958 to 23334)	3.59 (3.21 to 3.97)	47894 (39520 to 53660)	4.05 (3.4 to 4.51)	12.79 (2.47 to 21.66)	0.41 (0.22 to 0.6)
Australasia	771 (686 to 852)	3.48 (3.07 to 3.85)	1866 (1514 to 2108)	3.18 (2.62 to 3.58)	-8.66 (-17.99 to -0.51)	-0.22 (-0.52 to 0.08)
Andean Latin America	118 (90 to 147)	0.58 (0.45 to 0.71)	291 (237 to 353)	0.53 (0.43 to 0.64)	-9.6 (-30.38 to 23.36)	-0.18 (-0.45 to 0.09)

Tropical Latin America	1533<br>(1350 to 1664)	1.81<br>(1.59 to 1.95)	3583<br>(3115 to 4139)	1.56<br>(1.34 to 1.8)	-14.04<br>(-22.45 to -2.22)	-0.47<br>(-0.56 to -0.37)
Central Latin America	657<br>(599 to 733)	0.81<br>(0.74 to 0.9)	1955<br>(1607 to 2430)	0.85<br>(0.7 to 1.06)	5.44<br>(-11.75 to 28.89)	0.17<br>(-0.09 to 0.43)
Southern Latin America	1386<br>(1216 to 1558)	3.43<br>(3.01 to 3.85)	2844<br>(2455 to 3158)	3.3<br>(2.85 to 3.66)	-3.94<br>(-15.56 to 9.78)	0.01<br>(-0.41 to 0.43)
Caribbean	253<br>(218 to 283)	1<br>(0.87 to 1.12)	516<br>(422 to 626)	1<br>(0.82 to 1.22)	-0.42<br>(-16.89 to 18.21)	0.13<br>(-0.05 to 0.32)
Central Europe	903<br>(751 to 1111)	0.66<br>(0.54 to 0.81)	4675<br>(3771 to 5584)	2.08<br>(1.68 to 2.49)	215.7<br>(125.02 to 305.48)	4.26<br>(3.74 to 4.78)
Eastern Europe	562<br>(439 to 632)	0.21<br>(0.17 to 0.24)	1640<br>(1374 to 1929)	0.48<br>(0.4 to 0.57)	125.97<br>(86.09 to 203.26)	2.67<br>(2.03 to 3.32)
Central Asia	63<br>(51 to 73)	0.14<br>(0.11 to 0.16)	207<br>(172 to 264)	0.33<br>(0.27 to 0.43)	138.86<br>(86.15 to 243.85)	3.09<br>(2.32 to 3.87)
North Africa and Middle East	1729<br>(1260 to 2179)	1.13<br>(0.82 to 1.45)	3819<br>(3193 to 4444)	0.97<br>(0.81 to 1.12)	-14.33<br>(-30.12 to 21.53)	-0.54<br>(-0.7 to -0.37)
South Asia	3063<br>(1848 to 4366)	0.75<br>(0.47 to 1.03)	8749<br>(6846 to 11048)	0.76<br>(0.6 to 0.95)	1.29<br>(-21.06 to 39.53)	0.02<br>(-0.33 to 0.38)
Southeast Asia	656<br>(542 to 862)	0.3<br>(0.24 to 0.38)	1703<br>(1401 to 2132)	0.33<br>(0.27 to 0.41)	10.36<br>(-9.53 to 43.36)	0.42<br>(0.29 to 0.55)
East Asia	1568<br>(1021 to 2224)	0.19<br>(0.12 to 0.27)	3231<br>(2621 to 3846)	0.17<br>(0.14 to 0.2)	-8.43<br>(-39.85 to 53.54)	-0.21<br>(-0.49 to 0.06)
Oceania	18<br>(11 to 26)	0.79<br>(0.52 to 1.12)	41<br>(28 to 59)	0.75<br>(0.54 to 1.04)	-4.56<br>(-23.57 to 19.87)	-0.19<br>(-0.32 to -0.05)
Western Sub-Saharan Africa	512<br>(272 to 815)	0.6<br>(0.33 to 0.95)	1018<br>(710 to 1424)	0.55<br>(0.39 to 0.76)	-8.36<br>(-29.83 to 31.38)	-0.31<br>(-0.39 to -0.23)
Eastern Sub-Saharan Africa	574<br>(377 to 850)	0.94<br>(0.66 to 1.34)	1195<br>(952 to 1527)	0.92<br>(0.72 to 1.21)	-1.97<br>(-27.46 to 34.25)	-0.09<br>(-0.17 to 0)

Central Sub-Saharan Africa	169 (102 to 263)	0.97 (0.63 to 1.42)	410 (287 to 579)	1.01 (0.74 to 1.36)	3.51 (-23.88 to 44.84)	0.09 (0.01 to 0.16)
Southern Sub-Saharan Africa	219 (190 to 253)	0.9 (0.78 to 1.04)	430 (371 to 480)	0.96 (0.82 to 1.07)	6.39 (-6.57 to 20.29)	0.38 (0.11 to 0.66)

AAPC, average annual percentage change; ASMR, age-standardized mortality rate; SDI, sociodemographic index; UI, uncertainty interval; CI, confidence interval.
