# Supplementary material for: Global Burden and Improvement Gap of Non-Rheumatic Calcific Aortic Valve Disease: 1990–2019 Findings from Global Burden of Disease Study 2019
Source: J Clin Med. 2022 Nov 14;11(22):6733. doi: 10.3390/jcm11226733 (PMC9698619; doi:10.3390/jcm11226733)
Supplement: Supplementary file 1 [file jcm-11-06733-s001.zip › Supplemental Table S5 Decomposition.pdf]

Table S5. Changes in DALYs number according to population-level determinants from 1990 to 2019.

| | Overll difference [a] | Change due to Population-level determinants (% contribute to the total changes) | | |
|---|---|---|---|---|
| | | Aging [b] | Population [c] | Epidemiological change [d] |
| Global | 861857.7 | 437441.997 (50.76%) | 644103.751 (74.73%) | -219688.069 (-25.49%) |
| **Sex** | | | | |
| Female | 451486.8 | 215325.588 (47.69%) | 304776.407 (67.51%) | -68615.229 (-15.2%) |
| Male | 410370.9 | 224626.884 (54.74%) | 338955.233 (82.6%) | -153211.204 (-37.33%) |
| **SDI** | | | | |
| High SDI | 359916.9 | 288905.882 (80.27%) | 192942.943 (53.61%) | -121931.97 (-33.88%) |
| High-middle SDI | 217592.1 | 92600.64 (42.56%) | 90376.124 (41.53%) | 34615.325 (15.91%) |
| Middle SDI | 130019.3 | 53562.555 (41.2%) | 85933.168 (66.09%) | -9476.461 (-7.29%) |
| Low-middle SDI | 106119.8 | 25221.164 (23.77%) | 76288.263 (71.89%) | 4610.351 (4.34%) |
| Low SDI | 47687.09 | -893.339 (-1.87%) | 51800.204 (108.63%) | -3219.775 (-6.75%) |
| **Region** | | | | |
| High-income Asia Pacific | 71223.22 | 93118.761 (130.74%) | 18124.499 (25.45%) | -40020.041 (-56.19%) |
| High-income North America | 108103.1 | 71409.878 (66.06%) | 78556.599 (72.67%) | -41863.367 (-38.73%) |
| Western Europe | 221998.1 | 155153.903 (69.89%) | 66599.498 (30%) | 244.706 (0.11%) |
| Australasia | 11821.17 | 7405.606 (62.65%) | 7038.341 (59.54%) | -2622.778 (-22.19%) |
| Andean Latin America | 3959.696 | 1113.853 (28.13%) | 3385.046 (85.49%) | -539.203 (-13.62%) |
| Tropical Latin America | 27593.39 | 18575.248 (67.32%) | 31821.137 (115.32%) | -22802.992 (-82.64%) |
| Central Latin America | 26745.57 | 9738.641 (36.41%) | 17894.263 (66.91%) | -887.333 (-3.32%) |
| Southern Latin America | 18251.93 | 7926.753 (43.43%) | 13089.269 (71.71%) | -2764.097 (-15.14%) |
| Caribbean | 5011.07 | 1883.318 (37.58%) | 3384.755 (67.55%) | -257.003 (-5.13%) |
| Central Europe | 63114.19 | 17727.122 (28.09%) | 1317.128 (2.09%) | 44069.939 (69.83%) |

| | | | | |
|---|---|---|---|---|
| Eastern Europe | 31903.89 | 5830.385 (18.27%) | -361.012 (-1.13%) | 26434.517 (82.86%) |
| Central Asia | 3780.755 | 146.945 (3.89%) | 1369.168 (36.21%) | 2264.642 (59.9%) |
| North Africa and Middle East | 51900.49 | 9165.91 (17.66%) | 55381.679 (106.71%) | -12647.098 (-24.37%) |
| South Asia | 113813.2 | 30766.423 (27.03%) | 83414.196 (73.29%) | -367.449 (-0.32%) |
| Southeast Asia | 22257.27 | 7404.699 (33.27%) | 14304.552 (64.27%) | 548.02 (2.46%) |
| East Asia | 39829.08 | 25869.873 (64.95%) | 21504.02 (53.99%) | -7544.813 (-18.94%) |
| Oceania | 732.757 | 33.284 (4.54%) | 672.739 (91.81%) | 26.734 (3.65%) |
| Western Sub-Saharan Africa | 15720.76 | -1242.489 (-7.9%) | 20713.191 (131.76%) | -3749.943 (-23.85%) |
| Eastern Sub-Saharan Africa | 14286.68 | -853.876 (-5.98%) | 19254.765 (134.77%) | -4114.213 (-28.8%) |
| Central Sub-Saharan Africa | 5830.604 | -267.014 (-4.58%) | 6595.087 (113.11%) | -497.469 (-8.53%) |
| Southern Sub-Saharan Africa | 3980.776 | 954.833 (23.99%) | 4076.031 (102.39%) | -1050.088 (-26.38%) |
| **Nation** | | | | |
| Afghanistan | 2168.015 | -939.417 (-43.33%) | 3177.126 (146.55%) | -69.694 (-3.21%) |
| Albania | 326.783 | 206.084 (63.06%) | 8.458 (2.59%) | 112.241 (34.35%) |
| Algeria | 4078.619 | 1196.225 (29.33%) | 3859.283 (94.62%) | -976.889 (-23.95%) |
| American Samoa | 5.737 | 2.432 (42.39%) | 2.189 (38.16%) | 1.116 (19.45%) |
| Andorra | 26.768 | 15.543 (58.07%) | 11.403 (42.6%) | -0.178 (-0.66%) |
| Angola | 1477.668 | 14.358 (0.97%) | 1497.886 (101.37%) | -34.576 (-2.34%) |
| Antigua and Barbuda | 7.842 | 0.956 (12.19%) | 7.081 (90.3%) | -0.195 (-2.49%) |
| Argentina | 12928 | 4293.72 (33.21%) | 9990.739 (77.28%) | -1356.464 (-10.49%) |
| Armenia | 166.928 | 71.521 (42.85%) | 3.286 (1.97%) | 92.121 (55.19%) |
| Australia | 9037.299 | 5781.175 (63.97%) | 5761.029 (63.75%) | -2504.905 (-27.72%) |

| | | | | |
|---|---|---|---|---|
| Austria | 6014.939 | 2394.459 (39.81%) | 1448.363 (24.08%) | 2172.117 (36.11%) |
| Azerbaijan | 279.587 | 39.16 (14.01%) | 158.543 (56.71%) | 81.884 (29.29%) |
| Bahamas | 52.128 | 15.848 (30.4%) | 29.123 (55.87%) | 7.157 (13.73%) |
| Bahrain | 133.077 | 29.187 (21.93%) | 118.539 (89.08%) | -14.649 (-11.01%) |
| Bangladesh | 12183.63 | 3840.095 (31.52%) | 7487.19 (61.45%) | 856.34 (7.03%) |
| Barbados | 45.642 | 17.054 (37.36%) | 21.468 (47.04%) | 7.12 (15.6%) |
| Belarus | 529.471 | 138.481 (26.15%) | -16.682 (-3.15%) | 407.672 (77%) |
| Belgium | 7766.199 | 4765.772 (61.37%) | 1838.525 (23.67%) | 1161.902 (14.96%) |
| Belize | 25.454 | 1.014 (3.98%) | 18.93 (74.37%) | 5.51 (21.65%) |
| Benin | 576.436 | -28.108 (-4.88%) | 626.733 (108.73%) | -22.189 (-3.85%) |
| Bermuda | 12.997 | 37.797 (290.81%) | 11.8 (90.79%) | -36.6 (-281.6%) |
| Bhutan | 55.149 | 20.69 (37.52%) | 22.703 (41.17%) | 11.756 (21.32%) |
| Bolivia (Plurinational State of) | 1023.406 | 204.337 (19.97%) | 868.305 (84.84%) | -49.236 (-4.81%) |
| Bosnia and Herzegovina | 809.099 | 676.075 (83.56%) | -201.515 (-24.91%) | 334.539 (41.35%) |
| Botswana | 143.742 | 14.172 (9.86%) | 135.334 (94.15%) | -5.764 (-4.01%) |
| Brazil | 25763.23 | 18365.766 (71.29%) | 30854.822 (119.76%) | -23457.358 (-91.05%) |
| Brunei Darussalam | 82.187 | 19.966 (24.29%) | 43.18 (52.54%) | 19.041 (23.17%) |
| Bulgaria | 1126.967 | 355.838 (31.57%) | -199.756 (-17.73%) | 970.885 (86.15%) |
| Burkina Faso | 1216.324 | -143.386 (-11.79%) | 1172.849 (96.43%) | 186.861 (15.36%) |
| Burundi | 176.142 | -70.531 (-40.04%) | 573.301 (325.48%) | -326.628 (-185.43%) |
| Cabo Verde | 52.496 | 1.858 (3.54%) | 35.58 (67.78%) | 15.058 (28.68%) |
| Cambodia | 323.012 | 84.538 | 268.404 | -29.93 |

| | | | | |
|---|---|---|---|---|
| | | (26.17%) | (83.09%) | (-9.27%) |
| Cameroon | 1887.17 | -112.372 (-5.95%) | 1755.164 (93.01%) | 244.378 (12.95%) |
| Canada | 15297.22 | 8476.505 (55.41%) | 6363.328 (41.6%) | 457.385 (2.99%) |
| Central African Republic | 190.42 | -21.467 (-11.27%) | 264.266 (138.78%) | -52.379 (-27.51%) |
| Chad | 584.377 | -94.472 (-16.17%) | 624.85 (106.93%) | 53.999 (9.24%) |
| Chile | 3141.258 | 1807.292 (57.53%) | 2122.188 (67.56%) | -788.222 (-25.09%) |
| China | 35465.78 | 23798.959 (67.1%) | 20043.022 (56.51%) | -8376.205 (-23.62%) |
| Colombia | 7334.189 | 4184.039 (57.05%) | 5956.851 (81.22%) | -2806.701 (-38.27%) |
| Comoros | 32.766 | 7.437 (22.7%) | 37.746 (115.2%) | -12.417 (-37.9%) |
| Congo | 274.318 | 14.706 (5.36%) | 358.077 (130.53%) | -98.465 (-35.89%) |
| Cook Islands | 0.476 | 0.654 (137.39%) | 0.203 (42.65%) | -0.381 (-80.04%) |
| Costa Rica | 1049.748 | 303.595 (28.92%) | 535.663 (51.03%) | 210.49 (20.05%) |
| Croatia | 4121.2 | 1129.037 (27.4%) | -187.345 (-4.55%) | 3179.508 (77.15%) |
| Cuba | 1736.763 | 1122.355 (64.62%) | 443.703 (25.55%) | 170.705 (9.83%) |
| Cyprus | 825.169 | 513.157 (62.19%) | 838.236 (101.58%) | -526.224 (-63.77%) |
| Czechia | 8997.236 | 1556.142 (17.3%) | 468.335 (5.21%) | 6972.759 (77.5%) |
| Cote d'Ivoire | 1187.926 | 117.103 (9.86%) | 1160.67 (97.71%) | -89.847 (-7.56%) |
| Democratic People's Republic of Korea | 638.3 | 263.473 (41.28%) | 545.685 (85.49%) | -170.858 (-26.77%) |
| Democratic Republic of the Congo | 3721.039 | -215.321 (-5.79%) | 4218.659 (113.37%) | -282.299 (-7.59%) |
| Denmark | 3651.458 | 1113.558 (30.5%) | 625.464 (17.13%) | 1912.436 (52.37%) |
| Djibouti | 79.372 | 18.692 (23.55%) | 69.498 (87.56%) | -8.818 (-11.11%) |
| Dominica | 5.15 | 1.782 | 1.491 | 1.877 |

| | | | | |
|---|---|---|---|---|
| | | (34.6%) | (28.95%) | (36.45%) |
| Dominican Republic | 987.857 | 254.06<br>(25.72%) | 654.099<br>(66.21%) | 79.698<br>(8.07%) |
| Ecuador | 2218.418 | 383.306<br>(17.28%) | 1214.433<br>(54.74%) | 620.679<br>(27.98%) |
| Egypt | 11538.51 | 641.039<br>(5.56%) | 9696.622<br>(84.04%) | 1200.849<br>(10.41%) |
| El Salvador | 186.863 | 61.208<br>(32.76%) | 120.703<br>(64.59%) | 4.952<br>(2.65%) |
| Equatorial Guinea | 58.932 | -24.618<br>(-41.77%) | 93.736<br>(159.06%) | -10.186<br>(-17.28%) |
| Eritrea | 302.277 | 23.159<br>(7.66%) | 339.862<br>(112.43%) | -60.744<br>(-20.1%) |
| Estonia | 1110.393 | 269.743<br>(24.29%) | -73.493<br>(-6.62%) | 914.143<br>(82.33%) |
| Eswatini | 53.431 | 4.907<br>(9.18%) | 39.767<br>(74.43%) | 8.757<br>(16.39%) |
| Ethiopia | 1598.636 | -119.056<br>(-7.45%) | 4180.085<br>(261.48%) | -2462.393<br>(-154.03%) |
| Fiji | 20.065 | 9.85<br>(49.09%) | 17.6<br>(87.71%) | -7.385<br>(-36.81%) |
| Finland | 4552.98 | 2972.975<br>(65.3%) | 915.577<br>(20.11%) | 664.428<br>(14.59%) |
| France | 19435.63 | 30121.013<br>(154.98%) | 11812.801<br>(60.78%) | -22498.186<br>(-115.76%) |
| Gabon | 108.226 | -11.247<br>(-10.39%) | 127.204<br>(117.54%) | -7.731<br>(-7.14%) |
| Gambia | 121.478 | 5.608<br>(4.62%) | 101.175<br>(83.29%) | 14.695<br>(12.1%) |
| Georgia | 630.147 | 162.987<br>(25.86%) | -198.53<br>(-31.51%) | 665.69<br>(105.64%) |
| Germany | 58261.19 | 45339.249<br>(77.82%) | 10593.917<br>(18.18%) | 2328.019<br>(4%) |
| Ghana | 1725.829 | 87.866<br>(5.09%) | 1455.705<br>(84.35%) | 182.258<br>(10.56%) |
| Greece | 7068.38 | 4460.238<br>(63.1%) | 502.13<br>(7.1%) | 2106.012<br>(29.79%) |
| Greenland | 11.607 | 17.93<br>(154.48%) | 2.164<br>(18.64%) | -8.487<br>(-73.12%) |
| Grenada | 11.256 | 0.709<br>(6.3%) | 8.818<br>(78.34%) | 1.729<br>(15.36%) |
| Guam | 42.421 | 15.082<br>(35.55%) | 13.248<br>(31.23%) | 14.091<br>(33.22%) |

| | | | | |
|---|---|---|---|---|
| Guatemala | 526.751 | 49.841 (9.46%) | 591.749 (112.34%) | -114.839 (-21.8%) |
| Guinea | 532.974 | -96.021 (-18.02%) | 535.01 (100.38%) | 93.985 (17.63%) |
| Guinea-Bissau | 88.297 | -7.993 (-9.05%) | 98.571 (111.64%) | -2.281 (-2.58%) |
| Guyana | -9.683 | 74.328 (-767.61%) | 48.217 (-497.96%) | -132.228 (1365.57%) |
| Haiti | 934.858 | -11.1 (-1.19%) | 1165.24 (124.64%) | -219.282 (-23.46%) |
| Honduras | 692.869 | 68.495 (9.89%) | 515.976 (74.47%) | 108.398 (15.64%) |
| Hungary | 7359.69 | 2740.162 (37.23%) | 29.939 (0.41%) | 4589.589 (62.36%) |
| Iceland | 142.608 | 66.699 (46.77%) | 81.651 (57.26%) | -5.742 (-4.03%) |
| India | 87779.18 | 27677.674 (31.53%) | 64428.159 (73.4%) | -4326.65 (-4.93%) |
| Indonesia | 7409.75 | 2206.031 (29.77%) | 4569.242 (61.67%) | 634.477 (8.56%) |
| Iran (Islamic Republic of) | 8891.234 | 2971.157 (33.42%) | 7594.093 (85.41%) | -1674.016 (-18.83%) |
| Iraq | 1691.225 | 54.738 (3.24%) | 1447.112 (85.57%) | 189.375 (11.2%) |
| Ireland | 1214.239 | 595.416 (49.04%) | 997.986 (82.19%) | -379.163 (-31.23%) |
| Israel | 3112.834 | 1161.916 (37.33%) | 2457.335 (78.94%) | -506.417 (-16.27%) |
| Italy | 32967.42 | 15250.622 (46.26%) | 3494.335 (10.6%) | 14222.462 (43.14%) |
| Jamaica | 115.748 | 15.706 (13.57%) | 57.476 (49.66%) | 42.566 (36.77%) |
| Japan | 62383.5 | 89268.056 (143.1%) | 8680.991 (13.92%) | -35565.546 (-57.01%) |
| Jordan | 454.346 | 127.38 (28.04%) | 693.586 (152.66%) | -366.62 (-80.69%) |
| Kazakhstan | 563.28 | 77.747 (13.8%) | 116.745 (20.73%) | 368.788 (65.47%) |
| Kenya | 2430.316 | 20.586 (0.85%) | 2138.875 (88.01%) | 270.855 (11.14%) |
| Kiribati | 6.106 | 0.169 (2.77%) | 5.803 (95.04%) | 0.134 (2.19%) |
| Kuwait | 348.546 | 43.837 (12.58%) | 274.475 (78.75%) | 30.234 (8.67%) |

| | | | | |
|---|---|---|---|---|
| Kyrgyzstan | 60.697 | 0.296 (0.49%) | 94.01 (154.88%) | -33.609 (-55.37%) |
| Lao People's Democratic Republic | 146.436 | 3.811 (2.6%) | 132.184 (90.27%) | 10.441 (7.13%) |
| Latvia | 561.015 | 203.744 (36.32%) | -167.729 (-29.9%) | 525 (93.58%) |
| Lebanon | 687.866 | 158.593 (23.06%) | 642.34 (93.38%) | -113.067 (-16.44%) |
| Lesotho | 96.767 | -9.334 (-9.65%) | 55.924 (57.79%) | 50.177 (51.85%) |
| Liberia | 208.707 | -43.924 (-21.05%) | 260.166 (124.66%) | -7.535 (-3.61%) |
| Libya | 698.274 | 77.921 (11.16%) | 583.694 (83.59%) | 36.659 (5.25%) |
| Lithuania | 773.631 | 219.876 (28.42%) | -120.203 (-15.54%) | 673.958 (87.12%) |
| Luxembourg | 314.739 | 90.782 (28.84%) | 240.668 (76.47%) | -16.711 (-5.31%) |
| Madagascar | 1433.507 | -194.304 (-13.55%) | 2252.413 (157.13%) | -624.602 (-43.57%) |
| Malawi | 668.147 | -38.231 (-5.72%) | 711.244 (106.45%) | -4.866 (-0.73%) |
| Malaysia | 1896.744 | 409.647 (21.6%) | 1199.162 (63.22%) | 287.935 (15.18%) |
| Maldives | 26.788 | 3.553 (13.26%) | 27.027 (100.89%) | -3.792 (-14.16%) |
| Mali | 765.693 | -117.114 (-15.3%) | 895.634 (116.97%) | -12.827 (-1.68%) |
| Malta | 147.824 | 98.952 (66.94%) | 49.955 (33.79%) | -1.083 (-0.73%) |
| Marshall Islands | 3.555 | 0.346 (9.73%) | 3.432 (96.54%) | -0.223 (-6.27%) |
| Mauritania | 97.719 | 1.493 (1.53%) | 178.533 (182.7%) | -82.307 (-84.23%) |
| Mauritius | 91.615 | 38.628 (42.16%) | 62.278 (67.98%) | -9.291 (-10.14%) |
| Mexico | 12699.99 | 4023.195 (31.68%) | 6822.401 (53.72%) | 1854.392 (14.6%) |
| Micronesia (Federated States of) | 3.999 | 0.8 (20.01%) | 3.465 (86.65%) | -0.266 (-6.65%) |
| Monaco | 5.486 | 1.487 (27.11%) | 2.735 (49.85%) | 1.264 (23.04%) |

| | | | | |
|---|---|---|---|---|
| Mongolia | 119.533 | 23.308 (19.5%) | 85.053 (71.15%) | 11.172 (9.35%) |
| Montenegro | 71.804 | 27.009 (37.61%) | 7.773 (10.83%) | 37.022 (51.56%) |
| Morocco | 3946.038 | 1046.524 (26.52%) | 2514.958 (63.73%) | 384.556 (9.75%) |
| Mozambique | 1524.624 | -263.773 (-17.3%) | 1311.518 (86.02%) | 476.879 (31.28%) |
| Myanmar | 890.064 | 414.119 (46.53%) | 935.787 (105.14%) | -459.842 (-51.66%) |
| Namibia | 118.677 | 9.841 (8.29%) | 105.18 (88.63%) | 3.656 (3.08%) |
| Nauru | 0.101 | -0.056 (-55.45%) | 0.221 (218.81%) | -0.064 (-63.37%) |
| Nepal | 2134.503 | 470.835 (22.06%) | 1270.001 (59.5%) | 393.667 (18.44%) |
| Netherlands | 10209.86 | 6666.776 (65.3%) | 2784.294 (27.27%) | 758.791 (7.43%) |
| New Zealand | 2783.871 | 1626.23 (58.42%) | 1182.254 (42.47%) | -24.613 (-0.88%) |
| Nicaragua | 224.733 | 53.603 (23.85%) | 222.33 (98.93%) | -51.2 (-22.78%) |
| Niger | 756.768 | -17.434 (-2.3%) | 845.209 (111.69%) | -71.007 (-9.38%) |
| Nigeria | 4360.301 | -829.092 (-19.01%) | 9519.676 (218.33%) | -4330.283 (-99.31%) |
| Niue | -0.128 | 0.01 (-7.81%) | -0.071 (55.47%) | -0.067 (52.34%) |
| North Macedonia | 292.834 | 89.403 (30.53%) | 55.958 (19.11%) | 147.473 (50.36%) |
| Northern Mariana Islands | 10.294 | 4.706 (45.72%) | 1.096 (10.65%) | 4.492 (43.64%) |
| Norway | 1550.675 | 687.46 (44.33%) | 1532.62 (98.84%) | -669.405 (-43.17%) |
| Oman | 402.569 | -33.468 (-8.31%) | 509.763 (126.63%) | -73.726 (-18.31%) |
| Pakistan | 11660.71 | -1797.358 (-15.41%) | 10410.433 (89.28%) | 3047.636 (26.14%) |
| Palau | 0.649 | 0.286 (44.07%) | 0.337 (51.93%) | 0.026 (4.01%) |
| Palestine | 144.418 | 1.472 (1.02%) | 154.755 (107.16%) | -11.809 (-8.18%) |
| Panama | 143.477 | 103.191 (71.92%) | 291.277 (203.01%) | -250.991 (-174.93%) |

| | | | | |
|---|---|---|---|---|
| Papua New Guinea | 532.032 | 2.035<br>(0.38%) | 491.595<br>(92.4%) | 38.402<br>(7.22%) |
| Paraguay | 1830.163 | 207.94<br>(11.36%) | 914.343<br>(49.96%) | 707.88<br>(38.68%) |
| Peru | 717.872 | 489.9<br>(68.24%) | 1367.222<br>(190.45%) | -1139.25<br>(-158.7%) |
| Philippines | 2859.789 | 384.587<br>(13.45%) | 1285.467<br>(44.95%) | 1189.735<br>(41.6%) |
| Poland | 23725.74 | 4381.143<br>(18.47%) | 1626.527<br>(6.86%) | 17718.066<br>(74.68%) |
| Portugal | 8264.536 | 3226.473<br>(39.04%) | 960.52<br>(11.62%) | 4077.543<br>(49.34%) |
| Puerto Rico | 678.241 | 615.396<br>(90.73%) | 172.666<br>(25.46%) | -109.821<br>(-16.19%) |
| Qatar | 132.151 | 4.5<br>(3.41%) | 198.184<br>(149.97%) | -70.533<br>(-53.37%) |
| Republic of Korea | 8268.765 | 4916.616<br>(59.46%) | 2118.416<br>(25.62%) | 1233.733<br>(14.92%) |
| Republic of Moldova | 191.413 | 74.167<br>(38.75%) | -9.279<br>(-4.85%) | 126.525<br>(66.1%) |
| Romania | 5402.012 | 2332.243<br>(43.17%) | -608.702<br>(-11.27%) | 3678.471<br>(68.09%) |
| Russian Federation | 25491.82 | 4613.278<br>(18.1%) | 641.582<br>(2.52%) | 20236.955<br>(79.39%) |
| Rwanda | 321.075 | 21.374<br>(6.66%) | 745.546<br>(232.2%) | -445.845<br>(-138.86%) |
| Saint Kitts and Nevis | 3.968 | -0.05<br>(-1.26%) | 6.372<br>(160.58%) | -2.354<br>(-59.32%) |
| Saint Lucia | 20.989 | 9.673<br>(46.09%) | 15.406<br>(73.4%) | -4.09<br>(-19.49%) |
| Saint Vincent and the Grenadines | 3.219 | 8.436<br>(262.07%) | 7.421<br>(230.54%) | -12.638<br>(-392.61%) |
| Samoa | 6.988 | 2.163<br>(30.95%) | 8.59<br>(122.93%) | -3.765<br>(-53.88%) |
| San Marino | 15.339 | 6.453<br>(42.07%) | 5.293<br>(34.51%) | 3.593<br>(23.42%) |
| Sao Tome and Principe | 11.913 | -0.59<br>(-4.95%) | 8.482<br>(71.2%) | 4.021<br>(33.75%) |
| Saudi Arabia | 2402.887 | 112.379<br>(4.68%) | 1877.676<br>(78.14%) | 412.832<br>(17.18%) |
| Senegal | 658.863 | 29.568<br>(4.49%) | 621.765<br>(94.37%) | 7.53<br>(1.14%) |
| Serbia | 5153.818 | 1971.615<br>(38.26%) | -2.641<br>(-0.05%) | 3184.844<br>(61.8%) |

| | | | | |
|---|---|---|---|---|
| Seychelles | 7.632 | 1.512 (19.81%) | 5.43 (71.15%) | 0.69 (9.04%) |
| Sierra Leone | 372.544 | -56.103 (-15.06%) | 376.869 (101.16%) | 51.778 (13.9%) |
| Singapore | 488.765 | 309.096 (63.24%) | 437.974 (89.61%) | -258.305 (-52.85%) |
| Slovakia | 2251.404 | 366.666 (16.29%) | 219.891 (9.77%) | 1664.847 (73.95%) |
| Slovenia | 3475.605 | 1329.861 (38.26%) | 304.284 (8.75%) | 1841.46 (52.98%) |
| Solomon Islands | 42.133 | 2.683 (6.37%) | 41.443 (98.36%) | -1.993 (-4.73%) |
| Somalia | 619.362 | -81.841 (-13.21%) | 1025.592 (165.59%) | -324.389 (-52.37%) |
| South Africa | 2876.814 | 883.997 (30.73%) | 3274.827 (113.84%) | -1282.01 (-44.56%) |
| South Sudan | 39.794 | 6.291 (15.81%) | 210.759 (529.63%) | -177.256 (-445.43%) |
| Spain | 28431.12 | 16769.687 (58.98%) | 9856.844 (34.67%) | 1804.592 (6.35%) |
| Sri Lanka | 1183.799 | 1149.232 (97.08%) | 1076.141 (90.91%) | -1041.574 (-87.99%) |
| Sudan | 2581.238 | -173.675 (-6.73%) | 2815.404 (109.07%) | -60.491 (-2.34%) |
| Suriname | 61.205 | 17.166 (28.05%) | 38.616 (63.09%) | 5.423 (8.86%) |
| Sweden | 3684.033 | 2130.522 (57.83%) | 1949.826 (52.93%) | -396.315 (-10.76%) |
| Switzerland | 2863.019 | 1958.267 (68.4%) | 1700.703 (59.4%) | -795.951 (-27.8%) |
| Syrian Arab Republic | 360.665 | 1267.341 (351.39%) | 2049.023 (568.12%) | -2955.699 (-819.51%) |
| Taiwan (Province of China) | 3725.006 | 1926.297 (51.71%) | 930.614 (24.98%) | 868.095 (23.3%) |
| Tajikistan | 169.57 | -6.645 (-3.92%) | 143.197 (84.45%) | 33.018 (19.47%) |
| Thailand | 4360.291 | 2243.186 (51.45%) | 1654.239 (37.94%) | 462.866 (10.62%) |
| Timor-Leste | 29.875 | 8.239 (27.58%) | 14.199 (47.53%) | 7.437 (24.89%) |
| Togo | 515.028 | 64.865 (12.59%) | 411.11 (79.82%) | 39.053 (7.58%) |
| Tokelau | -0.039 | -0.01 (25.64%) | -0.01 (25.64%) | -0.019 (48.72%) |

| | | | | |
|---|---|---|---|---|
| Tonga | 3.721 | 1.916 (51.49%) | 1.791 (48.13%) | 0.014 (0.38%) |
| Trinidad and Tobago | 106.89 | 56.552 (52.91%) | 62.641 (58.6%) | -12.303 (-11.51%) |
| Tunisia | 1424.678 | 520.713 (36.55%) | 930.332 (65.3%) | -26.367 (-1.85%) |
| Turkey | 5474.926 | 4493.526 (82.07%) | 7726.079 (141.12%) | -6744.679 (-123.19%) |
| Turkmenistan | 141.425 | 22.58 (15.97%) | 64.585 (45.67%) | 54.26 (38.37%) |
| Tuvalu | 0.513 | 0.128 (24.95%) | 0.596 (116.18%) | -0.211 (-41.13%) |
| Uganda | 1471.112 | -159.751 (-10.86%) | 1615.385 (109.81%) | 15.478 (1.05%) |
| Ukraine | 3246.152 | 608.453 (18.74%) | -436.951 (-13.46%) | 3074.65 (94.72%) |
| United Arab Emirates | 1907.256 | 263.067 (13.79%) | 1624.764 (85.19%) | 19.425 (1.02%) |
| United Kingdom | 21264.79 | 10778.611 (50.69%) | 9894.775 (46.53%) | 591.399 (2.78%) |
| United Republic of Tanzania | 2389.637 | -26.763 (-1.12%) | 3082.729 (129%) | -666.329 (-27.88%) |
| United States of America | 92794.01 | 61554.256 (66.33%) | 71798.223 (77.37%) | -40558.471 (-43.71%) |
| United States Virgin Islands | 37.247 | 27.539 (73.94%) | 6.634 (17.81%) | 3.074 (8.25%) |
| Uruguay | 2181.516 | 777.538 (35.64%) | 509.935 (23.38%) | 894.043 (40.98%) |
| Uzbekistan | 1649.588 | -40.143 (-2.43%) | 604.672 (36.66%) | 1085.059 (65.78%) |
| Vanuatu | 23.919 | 2.171 (9.08%) | 18.961 (79.27%) | 2.787 (11.65%) |
| Venezuela (Bolivarian Republic of) | 3886.955 | 1592.637 (40.97%) | 2325.503 (59.83%) | -31.185 (-0.8%) |
| Viet Nam | 3002.644 | 951.604 (31.69%) | 2376.748 (79.16%) | -325.708 (-10.85%) |
| Yemen | 2364.37 | -16.566 (-0.7%) | 2300.93 (97.32%) | 80.006 (3.38%) |
| Zambia | 1187.462 | -36.201 (-3.05%) | 913.987 (76.97%) | 309.676 (26.08%) |
| Zimbabwe | 691.344 | 42.029 (6.08%) | 474.854 (68.69%) | 174.461 (25.24%) |

a.Change in DALYs number between year 2019 and 1990.

b.Change in DALYs number due to change in the age structure.
c.Change in DALYs number due to change in population number.
d.Change in DALYs number due to epidemiologic changes. Epidemiologic changes refer to the DALYs number change when age structure and population hold constant.
DALYs, Disability-Adjusted Life Years; SDI: Socio-demographic index.
