# Supplementary material for: Global Burden and Improvement Gap of Non-Rheumatic Calcific Aortic Valve Disease: 1990–2019 Findings from Global Burden of Disease Study 2019
Source: J Clin Med. 2022 Nov 14;11(22):6733. doi: 10.3390/jcm11226733 (PMC9698619; doi:10.3390/jcm11226733)
Supplement: Supplementary file 1 [file jcm-11-06733-s001.zip › Supplemental Table S6 frontier.pdf]

Table S6. Frontier DALYs and effective difference by country or territory.

	SDI	Age standardized DALYs	Frontier DALYs	Effective difference	Effective difference rank (Age standardized DALYs rank)
Afghanistan	0.343	21.27<br>(10.74 to 33.83)	3.65	17.63	120 (127)
Albania	0.681	11.56<br>(8.39 to 15.37)	2.49	9.07	43 (35)
Algeria	0.652	21.12<br>(13.79 to 28.87)	2.44	18.68	128 (126)
American Samoa	0.712	23.53<br>(18.83 to 28.9)	2.49	21.04	140 (139)
Andorra	0.894	26.72<br>(19.84 to 35.11)	2.48	24.24	151 (151)
Angola	0.47	19.74<br>(15.01 to 26.06)	3.65	16.09	111 (114)
Antigua and Barbuda	0.743	17.78<br>(14.59 to 22.4)	2.49	15.29	100 (95)
Argentina	0.708	57.86<br>(52.02 to 63.25)	2.48	55.38	190 (190)
Armenia	0.689	7.55<br>(6.3 to 8.97)	2.47	5.08	29 (24)
Australia	0.839	41.11<br>(35.26 to 47.46)	2.49	38.63	169 (169)
Austria	0.849	58.84<br>(50.9 to 67.64)	2.44	56.4	191 (191)

Azerbaijan	0.683	4.98<br>(3.94 to 6.28)	2.49	2.49	10 (6)
Bahrain	0.751	21.57<br>(17 to 27.14)	2.49	19.09	131 (130)
Bangladesh	0.483	14.78<br>(9.74 to 21.06)	3.65	11.13	59 (61)
Barbados	0.742	23.49<br>(19.23 to 28.46)	2.49	21	139 (138)
Belarus	0.745	6.11<br>(4.69 to 8)	2.49	3.62	19 (15)
Belgium	0.851	59.52<br>(49.61 to 66.56)	2.49	57.03	192 (192)
Belize	0.603	11.36<br>(9.27 to 14.55)	2.48	8.88	40 (34)
Benin	0.352	14.81<br>(8.5 to 23.48)	3.66	11.16	61 (64)
Bermuda	0.813	69.18<br>(57.44 to 84.51)	2.48	66.7	200 (200)
Bhutan	0.455	15.37<br>(10.31 to 21.37)	3.63	11.74	67 (69)
Bolivia	0.566	18.54<br>(12.93 to 26.05)	2.47	16.07	109 (104)
Bosnia and Herzegovina	0.718	24.66<br>(19.24 to 31.55)	2.48	22.18	146 (145)
Botswana	0.634	16.7<br>(11.24 to 25.55)	2.48	14.22	89 (84)

Brazil	0.64	29.2<br>(26.27 to 33.44)	2.49	26.71	160 (160)
Brunei	0.823	49.33<br>(40.45 to 57.24)	2.49	46.84	182 (182)
Bulgaria	0.764	14.32<br>(10.91 to 18.36)	2.49	11.83	69 (59)
Burkina Faso	0.257	17.72<br>(10.03 to 27.6)	11.88	5.84	31 (94)
Burundi	0.284	15.33<br>(10.27 to 22.05)	4.62	10.71	53 (67)
Cote d'Ivoire	0.408	13.91<br>(8.24 to 21.67)	3.65	10.26	52 (56)
Cambodia	0.469	4.54<br>(3.3 to 7.07)	3.65	0.89	2 (4)
Cameroon	0.49	17.17<br>(10.31 to 27.69)	3.65	13.52	81 (90)
Canada	0.873	37.11<br>(32.16 to 40.74)	2.47	34.64	164 (164)
Cape Verde	0.525	16.76<br>(13.23 to 21.63)	2.48	14.28	91 (85)
Central African Republic	0.274	20.84<br>(11.36 to 32.88)	4.97	15.87	107 (122)
Chad	0.238	13.62<br>(7.54 to 22.27)	11.87	1.76	5 (52)
Chile	0.759	27.65<br>(24.67 to 31.54)	2.49	25.16	156 (156)

China	0.686	4.09<br>(3.32 to 4.93)	2.49	1.61	4 (1)
Colombia	0.633	27.75<br>(19.88 to 38.91)	2.49	25.26	157 (157)
Comoros	0.455	15.42<br>(11.19 to 20.97)	3.66	11.76	68 (70)
Congo (Brazzaville)	0.568	21.02<br>(14.99 to 29.69)	2.49	18.53	127 (124)
Cook Islands	0.764	9.04<br>(7.13 to 11.25)	2.49	6.55	34 (29)
Costa Rica	0.68	28.45<br>(21.73 to 36.52)	2.49	25.96	158 (158)
Croatia	0.794	52.25<br>(38.1 to 66.05)	2.49	49.76	185 (185)
Cuba	0.668	23.68<br>(17.91 to 31.98)	2.49	21.19	142 (141)
Cyprus	0.841	96.09<br>(82.4 to 111.43)	2.48	93.61	204 (204)
Denmark	0.89	54.88<br>(47.22 to 61.42)	2.49	52.39	188 (188)
Djibouti	0.459	18.14<br>(12.25 to 26.66)	3.65	14.49	94 (101)
Dominica	0.729	21.85<br>(16.71 to 28.27)	2.49	19.36	133 (133)
Dominican Republic	0.592	17.96<br>(13.45 to 23.65)	2.48	15.47	104 (99)

DR Congo	0.382	18.35 (11.78 to 27.38)	3.65	14.7	96 (102)
Ecuador	0.64	19.66 (15.01 to 25.26)	2.49	17.17	117 (113)
Egypt	0.658	27.43 (19.63 to 38.9)	2.49	24.95	155 (155)
El Salvador	0.573	7.2 (5.33 to 9.48)	2.48	4.72	24 (20)
Equatorial Guinea	0.685	19.96 (12.78 to 29.04)	2.49	17.47	118 (116)
Eritrea	0.396	17.92 (11.75 to 26.67)	3.65	14.27	90 (98)
Estonia	0.835	46.34 (34.76 to 60.71)	2.49	43.85	176 (176)
eSwatini	0.577	16.07 (10.25 to 24.76)	2.49	13.58	83 (76)
Ethiopia	0.343	12.56 (9.15 to 17.29)	3.67	8.9	41 (43)
Federated States of Micronesia	0.58	24.43 (14.18 to 38.62)	2.49	21.94	145 (144)
Fiji	0.664	9.35 (7.33 to 11.82)	2.48	6.87	35 (30)
Finland	0.856	66.09 (57.31 to 75.68)	2.49	63.6	198 (198)
France	0.834	47.76 (40.45 to 53.44)	2.48	45.28	180 (180)

Gabon	0.656	22.71<br>(17.19 to 29.54)	2.47	20.24	134 (134)
Georgia	0.702	14.79<br>(10.79 to 22)	2.49	12.31	73 (62.5)
Georgia	0.841	14.79<br>(10.79 to 22)	2.49	12.31	72 (62.5)
Germany	0.898	67.99<br>(59.54 to 75.86)	2.49	65.5	199 (199)
Ghana	0.557	13.62<br>(8.44 to 21.87)	2.49	11.13	60 (53)
Greece	0.794	45.23<br>(39.85 to 52.25)	2.47	42.75	175 (175)
Greenland	0.761	60.2<br>(37.11 to 84.43)	2.49	57.72	194 (194)
Grenada	0.669	24.67<br>(20.41 to 32.06)	2.47	22.2	147 (146)
Guam	0.813	40.66<br>(33.21 to 49)	2.49	38.17	168 (168)
Guatemala	0.526	7.07<br>(5.44 to 9.12)	2.49	4.58	23 (19)
Guinea	0.325	15.5<br>(8.92 to 24.68)	3.66	11.84	70 (71)
Guinea-Bissau	0.355	18.64<br>(9.28 to 32.41)	3.66	14.99	98 (106)
Guyana	0.618	46.48<br>(35.06 to 61.5)	2.48	44	177 (177)

Haiti	0.432	25.51<br>(14.35 to 40.74)	3.65	21.86	144 (149)
Honduras	0.496	15.9<br>(12.49 to 20.31)	2.53	13.37	79 (75)
Hungary	0.791	75.23<br>(60.38 to 89.89)	2.49	72.74	202 (202)
Iceland	0.869	49.42<br>(42.05 to 55.92)	2.47	46.95	183 (183)
India	0.566	13.33<br>(9.88 to 17.6)	2.48	10.85	56 (49)
Indonesia	0.66	6.11<br>(4.13 to 8.51)	2.49	3.62	18 (14)
Iran	0.67	20.63<br>(18.56 to 23.89)	2.49	18.14	124 (120)
Iraq	0.671	8.52<br>(5.73 to 11.35)	2.48	6.04	33 (28)
Ireland	0.867	42.15<br>(37.07 to 47.64)	2.48	39.67	170 (170)
Israel	0.803	43.78<br>(37.54 to 49.17)	2.49	41.29	173 (173)
Italy	0.801	34.69<br>(28.94 to 39.17)	2.49	32.21	163 (163)
Jamaica	0.684	7.42<br>(5.72 to 9.82)	2.49	4.93	26 (22)
Japan	0.87	26.55<br>(21.26 to 30.4)	2.49	24.06	150 (150)

Jordan	0.731	10.1<br>(8.25 to 12.37)	2.49	7.61	37 (32)
Kazakhstan	0.723	5.67<br>(4.59 to 7.3)	2.48	3.19	16 (12)
Kenya	0.508	16.1<br>(12.5 to 20.78)	2.51	13.59	84 (77)
Kiribati	0.527	16.95<br>(12.01 to 26.39)	2.49	14.47	93 (88)
Kuwait	0.851	17.9<br>(14.75 to 21.25)	2.49	15.41	102 (97)
Kyrgyzstan	0.596	4.5<br>(3.66 to 5.47)	2.49	2.02	8 (3)
Laos	0.49	5.49<br>(3.97 to 8.06)	3.65	1.84	7 (10)
Latvia	0.82	23.6<br>(18.84 to 29.72)	2.49	21.11	141 (140)
Lebanon	0.708	26.75<br>(16.79 to 40.25)	2.48	24.27	152 (152)
Lesotho	0.507	17.82<br>(11.05 to 28.49)	2.53	15.3	101 (96)
Liberia	0.37	13.21<br>(7.72 to 20.93)	3.67	9.54	49 (47)
Libya	0.709	18.54<br>(11.25 to 26.54)	2.49	16.05	108 (103)
Lithuania	0.843	20.01<br>(15.63 to 24.93)	2.48	17.52	119 (117)

Luxembourg	0.895	60.92<br>(51.18 to 71.65)	2.47	58.45	195 (195)
Madagascar	0.396	24.22<br>(16.13 to 34.01)	3.65	20.56	135 (143)
Malawi	0.384	16.81<br>(12.25 to 22.75)	3.65	13.16	77 (86)
Malaysia	0.737	10.21<br>(7.91 to 13.16)	2.49	7.72	38 (33)
Maldives	0.562	11.56<br>(9.19 to 14.32)	2.49	9.07	44 (36)
Mali	0.263	12.9<br>(7.29 to 21.99)	11.88	1.02	3 (46)
Malta	0.801	27.01<br>(22.89 to 31.14)	2.49	24.52	153 (153)
Marshall Islands	0.544	23.29<br>(14.36 to 35.29)	2.49	20.8	137 (136)
Mauritania	0.496	11.7<br>(7.96 to 17.46)	2.51	9.19	45 (38)
Mauritius	0.705	15.56<br>(12.28 to 19.71)	2.49	13.07	76 (72)
Mexico	0.649	16.3<br>(13.51 to 19.42)	2.49	13.81	87 (81)
Moldova	0.696	6.88<br>(5.4 to 9.07)	2.49	4.39	20 (16)
Monaco	0.902	16.91<br>(13.26 to 21.17)	2.47	14.44	92 (87)

Mongolia	0.606	7.33<br>(5.14 to 12.01)	2.48	4.85	25 (21)
Montenegro	0.791	12.62<br>(10.17 to 16.09)	2.49	10.13	50 (45)
Morocco	0.548	21.77<br>(15.39 to 29.22)	2.49	19.28	132 (132)
Mozambique	0.307	21.08<br>(14.55 to 28.62)	4.47	16.61	114 (125)
Myanmar	0.521	5.35<br>(4.04 to 7.69)	2.45	2.9	13 (9)
Namibia	0.612	15.65<br>(11.57 to 21.76)	2.48	13.17	78 (73)
Nauru	0.618	27.41<br>(17.24 to 40.73)	2.49	24.92	154 (154)
Nepal	0.422	15.32<br>(10.78 to 20.95)	3.65	11.67	65 (66)
Netherlands	0.883	60<br>(49.94 to 68.18)	2.49	57.51	193 (193)
New Zealand	0.84	63.13<br>(54.19 to 70.31)	2.49	60.64	196 (196)
Nicaragua	0.517	8.42<br>(6.65 to 10.93)	2.49	5.94	32 (27)
Niger	0.162	12.56<br>(7.08 to 20.52)	12.33	0.23	1 (42)
Nigeria	0.515	11.68<br>(7.9 to 16.99)	2.49	9.19	46 (37)

Niue	0.711	18.93<br>(14.1 to 25.38)	2.49	16.45	113 (108)
North Korea	0.558	5.49<br>(3.96 to 7.86)	2.48	3.01	15 (11)
North Macedonia	0.744	15.33<br>(12.18 to 19.04)	2.47	12.86	75 (68)
Northern Mariana Islands	0.771	56.46<br>(45.35 to 69.14)	2.49	53.97	189 (189)
Norway	0.913	64.27<br>(53.86 to 71.4)	2.49	61.78	197 (197)
Oman	0.783	28.65<br>(19.46 to 39.02)	2.49	26.16	159 (159)
Pakistan	0.449	17.22<br>(11 to 23.67)	3.65	13.57	82 (92)
Palau	0.738	6.99<br>(5.39 to 9.03)	2.44	4.55	22 (18)
Palestine	0.588	7.51<br>(6.13 to 9.52)	2.49	5.03	27 (23)
Panama	0.686	11.89<br>(8.99 to 15.76)	2.48	9.41	47 (39)
Papua New Guinea	0.394	14.53<br>(8.56 to 22.46)	3.65	10.89	57 (60)
Paraguay	0.638	42.55<br>(31.44 to 55.98)	2.45	40.1	171 (171)
Peru	0.648	7.56<br>(5.5 to 10.22)	2.49	5.07	28 (25)

Philippines	0.623	4.31<br>(3.48 to 5.27)	2.49	1.82	6 (2)
Poland	0.802	37.7<br>(28.53 to 47.55)	2.49	35.21	165 (165)
Portugal	0.743	44.28<br>(38.56 to 49.42)	2.49	41.79	174 (174)
Puerto Rico	0.814	23.79<br>(18.72 to 29.71)	2.49	21.29	143 (142)
Qatar	0.83	16.32<br>(12.27 to 21.65)	2.49	13.83	88 (82)
Romania	0.76	25.39<br>(19.77 to 33.98)	2.48	22.91	149 (148)
Russia	0.805	16.11<br>(12.86 to 20.07)	2.49	13.62	85 (78)
Rwanda	0.429	18.85<br>(13.36 to 27.14)	3.65	15.2	99 (107)
Saint Kitts and Nevis	0.746	21.01<br>(16.54 to 26.94)	2.49	18.52	126 (123)
Saint Lucia	0.67	19.56<br>(16.12 to 24.09)	2.47	17.09	116 (112)
Saint Vincent and the Grenadines	0.627	23.11<br>(19.13 to 29.03)	2.49	20.62	136 (135)
Samoa	0.641	18.1<br>(13.22 to 24.94)	2.48	15.62	106 (100)
San Marino	0.884	30.56<br>(20.74 to 43.9)	2.47	28.09	161 (161)

Saudi Arabia	0.805	13.48<br>(10.27 to 17.58)	2.48	11	58 (51)
Senegal	0.389	12.61<br>(8.36 to 18.17)	3.66	8.96	42 (44)
Serbia	0.767	47.37<br>(37.43 to 59.63)	2.46	44.91	179 (179)
Seychelles	0.724	14.18<br>(11.37 to 17.13)	2.47	11.71	66 (58)
Sierra Leone	0.347	13.79<br>(8.21 to 20.97)	3.65	10.14	51 (54)
Singapore	0.861	11.91<br>(9.57 to 14.87)	2.48	9.42	48 (40)
Slovakia	0.812	31.18<br>(23.63 to 39.19)	2.48	28.7	162 (162)
Slovenia	0.84	91.55<br>(70.09 to 118.18)	2.48	89.07	203 (203)
Solomon Islands	0.407	19.92<br>(10.63 to 33.07)	3.66	16.26	112 (115)
Somalia	0.081	16.27<br>(9.58 to 26.03)	13.26	3.01	14 (80)
South Africa	0.678	17.17<br>(14.02 to 19.67)	2.49	14.69	95 (91)
South Korea	0.878	13.25<br>(11.11 to 15.77)	2.49	10.76	54 (48)
South Sudan	0.363	12.01<br>(7.9 to 17.57)	3.65	8.35	39 (41)

Spain	0.767	52.55<br>(45.6 to 59.91)	2.49	50.06	186 (186)
Sri Lanka	0.69	13.83<br>(10.02 to 18.96)	2.48	11.35	62 (55)
Sudan	0.515	20.7<br>(13.64 to 30.94)	2.48	18.22	125 (121)
Suriname	0.636	18.54<br>(14.8 to 23.72)	2.47	16.07	110 (105)
Sweden	0.872	52.66<br>(46.19 to 58.97)	2.47	50.19	187 (187)
Switzerland	0.929	38.09<br>(32.5 to 43.27)	2.49	35.6	167 (167)
Syria	0.619	37.96<br>(26.75 to 53.41)	2.49	35.48	166 (166)
Taiwan (province of China)	0.868	13.33<br>(10.28 to 17.07)	2.49	10.84	55 (50)
Tajikistan	0.539	5.08<br>(4.04 to 6.34)	2.49	2.59	11 (7)
Tanzania	0.423	19.1<br>(13.28 to 27.13)	3.65	15.45	103 (109)
Thailand	0.687	6.95<br>(5.16 to 9.24)	2.49	4.46	21 (17)
The Bahamas	0.796	21.49<br>(17.38 to 27.21)	2.49	19	130 (129)
The Gambia	0.399	15.13<br>(9.58 to 22.97)	3.65	11.47	63 (65)

Timor-Leste	0.514	4.89<br>(3.02 to 7.56)	2.49	2.4	9 (5)
Togo	0.417	15.77<br>(9.22 to 25.57)	3.65	12.12	71 (74)
Tokelau	0.626	17.25<br>(12.81 to 23.9)	2.49	14.77	97 (93)
Tonga	0.636	16.13<br>(11.57 to 21.98)	2.48	13.64	86 (79)
Trinidad and Tobago	0.757	14.09<br>(10.35 to 18.97)	2.49	11.6	64 (57)
Tunisia	0.672	20.38<br>(12.77 to 29.56)	2.49	17.89	122 (119)
Turkey	0.748	19.24<br>(14.48 to 24.5)	2.47	16.77	115 (111)
Turkmenistan	0.67	5.16<br>(4.05 to 6.76)	2.48	2.68	12 (8)
Tuvalu	0.589	20.32<br>(14.39 to 28.59)	2.49	17.83	121 (118)
Uganda	0.404	16.33<br>(12.15 to 22.2)	3.66	12.67	74 (83)
UK	0.847	50.78<br>(45.49 to 55.33)	2.48	48.3	184 (184)
Ukraine	0.736	8.23<br>(6.66 to 10.13)	2.48	5.75	30 (26)
United Arab Emirates	0.88	24.93<br>(13.93 to 37.82)	2.48	22.44	148 (147)

Uruguay	0.697	73.15<br>(65.09 to 81.49)	2.48	70.67	201 (201)
USA	0.859	47.99<br>(42.61 to 52.83)	2.49	45.5	181 (181)
Uzbekistan	0.631	9.37<br>(7.01 to 13.08)	2.48	6.9	36 (31)
Vanuatu	0.485	19.23<br>(12.23 to 28.18)	3.65	15.58	105 (110)
Venezuela	0.607	21.35<br>(15.53 to 29.4)	2.49	18.86	129 (128)
Vietnam	0.617	6.08<br>(4.4 to 8.72)	2.49	3.59	17 (13)
Virgin Islands	0.799	43.12<br>(35.12 to 52.17)	2.46	40.66	172 (172)
Yemen	0.412	21.61<br>(14.46 to 30.96)	3.65	17.97	123 (131)
Zambia	0.505	23.45<br>(17.21 to 31.97)	2.51	20.94	138 (137)
Zimbabwe	0.476	17.03<br>(12.37 to 24.34)	3.65	13.38	80 (89)

DALYs, disability-adjusted life years; SDI, socio-demographic index; UI: uncertainty interval.
