# Supplementary material for: Global Burden and Improvement Gap of Non-Rheumatic Calcific Aortic Valve Disease: 1990–2019 Findings from Global Burden of Disease Study 2019
Source: J Clin Med. 2022 Nov 14;11(22):6733. doi: 10.3390/jcm11226733 (PMC9698619; doi:10.3390/jcm11226733)
Supplement: Supplementary file 1 [file jcm-11-06733-s001.zip › Supplementary Figure legends nrCAVD.pdf]

Supplemental Figure legends:

Supplemental Figure S1. The age-standardized prevalence rate, incidence rate, mortality and disability-adjusted life-years of nrCAVD versus SDI quintile for men and women, 1990–2019. nrCAVD, non-rheumatic calcific aortic valve disease; SDI, socio-demographic index.

Supplemental Figure S2. The ASPR of nrCAVD versus SDI quintile for men and women by region, 1990–2019. The black line, a LOWESS smoother, shows the expected value only on the SDI values of the global regions between 1990 and 2019. ASPR, age-standardized prevalence rate; LOWESS, locally weighted scatterplot smoothing; nrCAVD, non-rheumatic calcific aortic valve disease; SDI, socio-demographic index.

Supplemental Figure S3. Percentage contributions of major risk factors attributed for DALYs of nrCAVD stratified by age and sex. DALYs, disability-adjusted life-years; nrCAVD, non-rheumatic calcific aortic valve disease.

Supplemental Figure S4. Percentage contributions of major risk factors attributed for DALYs of nrCAVD stratified by SDI quintile and age group. DALYs, disability-adjusted life-years; nrCAVD, non-rheumatic calcific aortic valve disease; SDI, socio-demographic index.
