# Supplementary material for: Global Burden and Improvement Gap of Non-Rheumatic Calcific Aortic Valve Disease: 1990–2019 Findings from Global Burden of Disease Study 2019
Source: J Clin Med. 2022 Nov 14;11(22):6733. doi: 10.3390/jcm11226733 (PMC9698619; doi:10.3390/jcm11226733)
Supplement: Supplementary file 1 [file jcm-11-06733-s001.zip › Supplementary methods.pdf]

Supplemental methods

Statistical analysis

Decomposition analysis:

The decomposition analysis is an analytic approach to identify the additive contribution of the effect of the differences in factors in 2 populations (such as population in 1990 and population in 2019) to the difference in their overall value. The decomposition of nrCAVD DALY by the different causes allows the quantification of the contribution of each cause to the overall nrCAVD DALY. We first used the decomposition methodology of Das Gupta [1-3] to decompose nrCAVD DALYs by population age structure, population growth, and epidemiologic changes (DALYs rate). The number of DALYs at each location was obtained from the following formula: $DALY_{ay, py, ey} = \sum_{i=1}^{20}(a_{i,y} * p_y * e_{i,y})$ where $DALY_{ay, py, ey}$ represented DALYs based on the factors of age structure, population, and DALYs rate for specific year y; $a_{i\,y}$ represents the proportion of population for the age category i of the 20 age categories in given year y; p_y represents the total population in given year y; and $e_{i,y}$ represents DALYs rate given age category i in year y. The contribution of each factor to the change in DALYs from 1990 to 2019 was defined by the effect of one factor changing while the other factors were held constant. For example, the effect of age structure was calculated as:

$$[(DALY_{a2019, p1990, e1990} + DALY_{a2019, p2019, e2019})/3 + (DALY_{a2019, p1990, e2019} + DALY_{a2019, p2019, e1990})/6] - [(DALY_{a1990, p2019, e2019} + DALY_{a1990, p1990, e1990})/3 + (DALY_{a1990, p2019, e1990} + DALY_{a1990, p1990, e2019})/6]$$

Frontier analysis

In order to evaluate the relationship between burden of nrCAVD and socio-demographic development, we applied a frontier analysis as a quantitative methodology to identify the lowest potentially achievable age-standardized DALYs rate on the basis of development status as measured by the Socio-demographic Index (SDI).

In this method, data envelopment analysis (DEA) would be used for frontier analysis. DEA is a non-parametric type

of frontier analysis. DEA methods do not require a functional form for the relationship between variables, as SFA does. The frontier is produced from a deterministic algorithm. The figure below shows examples of DEA frontiers using various algorithms: FDH, VRS.

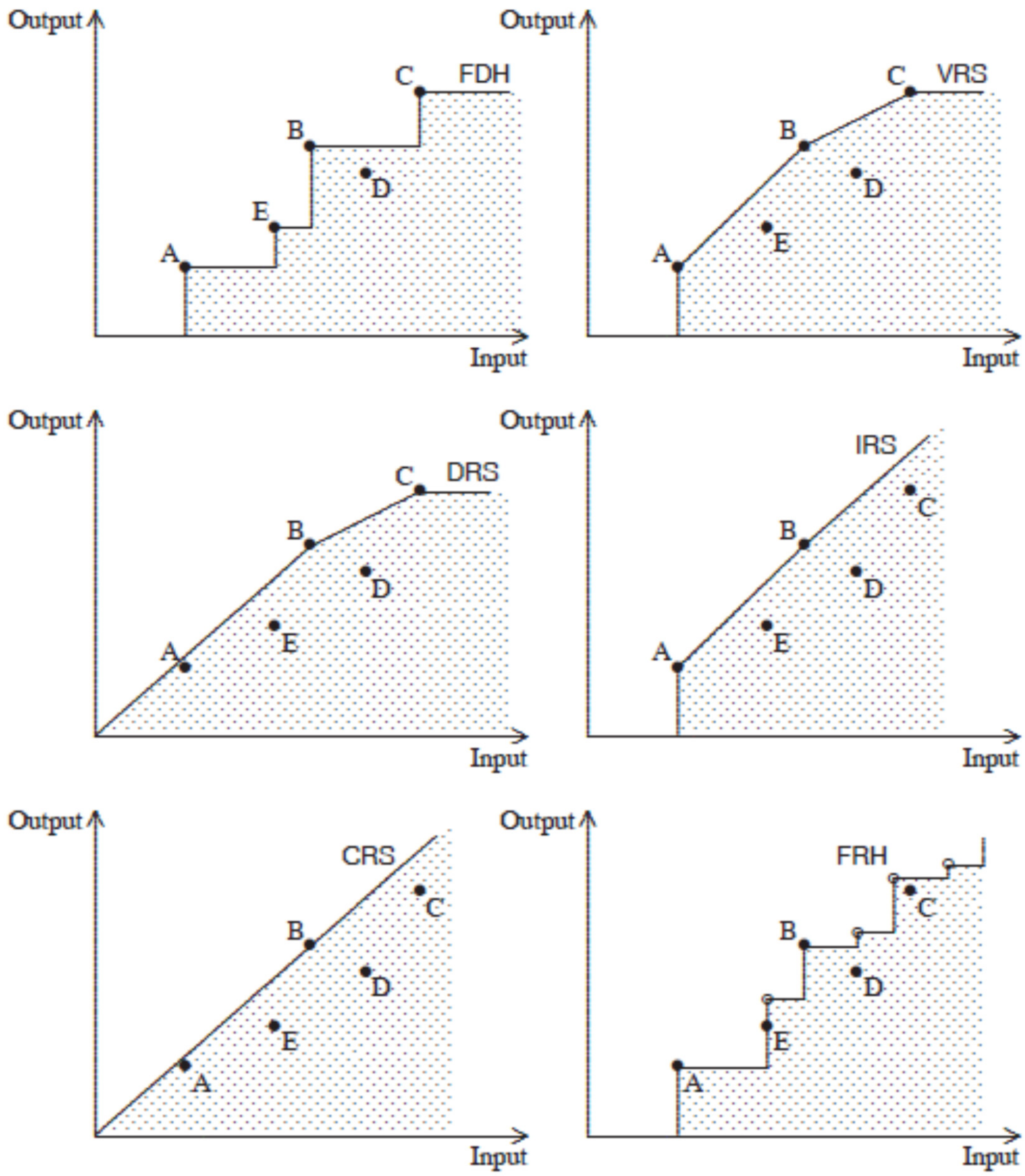

FDH, free disposability hull; VRS, variable returns to scale; DRS, decreasing returns to scale; IRS, increasing returns to scale; CRS, constant returns to scale; FRH, free disposability and replicability hull.

We chose to use the FDH option for this analysis. In contrast to the commonly-used “variable returns to scale” option, FDH relaxes the requirement that the frontier be convex. This is an important property, because the frontier in our data appears convex in some places and concave in others.

In general, the main limitation of DEA methods is that they assume a lack of stochastic variation [4]. That assumption does not hold for this analysis, which is based upon population-level metrics that inherently have a degree of measurement error. To incorporate stochastic variation into the frontier, we used 1,000 bootstrapped samples of the data. Each bootstrap includes a subset of locations produced by randomly sampling with replacement from all countries in the Global Burden of Disease study. This accounts for autocorrelation of locations over time.

In each bootstrapped samples, the following procedure was performed: we firstly remove one data point at a time to generate a DEA and identify if the removed point is a superefficient point (outlier). Then put the data point back, remove the second data point to generate DEA and examine if it is the superefficient point (outlier). In this method, superefficient point is defined as the unit whose number of age-standardized DALY rate less than frontier line at each SDI value calculated after removing the unit. After all the points are examined and removed all superefficient points (outliers), we then generate the frontier using DEA with FDH algorithm. We repeat this step for 1000 iteration bootstrapping and mean nrCAVD DALYs frontier at each SDI value from the bootstrapped samples was computed for each country at each year. Finally, LOESS regression with local polynomial degree of 1 and span of 0.2 was then developed to generate a smoothed frontier [5, 6]. To understand the relationship of age-standardized nrCAVD DALYs rates vis-à-vis the frontier in 2019, we calculated the effective difference (the absolute distance from the frontier) using 2019 SDI and age-standardized nrCAVD DALYs rate data point for each country or territory. Countries or territories with lower DALYs than the frontiers were assigned a zero distance.

References:

1. Gupta P. Standardization and decomposition of rates: a users's manual. U.S. bureau of the census, current population reports 1993:123-186.

2. Das Gupta P. Standardization and decomposition of rates from cross-classified data. Genus 1994;50:171-96.

3. Chevan A, Sutherland M. Revisiting Das Gupta: refinement and extension of standardization and decomposition.

Demography 2009;46:429-49.

4. Hwang S-N, Lee H-S, Zhu J. Handbook of Operations Analytics Using Data Envelopment Analysis.Springer, 2016.

5. Access GBDH, Quality Collaborators. Electronic address cue, Access GBDH, et al. Healthcare Access and Quality Index based on mortality from causes amenable to personal health care in 195 countries and territories, 1990-2015: a novel analysis from the Global Burden of Disease Study 2015. Lancet 2017.

6. Xie Y, Bowe B, Xian H, et al. Rate of Kidney Function Decline and Risk of Hospitalizations in Stage 3A CKD. Clinical journal of the American Society of Nephrology: CJASN 2015; 10: 1946-1955
