# Supplementary figures and images for: Global Burden and Improvement Gap of Non-Rheumatic Calcific Aortic Valve Disease: 1990–2019 Findings from Global Burden of Disease Study 2019
Source: J Clin Med. 2022 Nov 14;11(22):6733. doi: 10.3390/jcm11226733 (PMC9698619; doi:10.3390/jcm11226733)

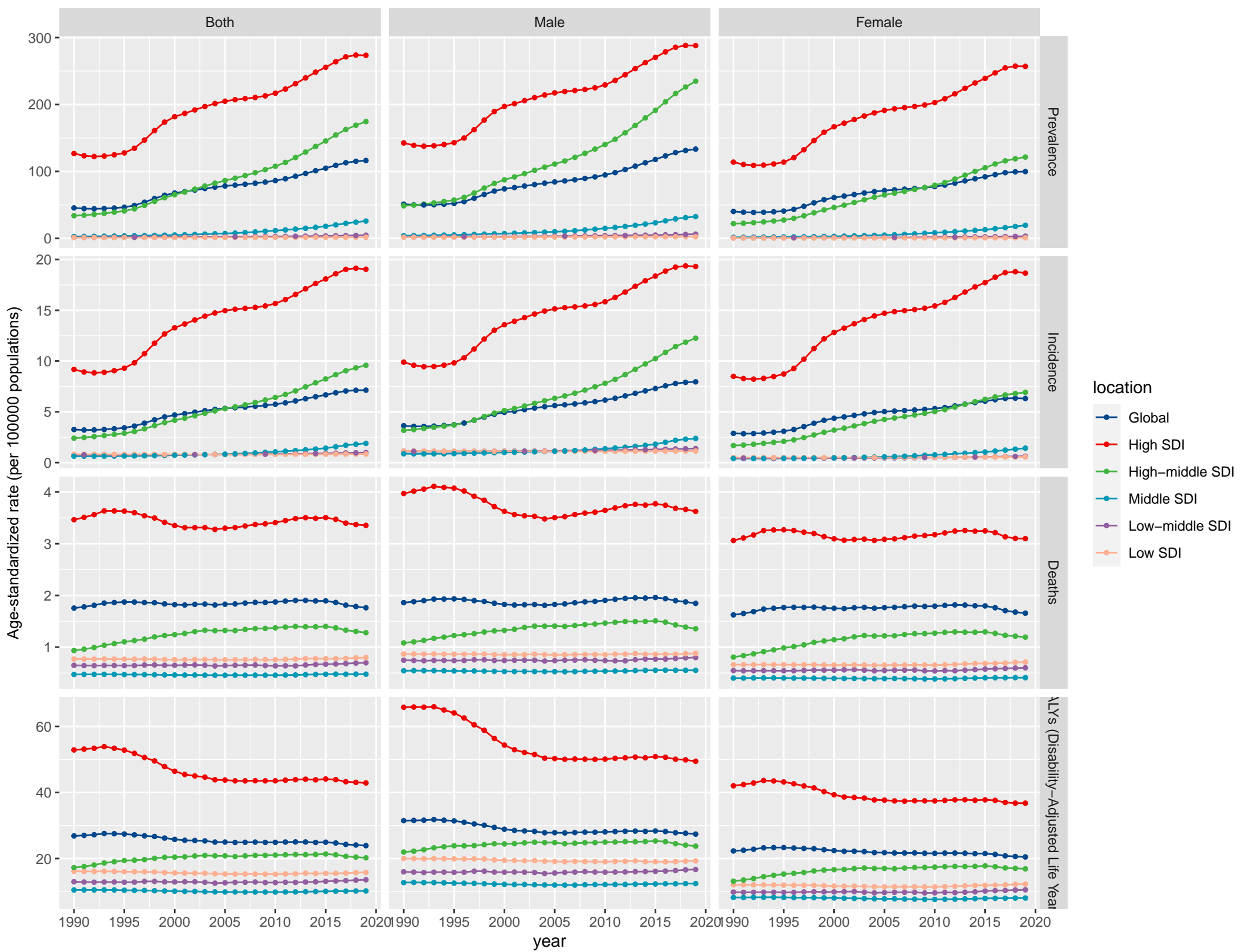

Supplement: Supplementary file 1 [file jcm-11-06733-s001.zip › Figure S1.pdf]

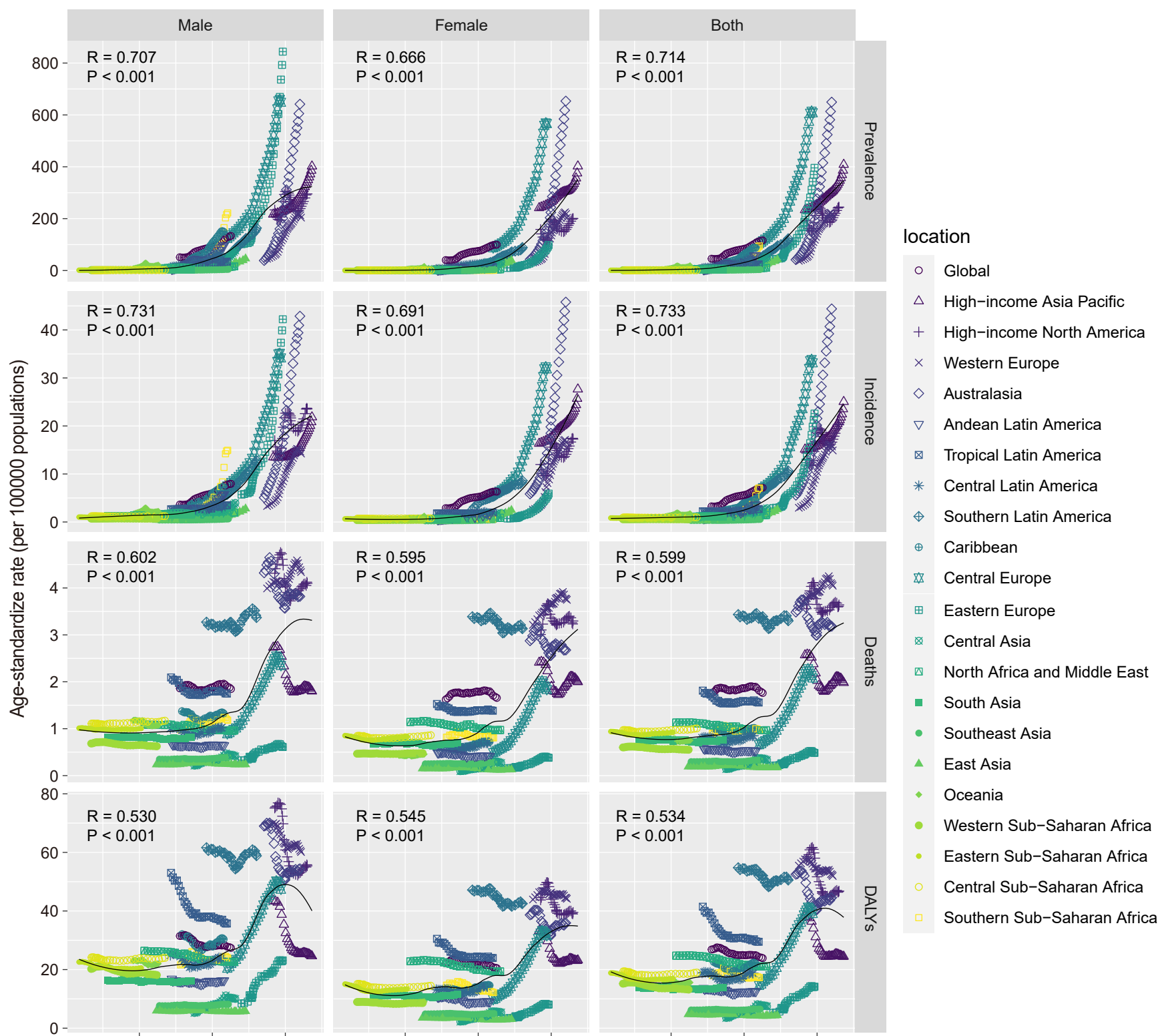

Supplement: Supplementary file 1 [file jcm-11-06733-s001.zip › Figure S2.pdf]

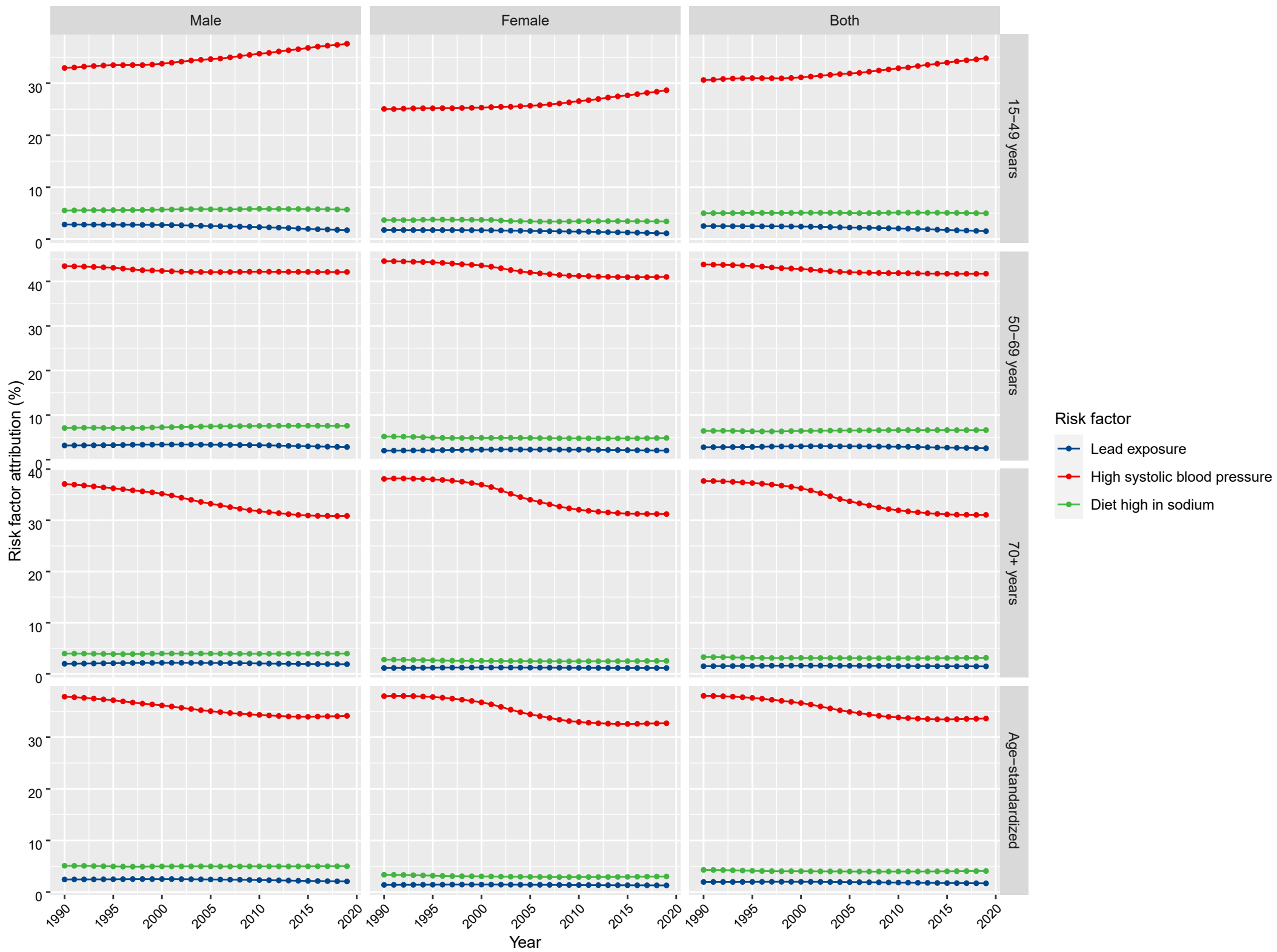

Supplement: Supplementary file 1 [file jcm-11-06733-s001.zip › Figure S3.pdf]

D

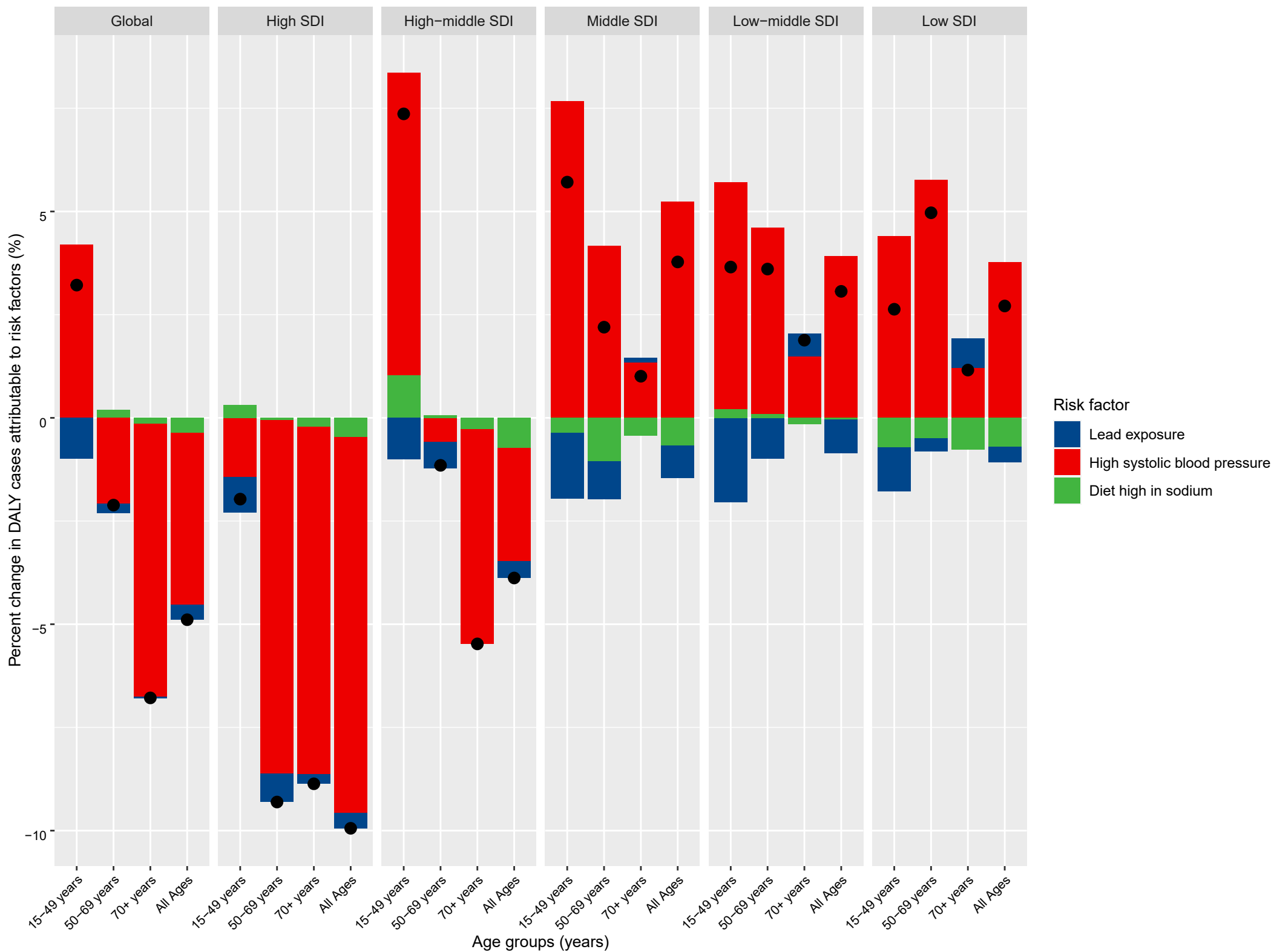

Supplement: Supplementary file 1 [file jcm-11-06733-s001.zip › Figure S4.pdf]
